# Temporal changes in fecal swine microbiome primarily reflect *Salmonella* Typhimurium challenge and poor sanitary housing conditions, even with functional amino acid supplementation

**DOI:** 10.3389/fvets.2025.1597857

**Published:** 2025-07-23

**Authors:** Antonio Diego Brandão Melo, Graziela Alves da Cunha Valini, Qinnan Yang, Marllon José Karpeggiane de Oliveira, Danilo Alves Marçal, Pedro Righetti Arnaut, Ismael França, Cleslei Alisson Silva, Nate Korth, Natasha Pavlovikj, Paulo Henrique Reis Furtado Campos, Henrique Gastmann Brand, John Kyaw Htoo, Andrew K. Benson, Luciano Hauschild, Joao Carlos Gomes-Neto

**Affiliations:** ^1^Department of Animal Science, School of Agricultural and Veterinary Sciences, State University of São Paulo (UNESP), Jaboticabal, Brazil; ^2^Department of Food Science and Technology, University of Nebraska-Lincoln, Lincoln, NE, United States; ^3^Nebraska Food for Health Center, University of Nebraska-Lincoln, Lincoln, NE, United States; ^4^Holland Computing Center, University of Nebraska-Lincoln, Lincoln, NE, United States; ^5^Department of Animal Science, Federal University of Viçosa, Minas Gerais, Brazil; ^6^Evonik Brazil Ltda, São Paulo, Brazil; ^7^Evonik Operations GmbH, Hanau, Germany; ^8^Department of Animal Sciences, The Ohio State University, Center for Food Animal Health, Wooster, OH, United States

**Keywords:** swine, microbiome, *Salmonella* Typhimurium, amino acids, sanitary condition

## Abstract

Nutrition has a significant impact on the gastrointestinal (GI) microbiome, which can influence pig metabolism, nutrient absorption, biomolecule synthesis, and bioavailability (including bile acids and short-chain fatty acids), as well as colonization resistance to GI pathogens and overall disease tolerance through immune maturation and regulation. The aim of this study was to assess the impact of functional amino acid supplementation on the fecal microbiome of pigs allocated into GOOD vs. POOR sanitary conditions (SC) over time, using 16S rRNA data. A total of 120 female growing pigs were randomly assigned in a 2 × 2 factorial arrangement (n = 30/treatment), consisting of two sanitary conditions (GOOD vs. POOR) and two diets [control (CN; 100% NRC, 2012) vs. supplemented with AA (Trp, Thr, and Met+Cys: Lys ratios increased to 20% higher than CN)]. Pigs were allocated to the GOOD SC group and were sham-inoculated, and the barn was kept clean, whereas pigs housed under POOR SC were challenged with *Salmonella* Typhimurium, in addition to the spreading fecal material from a commercial farm undergoing poor growth performance. Fecal samples were collected at day post-challenge (DPC) 0, 10, and 21, and extracted DNA was sequenced for 16S rRNA data analysis. Although alpha-diversity analysis revealed minor, statistically significant changes between groups, beta-diversity analysis demonstrated a significant separation between communities based on sanitary conditions at DPC 21. Accordingly, the most important taxa differentiating the two groups were the enrichment of the following taxa in the POOR group at DPC 21: *Clostridium sensu stricto 1*, *Dorea*, *Intestinibacter*, *Lactobacillus*, *Romboutsia*, *Ruminococcus torques*, *Subdoligranulum*, *Terrisporobacter*, and *Turicibacter*. Network and correlation structural analysis further revealed a sub-structuring of the data, with positive correlations forming in the POOR SC group: Sub-cluster 1 (*Romboutsia*, *Turicibacter*, *Clostridium sensu stricto 1*, *Terrisporobacter*, and *Intestinibacter*) and Sub-cluster 2 (*Dorea*, *Subdoligranulum*, *Ruminococcus torques*, *Blautia*, *Holdemanella*, and *Solobacterium*). In conclusion, temporal changes in the fecal swine microbiome of growing pigs reflected the *S.* Typhimurium challenge and poor sanitary status despite a dietary surplus of functional amino acids.

## 1 Introduction

Providing optimal nutrients in swine diets can maximize performance and profitability by improving animal health, potentially through modulation of the gastrointestinal (GI) microbiome (1–3). However, environmental and sanitary factors can affect feed intake, nutrient utilization, and lean meat deposition in modern pig genotypes (4–8). Various feeding and dietary programs, including increased supplementation with functional amino acids (AAs) with or without lowering crude protein, are typically deployed to mitigate the negative impacts of poor sanitary housing conditions and infectious, thereby maximizing pig growth (9–15).

The swine GI microbiome contributes to overall health by participating in, but not limited to, fiber digestion, vitamin and other micronutrient metabolism and biosynthesis (short-chain fatty acids—SCFA and bile acids), nutrient absorption, host metabolism, innate and adaptive immune development and maturation, and colonization resistance against enteric pathogens (2, 3, 16–22). Although the swine GI microbiome is biogeographically variable, growing pigs have a predictable composition at the genus level using 16S rRNA sequencing analysis, with *Acidaminococcus*, *Blautia*, *Clostridium*, *Fusobacterium, Intestinibacter*, *Lactobacillus*, *Megasphera*, *Oscillospira, Prevotella*, *Roseburia*, *Ruminococcus*, *Streptococcus*, *Subdoligranulum*, *Terrisporobacter*, *Treponema*, and *Turricibacter* being among the most dominant fecal taxa (16, 23–25). Many of these core beneficial groups of the swine fecal microbiome are colonizers of the ileum and/or large intestine, with some involved in SCFA production that can, in turn, impact intestinal epithelial health, immune function, and host basal metabolism (1, 26–30). Risk factors that are known for negatively affecting growing pigs’ performance, such as poor sanitary housing conditions and *Salmonella enterica subsp*. *enterica* lineage I serovar Typhimurium (henceforth named *S.* Typhimurium) intestinal infection is also associated with disruption of this core fecal microbiome, resulting in predictable changes in SCFA-producing bacteria (9, 11, 12, 22, 28, 31–37).

A surplus of functional amino acids has been used to enhance gut health, promote growth, and maximize feed efficiency in modern pigs (38). More specifically, dietary supplementation with threonine, methionine, and tryptophan has been shown to improve the average performance of *S.* Typhimurium-challenged pigs while supporting pigs’ health (9, 39–42), for instance, with an increase in core beneficial members of the swine microbiome such as *Lactobacillus*, *Clostridium*, and *Succinivibrio* (43, 44). Even though studies reporting methionine and threonine effects specifically modulating the swine GI microbiome are still scarce in the literature (3), a recent study demonstrated that *S.* Typhimurium seems to be capable of altering that nutrient metabolic partition by utilizing its virulence apparatus to induce AA (e.g., lysine) malabsorption in the ileum, resulting in a more favorable ecosystem for invasion in the large intestine, where the oxidative metabolism creates a bottleneck for SCFA producers (45).

Therefore, experimental studies assessing temporal changes in the GI microbiome (diversity, compositional structure, and differential taxa) of growing pigs challenged with an endemic enteric pathogen that poses a food safety concern, such as *S.* Typhimurium and raised under poor housing conditions mimicking commercial farms, supplemented with functional AA, pose an opportunity to identify novel, ecologically driven (host-adapted) synbiotic strategies that would help maximize pig performance. Therefore, the current study was designed to assess the impact of increased dietary supplementation with threonine, methionine, and tryptophan on the fecal microbiome over time on group-housed growing pigs under challenging sanitary conditions, expecting specific alterations in the core beneficial members of that microbiota directly linked to SCFA production underlying the microbiome resilience capacity and functional adaptation to *S.* Typhimurium challenge along with exposure to poor hygiene.

## 2 Materials and methods

### 2.1 Animal study and housing

A total of 120 female pigs (Pietrain × [Large White × Landrace]) with an initial body weight (BW) of 25.4 ± 3.7 kg were used in this experimental study. Pigs were sourced from SEARA Foods (SEARA Alimentos), Santa Catarina, Brazil. The pigs were distributed in a randomized block design in a 2 × 2 factorial arrangement composed of two sanitary conditions (Barn-SC): good (GOOD) or poor (POOR—*S.* Typhimurium inoculation + poor hygiene) and were fed with one of two diets [control (CN), formulated to meet the nutritional requirements according to (85); or surplus supplemented (AA) with Thr, Met+Cys, and Trp ratios to Lys adjusted to 20% above the CN diet], as previously described (9). The experiment was conducted in two similar barns located at the facilities of the Swine Research Laboratory at São Paulo State University (UNESP-Jaboticabal, São Paulo, Brazil). Pigs were identified and randomly assigned to one of two similar growing-finishing barns (0.9 m^2^/pig), where they had been housed for 14 days in the same facilities prior to the beginning of the experiment. The full concrete floor of both barns underwent the same cleaning and disinfection protocol before allocating all pigs. For both barns, the temperature was controlled (set to 22°C) with the aid of exhaust fans and an automated evaporative system of pad cooling (Big Dutchman, Araraquara, SP, Brazil) and monitored using data loggers (Hobo, Onset Computer Corporation, Bourne, MA, United States). Artificial lights were used to maintain a 12-h photoperiod (7:00 a.m.–7:00 p.m.). Additionally, each room was equipped with four Automatic and Intelligent Precision Feeders (AIPF, University of Lleida, Lleida, Spain) and six nipple drinkers, allowing *ad libitum* access to feed and water, respectively. AIPF’s feed delivery is achieved through recognition of the individual radio frequency ear tag (Allflex, Joinville, SC, Brazil) attached to the right ear of each pig, enabling individualized data collection, as previously described (9, 46).

### 2.2 Experimental challenge

The *S.* Typhimurium strain (RLO971/09, originating from a past swine clinical salmonellosis case) was used to prepare the experimental inoculum. This strain is stored at the Laboratory of Ornitopathology, Department of Veterinary Pathology, UNESP-Jaboticabal, SP, Brazil (9, 11). A single colony was grown overnight to make a total volume of 5 mL of brain heart infusion (BHI, CM 1135, Oxoid, Thermo Fisher Scientific, NH, England) broth, which was used to inoculate 2 × 10^9^ colony-forming units (CFU) of *S.* Typhimurium selected for antibiotic resistance to nalidixic acid (25 μg/mL) by oral gavage in all 60 pigs allocated into the POOR (*S.* Typhimurium inoculation + poor hygiene) SC barn, as previously described (9). The counterpart pigs allocated in the GOOD SC barn were sham-inoculated via oral gavage with 5 mL of brain heart infusion broth solution as negative controls, following the same procedures done on pigs allocated in the POOR (*S.* Typhimurium inoculation + poor hygiene) SC barn.

Under both inoculation conditions, all pigs were fasted for 6 h and had no water consumption for 1 h prior to inoculation. After pigs in the POOR (*S.* Typhimurium inoculation + poor hygiene) SC barn was inoculated with *S.* Typhimurium, fresh manure from a commercial pig farm was spread across all pens to establish the initial sanitary challenge and remained uncleaned throughout the study, as previously described (9). In contrast, in the GOOD SC barn, no manure was spread on the floor, while it was cleaned twice a day with a water jet stream, and potassium monopersulfate (1:200; Virkon; Lanxess, Colony, Germany) was pulverized into the air once a week as part of the biosecurity protocol to improve the hygiene condition. The management teams were required to wear clean clothing and footwear with a bleach solution (1:10 dilution) when entering the building to avoid cross-contamination (9). Regardless of sanitary conditions, pigs did not receive any growth promoters or any other treatment prior to or during the experiment. Cross-contamination between barns was assessed by testing fecal samples at day post-challenge (DPC) 0, 10, and 21 for *Salmonella* spp. growth using selective media (9), and none was found. All experimental procedures applied in this trial followed the guidelines of the Brazilian National Council of Control of Animal Experimentation (CONCEA) and were reviewed and approved [protocol no. 4784/20] by the Ethical Committee on Animal Use (CEUA) of Faculdade de Ciências Agrárias e Veterinárias (FCAV/UNESP—Jaboticabal, SP, Brazil) (9).

### 2.3 Experimental diets

As previously described (9), all experimental diets were based on corn-soybean meal and were formulated using the reported nutrient content and analyzed AA composition of ingredients to meet or exceed the nutrient requirements based on pig weight at the beginning of the study, according to (85) and AMINODat^®^ 6.0 (Evonik Operations GmbH, Essen, Germany). AMINODat is a web-based, internationally referenced database for the nutritional composition of feed ingredients. The diets were steam-pelleted (2.5 mm) and provided *ad libitum* without the inclusion of in-feed antibiotics as growth promoters throughout the trial.

### 2.4 Sample collection, DNA extraction, 16S rRNA amplicon sequencing, and bioinformatic processing

Rectal samples were collected from all pigs at DPC 0, 10, and 21. Specifically at DPC 0, fecal samples were collected prior to *S.* Typhimurium inoculation, and sample aliquots were separated for bacterial counts (fresh samples) and DNA extraction for subsequent 16S rRNA sequencing. All aliquots used for DNA extractions were frozen in liquid nitrogen and then stored at −80°C. DNA extraction was performed using the Qiagen DNeasy® PowerSoil® Pro kit in accordance with the manufacturer’s instructions (12). All DNA extracts were eluted in 60 μL of elution buffer and then frozen at −80°C prior to quality assessment and sequencing. DNA concentration was checked using a Nanodrop One spectrophotometer (Thermo Fisher Scientific Inc., Middletown, VA). For 16S rRNA sequencing, the V4 region was amplified from each sample using an in-house modified dual-indexing sequencing strategy on the Illumina MiSeq platform, as previously described (12, 47, 48). Paired-end sequences were analyzed using the Quantitative Insights Into Microbial Ecology (QIIME) program (version 2) (49). Sequences were truncated (220 bases for forward reads and 160 bases for reverse reads) and denoised into amplicon sequence variants (ASVs) using DADA2 (50). They were then rarefied to 5,000 reads per sample, as previously done (12). All ASVs were assigned taxonomic information using the pre-fitted sklearn-based taxonomy classifier from the SILVA database (release 138) (12, 48, 51). Only bacterial taxa (contained domain = “Bacteria”) with genus-level information in the name (g from QIIME2 output) were filtered for both diversity and taxonomic analyses prior to statistical analysis. Each fecal sample was treated as a unique experimental unit for microbiome sequencing with no replicates added.

### 2.5 Co-occurrence network analysis of fecal microbiome data

Network construction and visualization were performed using the NetCoMi package within the R statistical framework (version 4.2.2) (52). Overall, samples were rarefied to 5,000 reads and grouped by sampling at DPC 21 and sanitary conditions [GOOD vs. POOR (*S.* Typhimurium inoculation + poor hygiene)], as previously described (12). In brief, each network was constructed using a centered log-ratio transformation and Spearman-based correlations, employing the 50 most abundant microbial taxa. Data interpretation was summarized based on each node representing a bacterial taxon, and the size of each node is scaled according to its centrality. Edges represented associations between taxa, with positive correlations being colored in green and negative ones in red. The thickness of each edge corresponds to the strength of the association, and edges representing a value less than 0.5 were excluded from the analysis. Taxa were colored based on clusters calculated in network construction. Therefore, the taxa with the highest degree of centrality were selected to highlight cluster-specific dominance (12).

### 2.6 Statistical analyses

All bacterial taxonomic outputs were processed for quality control (data distribution) to statistical modeling using R version 4.4.0. All data exploration and statistical analysis were conducted using bacterial genus-level taxonomy only, except for an examination of the most important taxa (>1%) over time, which was also performed using family-level taxonomic resolution when necessary for data amalgamation. In brief, the tidyverse library (version 2.0.0) was used for data exploration, analysis, and plotting. Statistical analysis was conducted at two levels: (1) by comparing Barn-SC GOOD vs. POOR (*S.* Typhimurium inoculation + poor hygiene) by DPC (in the absence of interaction between diet and Barn-SC, with Barn-SC being the main significant effect); and (2) by comparing all treatments [combination between Barn-SC and diet by DPC—GOOD CN, GOOD AA, POOR (*S.* Typhimurium inoculation + poor hygiene) CN, and POOR (*S.* Typhimurium inoculation + poor hygiene) AA]. All taxa data were rarefied to 5,000 counts, as previously described (12). A first-glance analysis of the sampling distribution using mean and median was conducted for taxa with a proportion greater than 1% (major taxa) at both the family and genus levels (hierarchical levels) by DPC, regardless of treatments, to assess data skewness. For alpha-diversity (Simpson’s D and Shannon’s indexes), either a one-way analysis of variance (ANOVA) (more than two treatments—Barn-SC, dietary condition, and interaction) or a Welch T-test (two treatments only—Barn-SC effect, regardless of diet provided) was used to assess significance (*p* < 0.05). Both Shannon and Simpson’s D indexes of alpha diversity were calculated using rarefied counts, with the diversity function in R from the vegan library (version 2.6-6.1).

If no significant effects arose from the interaction between Barn-SC and Diet, only the significant main effect would be further examined. This approach was taken for alpha- and beta-diversity. The rest of the analysis was conducted based on the main significant effect on community diversity, composition, and structure, as previously established by Gomes-Neto et al. (32).

Beta-diversity was calculated using the vegdist function from the vegan library (version 2.6-6.1), employing a Bray-Curtis distance matrix (non-binary data) within a framework previously established (32). For the principal coordinates analysis (PCoA), a classical multidimensional scaling model was first used to reduce the distance matrix to two dimensions (2 principal coordinates—PCs), using the cmdscale function (k = 2) in R from the stats library. Specifically, for DPC 21, principal component analysis (PCA) was employed to reduce the data to three dimensions, thereby enhancing the clustering assessment. Ultimately, PC1 and PC2 were used to present a biplot that captures the effects of either sanitary conditions or all treatment effects on beta diversity each day. A PERMANOVA was used to calculate the effect of treatment groups (*p*-value and R-squared) on beta-diversity (Bray-Curtis distances), using the ‘adonis2’ function in R from the “vegan” library (version 2.6-6.1). A volatility analysis (microbiome compositional assessment using each PC independently as an outcome variable—PC1, PC2, or PC3 from the PCoA) was conducted by assessing significant differences between Barn-SC groups across each DPC or across all treatments by DPC as well (a Welch T-test was used to compare Barn-SC by DPC); an ANOVA model was used for all treatments by DPC, with a Tukey Honest Significant Differences test (Tukey HSD) applied for pairwise comparisons, provided a significant effect of treatments (*p* < 0.05) was found in the ANOVA model. An analysis of similarity (ANOSIM) was used to calculate the effect of treatment groups (Barn-SC or all treatments) on beta-diversity (Bray-Curtis distances), using the anosim (method = “bray”—Bray-Curtis’s distance matrix) functions in R, respectively, from the vegan library (version 2.6-6.1). Finally, a PCA (without a distance matrix) analysis was also conducted using log2-transformed proportions across bacterial taxa, with rarefied counts as input data, to assess clustering between Barn-SC groups and across all treatments by DPC.

Keystone taxa were identified based on significant (*p* < 0.05) differences in community structure at DPC 21 across Barn-SC groups [GOOD vs. POOR (*S.* Typhimurium inoculation + poor hygiene)]; by filtering all bacterial genera with (1) mean proportion > 1%; (2) using a LEfSe analysis; (3) using a co-occurrence network analysis to identify central nodes; and (4) using a random forest classifier. Mean and median proportions were compared for skewness, and mean proportions were used to examine the distribution of keystone taxa using either proportion or log2-transformed proportions (adding a pseudo-value of 1 for all data to ensure proper transformation with zero counts). LEfSe analysis for identification of differentiating taxa was done with the Galaxy platform with a Wilcoxon (*p* < 0.05), and upon removal of taxon or features with zero counts across fecal samples, only contrasting sanitary conditions [GOOD vs. POOR (*S.* Typhimurium inoculation + poor hygiene) Barn-SC] were considered (53). The random forest classifier was also applied for DPC 0 and 10. For this purpose, the caTools and randomForest libraries were utilized in R. All random forest models were constructed using rarefied counts (without further transformation), 100 trees, a classification model for a binary outcome, and a 70/30 random split of the training and testing data, respectively. The Gini importance was used to identify the most important predictors of the random forest model, which differentiates between POOR (*S.* Typhimurium inoculation + poor hygiene) and GOOD Barn-SC across each DPC.

Statistical differences across keystone taxa were calculated using both proportion and log2-transformed proportion, all derived from rarefied counts. When comparing GOOD vs. POOR (*S.* Typhimurium inoculation + poor hygiene), Barn-SC, a Welch T-test (*p* < 0.05), was used by DPC. If comparing across all four treatment groups, then an ANOVA model was used, followed by a pairwise T-test, provided the ANOVA model yielded significant results (*p* < 0.05). Box-and-whiskers and a clustering heatmap were used to visualize the distribution of taxa across Barn-SC groups or all treatments by DPC. For the clustering heatmap visualizations, a nested approach was applied, considering the 2 × 2 factorial design (diet groups within each barn-SC). All cluster heatmap analysis was performed using log2-transformed proportions (a pseudo-value of 1 was input when zero was present), followed by data scaling (normalization) and visualization using a heatmap.2 functions in R. The correlation structure between keystone taxa was also calculated using log2-transformed proportions, with input values of 1 for zeros. This analysis was conducted using the cor function to calculate the Pearson correlation matrix, followed by the cor.mtest to identify significant correlations (*p* < 0.05). The results were then visualized using a corrplot in R, clustered using a hierarchical clustering approach (hclust). Fold-change (ratio) differences between keystone taxa across Barn-SC or all treatments were made from the endpoint (DPC 21) and baseline (DPC 0). For that, a pseudo-value of 0.01 was initially input for zeros in the mean proportional data (to avoid infinity values in the denominator), followed by a log_2_ transformation of the ratio with a pseudo-value imputation of 1 for zeros. Pearson’s correlation between keystone taxa relative abundance (log_2_ transformed) ratio (DPC 21/DPC 0) and the average daily gain (host-associated trait) for the corresponding period was also calculated. All tests for Pearson’s correlation coefficient were calculated using the cor_test function in R (*p* < 0.05). For the average daily gain between DPC 0 and 21 (host-associated trait), an ANOVA model was used to assess the effect of diet, Barn-SC, and interaction between the two (*p* < 0.05). All ANOVA models were done using the aov function in R, while two-group comparisons were made using a T-test by applying a two-sample t-test function in R from the stats library. A sample was removed from the analysis when a missing value (NA) was consistently present across all taxa. Illustrative figures were created using BioRender.

## 3 Results

### 3.1 Experimental background and workflow

**Figure 1:**
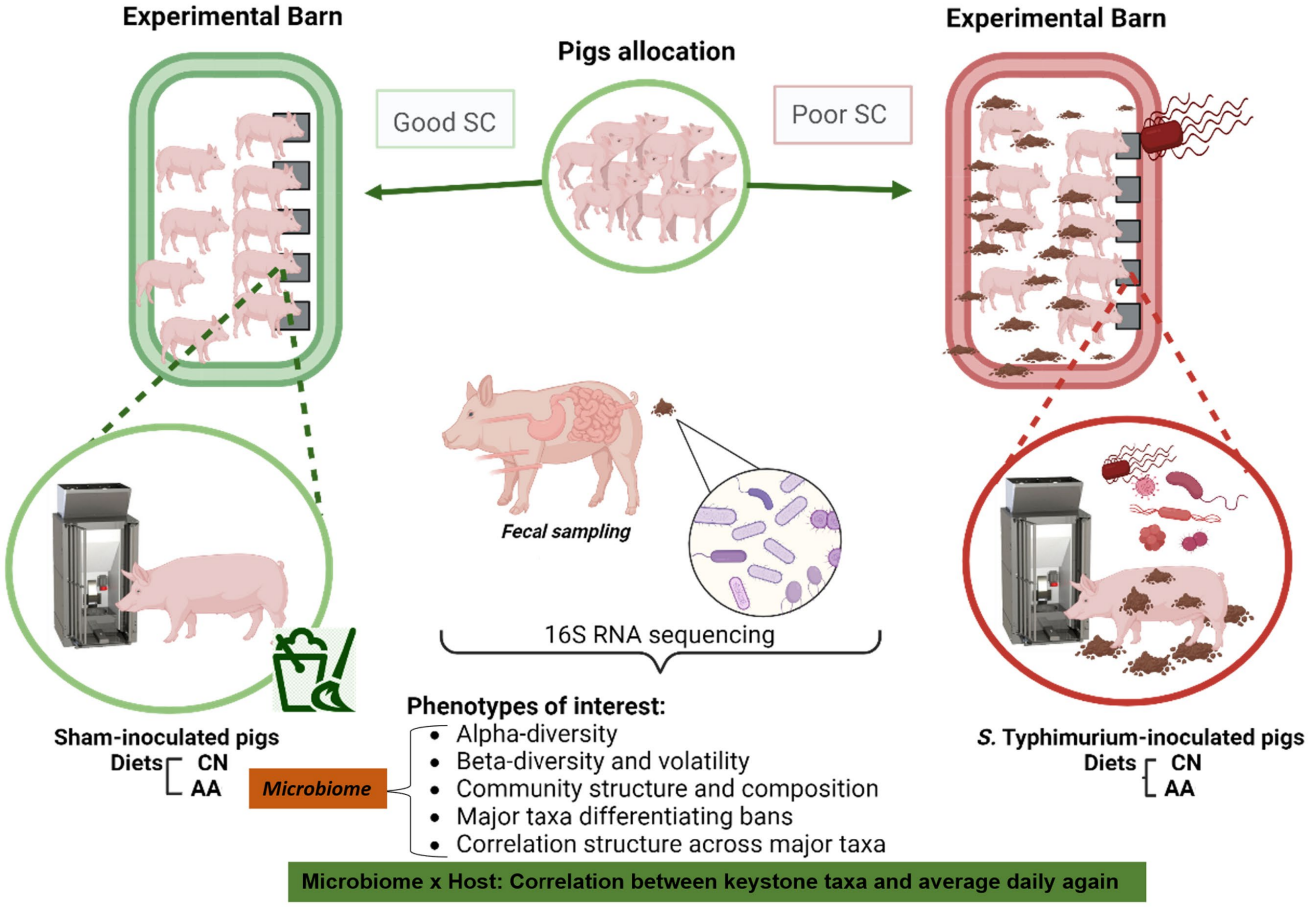
Experimental design explaining the experimental and analytical workflow. The experimental workflow describes the random allocation of female, age-matching, growing pigs into two sanitary conditions: GOOD vs. POOR (*S.* Typhimurium inoculation + poor hygiene) (Barn-SC). Fecal samples were longitudinally collected from animals at DPC 0, 10, and 21. The experiment employed a 2 × 2 factorial design, consisting of two housing sanitary conditions (Barn-SC: GOOD vs. POOR) and supplementation, or not, of conventional diets with functional amino acids (AA) (Trp, Thr, and Met+Cys: Lys ratios increased to 20% higher than control). Animals allocated under GOOD sanitary conditions were sham-inoculated, whereas animals housed under POOR sanitary conditions were challenged with *S.* Typhimurium, in addition to using fresh stools collected from a herd with low sanitary status and spread on the floor at DPC 0. Fecal microbiome samples were analyzed using 16S rRNA sequencing for subsequent diversity, community structure, and composition analysis, along with a taxa enrichment analysis to identify differences across treatments (microbiome-associated ecological attributes). Additionally, a fold change in the relative abundances of keystone taxa was correlated with the average daily gain throughout the study (DPC21/DPC0) (Microbiome × Host-associated trait). The fecal sample size for these analyses is presented in Supplementary Table 1A,B. Each animal was considered an experimental unit throughout the analysis.

In our companion study, several aspects of growth, including performance and body composition, among others, were captured in group-housed growing pigs under contrasting sanitary conditions [GOOD SC vs. POOR (*S.* Typhimurium inoculation + poor hygiene) SC] and fed two diets, control (CN) or supplemented with AA (9). Pigs housed under POOR SC (*S.* Typhimurium inoculation + poor hygiene) had a significantly lower average daily gain compared to those in GOOD SC (*S.* Typhimurium inoculation + good hygiene) (*p* < 0.05), underscoring a potential difference in fecal microbial communities. Figure 1 presents the experimental design, sampling, and key microbiome outcomes. Supplementary Table 1A,B describe the fecal sampling scheme throughout the study across all treatments. More specifically, this study assessed microbial diversity, community structure, and enrichment of differentiable taxa across treatments over time using 16S rRNA sequencing from fecal samples. The aim was to identify differences between sanitary conditions and the potential impact of AA surplus in the diets of growing pigs. A first-glance assessment of the keystone fecal taxa (> 1%) both at the family and genus level, over time, regardless of treatment conditions, was done to determine data skewness (mean vs. median) prior to subsequent statistical data mining with all samples rarefied at 5,000 counts (Supplementary Figures 1, 2).

### 3.2 Alpha-diversity

**Figure 2:**
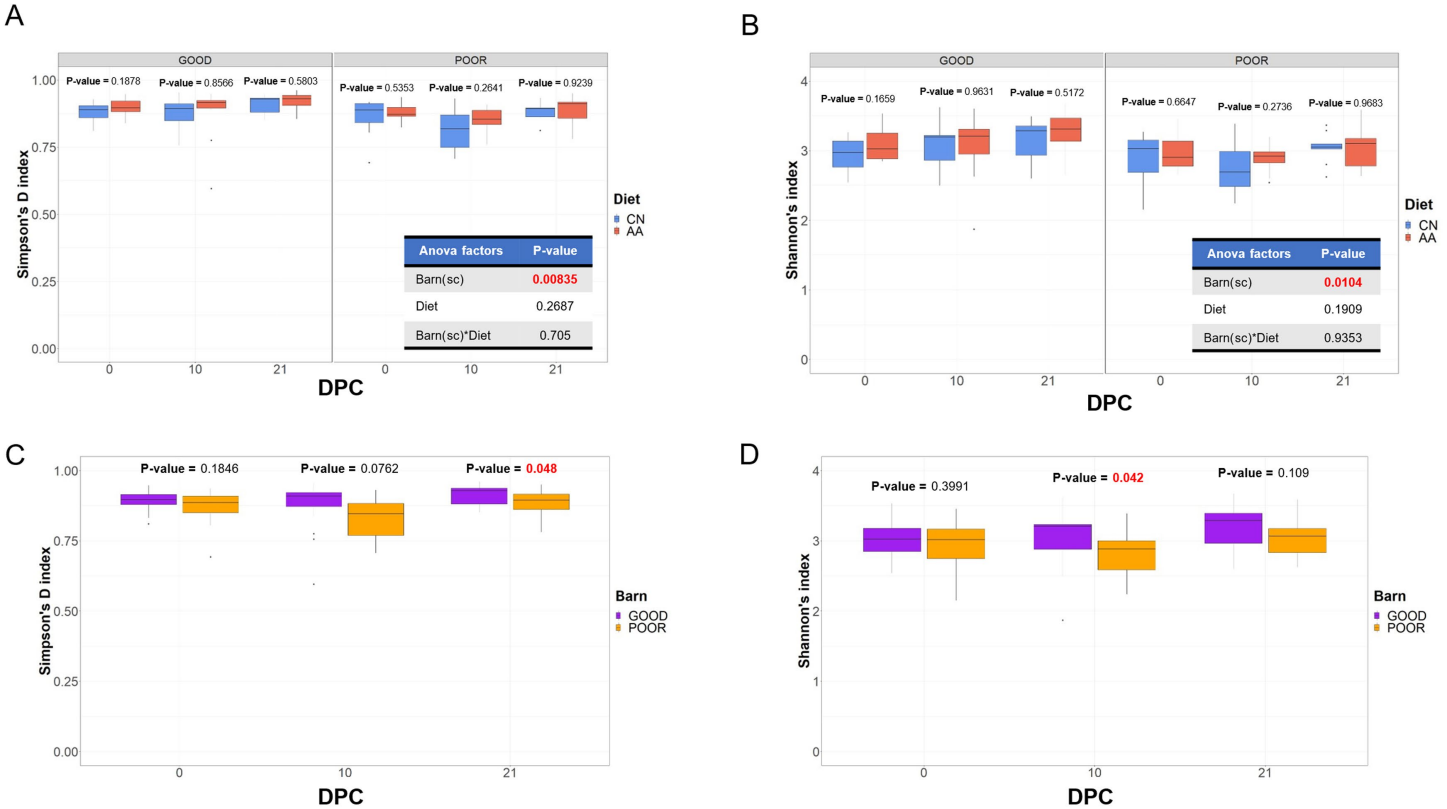
Alpha-diversity analysis of the fecal microbiome composition across treatment days post-challenge. **(A,B)** Simpson’s D and Shannon’s indexes of diversity are analyzed across diets (CN, conventional; AA, supplemented with functional amino acids) based on housing sanitary conditions (Barn-SC), respectively. **(C,D)** Simpson’s D and Shannon’s indexes of diversity are analyzed across barns with either GOOD or POOR (*S.* Typhimurium inoculation + poor hygiene) sanitary conditions. A one-way ANOVA was used to assess the effects of diet, barn, and their interaction on both Simpson’s and Shannon’s indexes of alpha-diversity (*p* < 0.05) **(A,B)**. Given the significant effect of “Barn(sc)” (housing sanitary condition) (*p* < 0.05) **(A,B)**, a follow-up analysis (**C,D**) was conducted across DPC time points, comparing sanitary housing groups (GOOD vs. POOR). A two-sided T-test was used for comparison across two groups in all analyses (*p* < 0.05). *p*-values marked in red represent a significant effect for that comparison (*p* < 0.05). All alpha-diversity metrics were calculated using rarefied count data.

Differences in alpha diversity across treatments were analyzed using both the Simpson’s D (evenness) and Shannon (richness) indexes of diversity. Figures 2A,B demonstrates the results of the ANOVA and T-test-based differences across diet groups by DPC for each SC condition for both Simpson and Shannon indexes, respectively. Significant differences were found for both Simpson (*p* = 0.00835) and Shannon (*p* = 0.0104) indexes of diversity only in barn SC (Figures 2A,B), with no significant differences between diet groups across SC by DPC (*p* < 0.05). Alpha diversity was significantly lower in the POOR SC group (*S.* Typhimurium inoculation + poor hygiene) at both DPC 21 (*p* = 0.048) using Simpson’s D index and at DPC 10 (*p* = 0.042) using the Shannon index, in comparison to the GOOD SC group (Figures 2C,D).

### 3.3 Beta-diversity analysis

**Figure 3:**
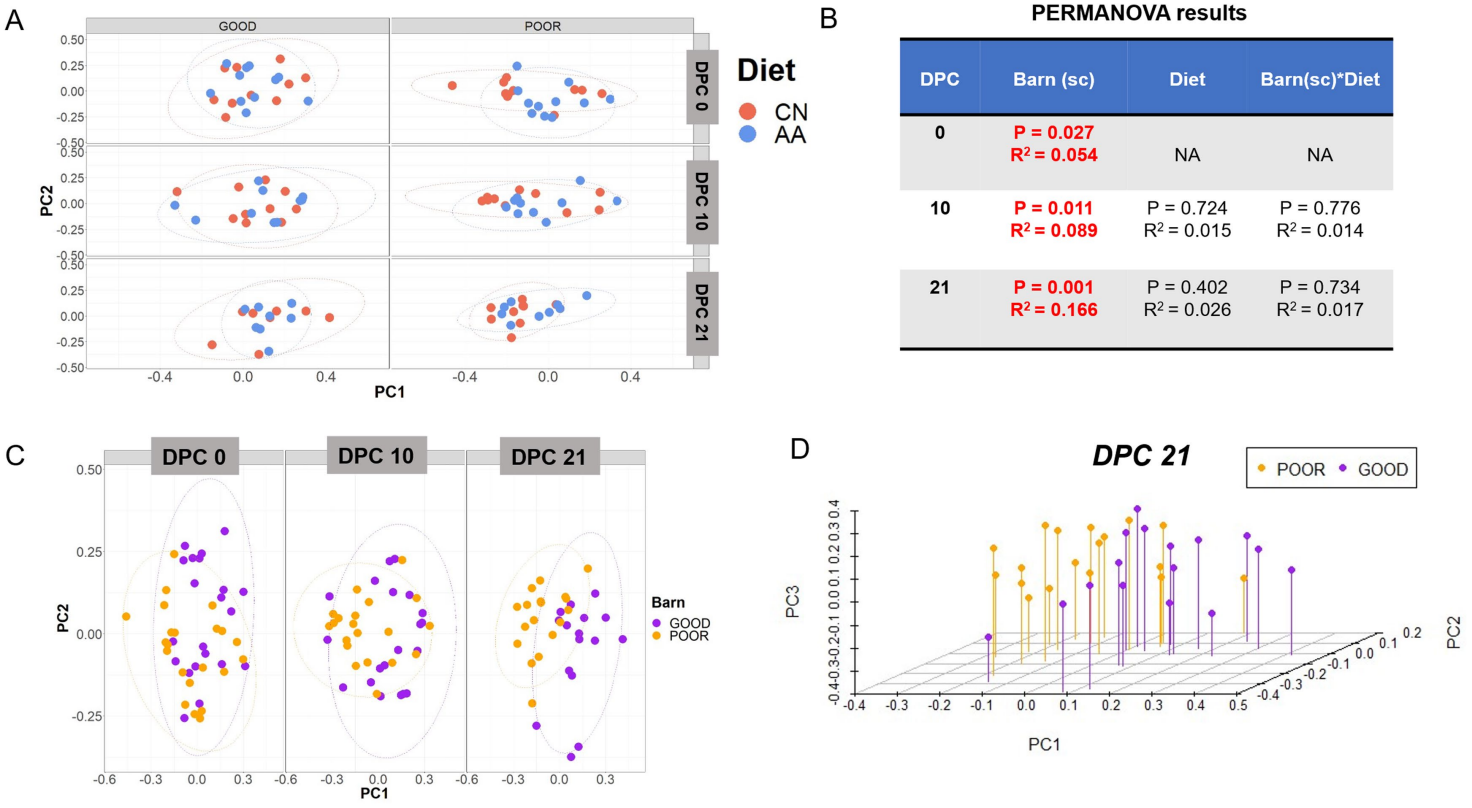
Beta-diversity analysis of fecal microbiome composition across diets (CN vs. AA) and housing sanitary conditions (GOOD vs. POOR—*S.* Typhimurium inoculation + poor hygiene) post-challenge. **(A)** Two principal coordinates (PC) are shown across diet and/or barns at DPC 0, 10, and 21. **(B)** A PERMANOVA model was used for variance decomposition across experimental factors, including diet and housing sanitary conditions (Barn-SC) and their interaction effects. PERMANOVA *p*-values and R-squared statistics are depicted with significant differences highlighted in red (*p* < 0.05). **(C)** Beta-diversity analysis was conducted for the “Barn-SC” effect across DPC 0, 10, and 21. **(D)** A three-dimensional visualization of the principal coordinate analysis for the housing sanitary effect is provided. The Bray–Curtis distance matrix was used to calculate beta diversity between diets (CN vs. AA) and/or barns under two distinct sanitary conditions (GOOD vs. POOR). Beta diversity analysis was executed using rarefied count data.

Initial assessment of beta diversity across fecal communities showed no clustering by diet across SC conditions by DPC (Figure 3A). A PERMANOVA analysis revealed only a significant effect for barn SC (*p* < 0.05) and not for diet or the interaction between the two, with the R-squared going from ~ 5% at DPC 0 to ~17% at DPC 21, demonstrating a significant biological effect of SC on the community clustering (Figure 3B). Based on this, Figure 3C highlights a clear separation of the fecal communities by SC, starting at DPC 10, with more discrete clustering at DPC 21. A 3D representation of PCoA analysis at DPC 21 confirmed the separation between GOOD vs. POOR (*S.* Typhimurium inoculation + poor hygiene) SC fecal communities (Figure 3D). ANOSIM results mirrored those of the PERMANOVA, underscoring a dissimilarity between GOOD vs. POOR (*S.* Typhimurium inoculation + poor hygiene) SC fecal communities, starting at DPC 10 (R = 0.196, *p* = 0.01) and reaching a higher degree at DPC 21 (R = 0.352, *p* = 0.001) (Supplementary Figures 3–5). When stratified by all treatment combinations, ANOSIM results corroborated the differences between SC groups, with fecal community dissimilarities most pronounced at DPC 21, irrespective of diet (R = 0.219, *p* = 0.001) (Supplementary Figures 6–8). A follow-up analysis using the individual PCs (PC1, PC2, and PC3) as the response variable recapitulated the beta-diversity results, demonstrating significant differences across SC groups at DPCs 10 and 21, not driven by diet (*p* < 0.05) (Supplementary Figures 9A–D, 10). Notably, the POOR (*S.* Typhimurium inoculation + poor hygiene) SC community exhibited higher dispersal, as indicated by PC1- and PC3-based analysis (Supplementary Figures 9A, 10A). An independent PCA analysis (not using a distance matrix) corroborated all results by showing clustering driven by barn SC at DPC 10 and 21 (Supplementary Figures 11–13) and not by diet (Supplementary Figures 14–16).

### 3.4 Taxa enrichment analysis, temporal changes, and correlation with host phenotype

**Figure 4:**
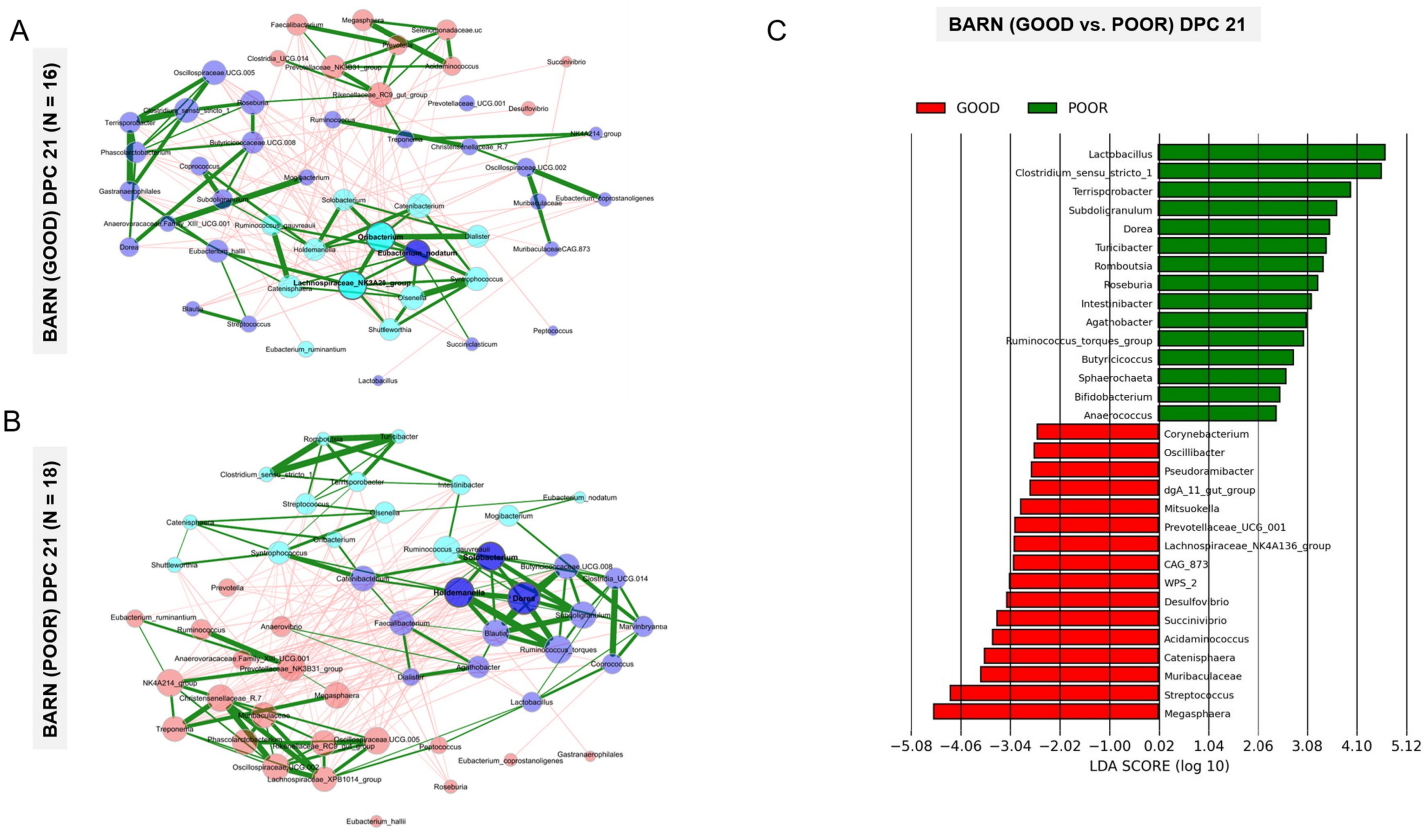
Community structure analysis complemented by linear discriminant analysis effect Size (LEfSe) of fecal microbiome samples from pigs housed under either GOOD or POOR (*S.* Typhimurium inoculation + poor hygiene) sanitary conditions at DPC 21. **(A,B)** Network analysis for examination of community structure patterns across fecal microbiome samples collected at DPC 21, highlighting the greatest beta-diversity difference between GOOD and POOR sanitary conditions (**Figure** **3****C**) for pigs housed under each condition, respectively. Each node represents a bacterial taxon, with the size of each node scaled according to its centrality. Positive Spearman-based associations between taxa are depicted as green edges (the thicker the line, the stronger the association), while red edges are indicative of an anti-correlation between taxa. Edges representing a value less than 0.5 are not shown. Taxa are colored based on clusters calculated during network construction. **(C)** LEfSe results for comparison between GOOD and POOR housing conditions at DPC 21. Both network and LEfSe analyses were used to identify core-microbiome taxa that differentiate pigs housed under GOOD vs. POOR sanitary conditions, as the strongest beta-diversity effect was found at DPC 21. Rarefied taxon counts and proportions (derived from rarefied count data) were used as raw input data for network and LEfSe analysis, respectively.

**Figure 5:**
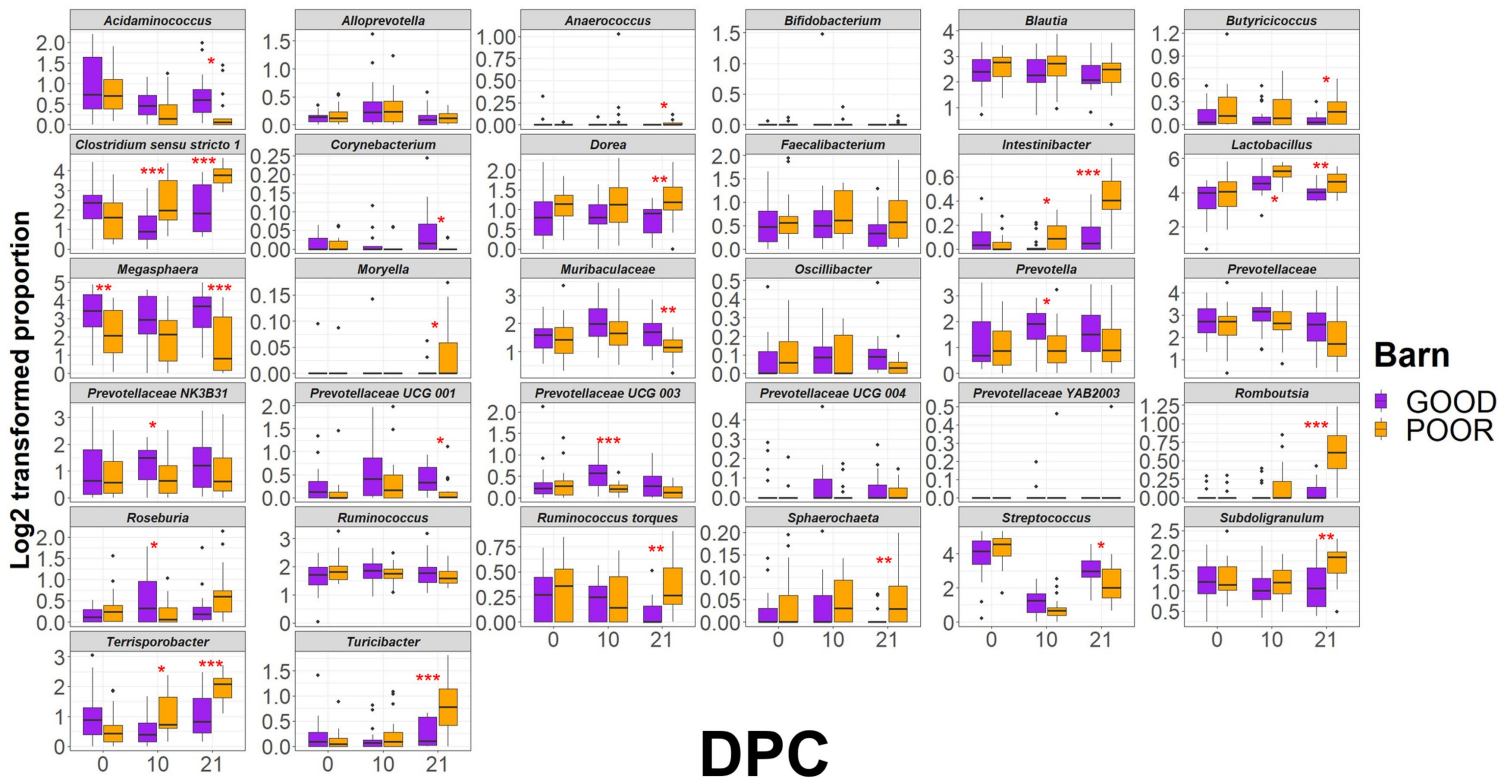
Distribution of keystone taxa across fecal samples from pigs housed under GOOD vs. POOR (*S.* Typhimurium inoculation + poor hygiene) sanitary conditions. The log2-transformed proportions of keystone taxa across sanitary conditions (Barn). A two-sided T-test was conducted to assess differences between housing sanitary conditions at each DPC (*p* < 0.05). * For *p* < 0.05; ** for *p* < 0.01, and *** for *p* < 0.001. Rarefied taxon counts transformed to proportions were used as raw data prior to log_2_ transformation.

Given the beta-diversity results clearly demonstrating a separation between fecal communities between SC conditions at DPC 21 (Figures 3B–D) and the absence of a significant dietary effect (Figure 3A), a combination of multiple analytical methods was used to identify all taxa differentiating GOOD vs. POOR (*S.* Typhimurium inoculation + poor hygiene) SC groups at DPC 21 first, including network analysis (Figures 4A,B), LEfSe analysis (Figure 4C and Supplementary Figures 17–23), random forest (Supplementary Figures 24–26), and t-test (Figure 5 and Supplementary Figure 29). Additionally, an exploratory analysis was conducted using the overall mean abundance and a cut-off of 1% to select the major taxa across the dataset (Supplementary Figures 1, 2), which allowed for contextual interpretation of the data for the most significantly different taxa between groups (*p* < 0.05).

Based on that, the most important taxa differentiating the two groups are as follows: (1) on average lower in relative abundance in the POOR (*S.* Typhimurium inoculation + poor hygiene) group at DPC 21: *Acidaminococcus*, and (2) on average, higher in relative abundance in the POOR (*S.* Typhimurium inoculation + poor hygiene) group at DPC 21: *Clostridium sensu stricto 1*, *Dorea*, *Intestinibacter*, *Lactobacillus*, *Romboutsia*, *Ruminococcus torques*, *Subdoligranulum*, *Terrisporobacter*, and *Turicibacter*. Network analysis revealed two unique sub-clustering structures (with positive interactions) formed in the POOR (*S.* Typhimurium inoculation + poor hygiene) SC group (Figure 4B): Sub-cluster 1 (*Romboutsia*, *Turicibacter*, *Clostridium sensu stricto 1*, *Terrisporobacter*, and *Intestinibacter*) and Sub-cluster 2 (*Dorea*, *Subdoligranulum*, *Ruminococcus torques*, *Blautia*, *Holdemanella*, and *Solobacterium*). LEfSe analysis also indicated enrichment in *Roseburia* for POOR (*S.* Typhimurium inoculation + poor hygiene) SC at DPC 21 (Figure 4C). Average community abundances (proportion or log-transformed) were also examined (Supplementary Figures 27, 28). Following the identification of the most differentiating taxa, a temporal analysis was conducted using relative frequencies (log-transformed or not) for the barn SC effect or across all treatments (Figure 5 and Supplementary Figures 29–31).

**Figure 6:**
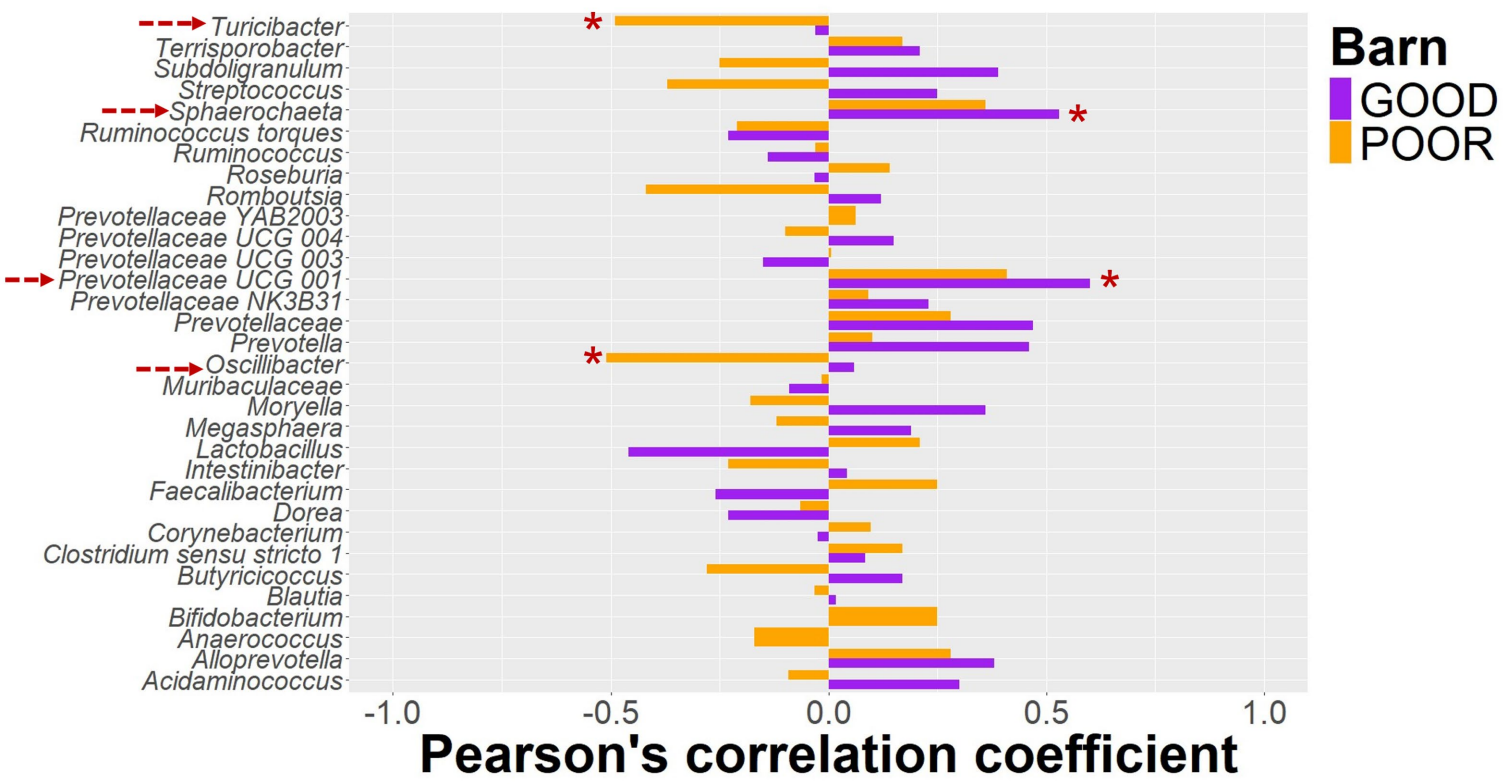
Pearson’s correlation between keystone taxa using log2 ratios from baseline to endpoint and average daily gain. Pigs were housed under either GOOD or POOR conditions (*S.* Typhimurium inoculation + poor hygiene) in sanitary conditions. The log2 transformed proportional ratio of keystone taxa across the sanitary conditions (barn) was used for the correlation analysis. All log2 transformed ratios were calculated using rarefied data as input, followed by calculating the relative abundance of each taxon by animal DPC 21 in relation to DPC 0. A value of 0.01 was assigned for proportions that were zero at DPC 0. The average daily gain between DPC 0 and 21 was used as the animal phenotype. Red dotted arrows on the left side mark all taxa with a significant Pearson’s correlation coefficient based on *p* < 0.05. * For *p* < 0.05; ** for *p* < 0.01, and *** for *p* < 0.001.

The initial clustering separation between GOOD vs. POOR (*S.* Typhimurium inoculation + poor hygiene) SC groups for the beta-diversity analysis at DPC 10 (Figure 3C) could be explained in part by the significantly higher average abundance for *Clostridium sensu stricto 1*, *Intestinibacter*, *Lactobacillus*, *and Terrisporobacter* in the POOR (*S.* Typhimurium inoculation + poor hygiene) SC group and significantly lower abundance for *Prevotella*, some *Prevotellaceae* taxa, and *Roseburia* in the same group (Figure 5 and Supplementary Figure 29), despite AA surplus (Supplementary Figures 30, 31). Additionally, a fold-change analysis demonstrated that *Clostridium sensu stricto 1*, *Intestinibacter*, *Romboutsia*, *Terrisporobacter*, and *Turicibacter* were among the top enriched taxa in the POOR (*S.* Typhimurium inoculation + poor hygiene) SC group at DPC 21 in relationship with DPC 0 (Supplementary Figure 32), despite AA surplus in the diet (Supplementary Figure 33). The host phenotype of interest, average daily gain, was significantly lower in the POOR (*S.* Typhimurium inoculation + poor hygiene) SC group and remained unchanged by AA surplus (Supplementary Figure 34). In the GOOD SC group, *Prevotellaceae UCG 001* and *Sphaerochaeta* were significantly positively correlated with average daily gain for the duration of the study (Supplementary Table 2A). On the other hand, *Oscillibacter* and *Turicibacter* were significantly negatively correlated with average daily gain for the study duration in the POOR (*S.* Typhimurium inoculation + poor hygiene) SC group, while no significant association was found for the GOOD SC for these two taxa (Figure 6 and Supplementary Table 2B). In aggregate, *Prevotella* was more likely to be positively correlated with the host phenotype for the GOOD SC group (Supplementary Table 1A,B). When stratifying by barn SC, it was found that for GOOD CN, *Moryella* and *Sphaerochaeta* were significantly positively correlated with the outcome (Supplementary Table 3A), while *Lactobacillus* was significantly negatively correlated with it for GOOD AA (Supplementary Table 3B). Finally, for POOR (*S.* Typhimurium inoculation + poor hygiene), AA *Prevotellaceae UCG 003* was found to be significantly positively correlated with average daily gain, while no significant associations were found across taxa for POOR (*S.* Typhimurium inoculation + poor hygiene) CN, even though both *Romboutsia* and *Turicibacter* trended in demonstrating a strong negative correlation in comparison to POOR AA (Supplementary Table 4A,B).

### 3.5 Temporal clustering and correlation-based analysis for the most differentiable taxa

**Figure 7:**
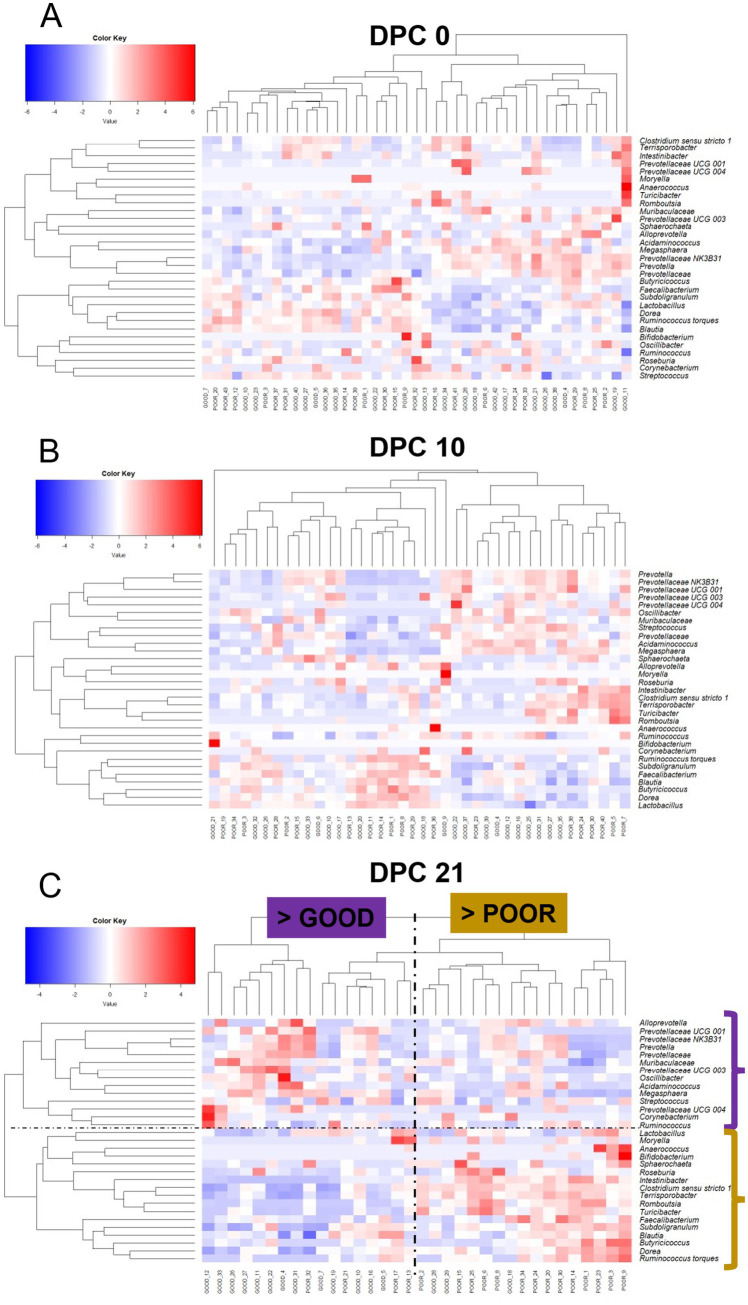
Keystone-based heatmap clustering analysis demonstrating changes in fecal microbiome composition across DPC 0 **(A)**, 10 **(B)**, and 21 **(C)** for pigs housed either at GOOD or POOR (*S.* Typhimurium inoculation + poor hygiene) sanitary conditions. Plot **C** is marked with a vertical dotted line to demonstrate a clustering separation between barn GOOD vs. POOR (> representing that the majority of pigs in that group belonged to the respective barn group) and a horizontal dotted line to show the taxa differentially enriched between groups (with two brackets on the side—color-matching pattern based on barn). Each row represents a distinct taxon, while each column represents an individual pig housed in barns under GOOD or POOR sanitary conditions. The input data consisted of rarefied proportions that were log2-transformed prior to scaling (deviation from the mean divided by the standard deviation of the sample). The scaling transformation was applied simultaneously across all taxa.

**Figure 8:**
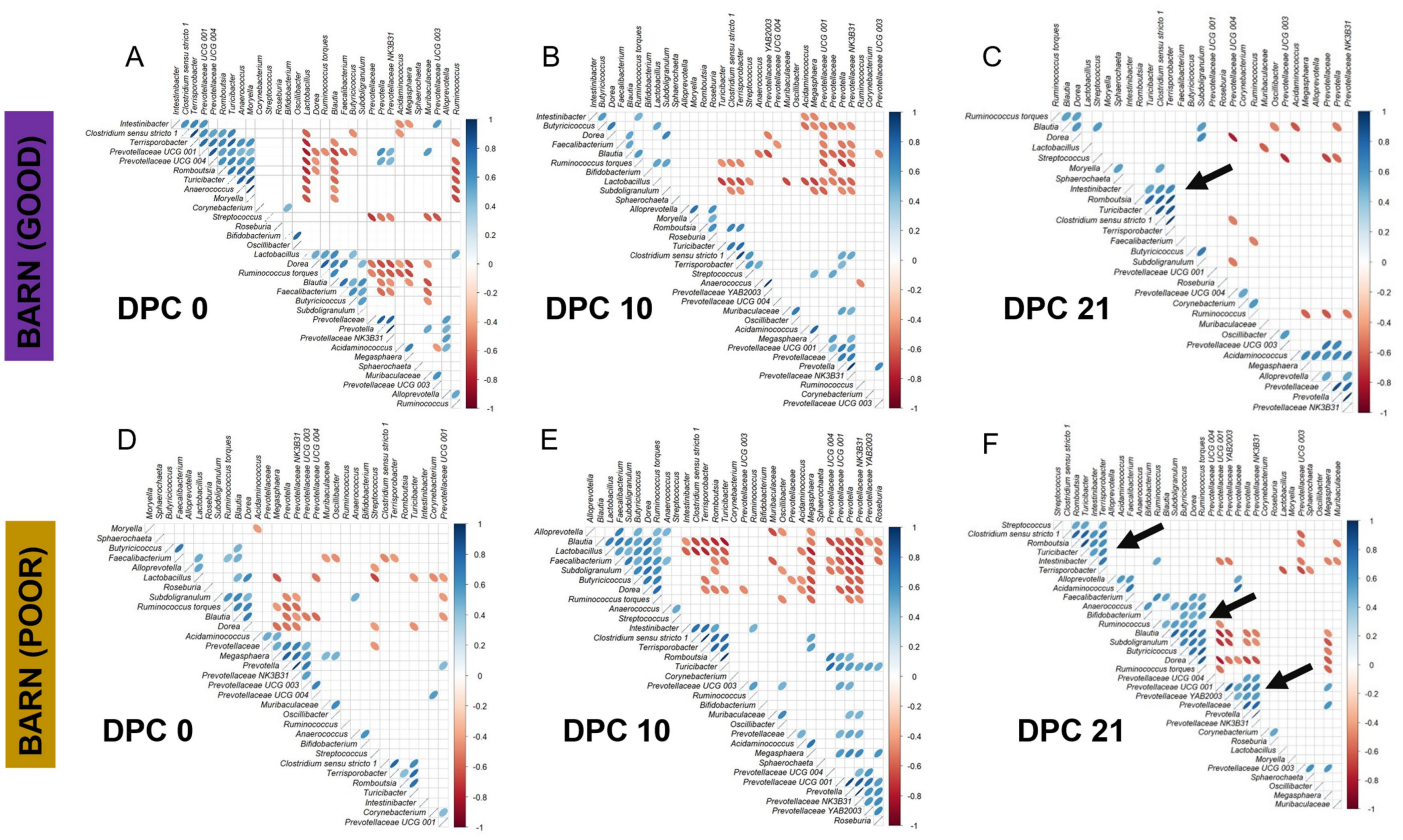
Correlation analysis of fecal microbiome taxa among pigs housed in barns under GOOD or POOR (*S.* Typhimurium inoculation + poor hygiene) sanitary conditions across DPC 0, 10, and 21. **(A–C)** Plots for the barn under GOOD sanitary conditions across DPC 0, 10, and 21, respectively. **(D–F)** Plots for the barn under POOR sanitary conditions across DPC 0, 10, and 21, respectively. In all plots, blue ellipses indicate a positive correlation, while red ellipses indicate a negative correlation. The thinner the ellipse, the stronger the correlation or anti-correlation between taxa. Pearson’s correlation was used at a *p*-value of < 0.05 significance level. Only pre-filtered keystone fecal microbiome taxa were included in the analysis (most differentiable between barns at DPC 21). The input data comprised rarefied proportions that were log2-transformed prior to calculating the correlation structure. Black arrows at DPC21 highlight important correlated clusters of taxa for either GOOD **(C)** or POOR (**F**) sanitary conditions.

A temporal clustering and correlation analysis using only the keystone differential fecal taxa confirmed a separation between communities based on barn SC status, most prominently at DPC 21 (Figures 7A–C), with no predictive signatures based on dietary modification (AA surplus) (Supplementary Figures 35–46). At DPC 21 (Figure 7C), there were two groups of taxa more likely to be enriched in the POOR (*S.* Typhimurium inoculation + poor hygiene) than the GOOD SC group: (1) a major sub-cluster comprised of *Clostridium sensu stricto 1*, *Intestinibacter*, *Romboutsia*, *Terrisporobacter*, and *Turicibacter* as previously captured by the temporal analysis of relative abundances (Figure 5 and Supplementary Figure 29); and (2) a secondary sub-cluster comprised of *Blautia*, *Butyricoccus*, *Dorea*, *Faecalibacterium*, *Ruminococcus torques*, and *Subdoligranulum*, with bimodal distribution-like patterns across animals in the POOR SC group. In accordance, these two sub-clusters demonstrated a correlated pattern within themselves, starting at DPC 10 and more clearly at DPC 21 for the POOR (*S.* Typhimurium inoculation + poor hygiene) SC group (Figures 8A–F). The correlation structure among these keystone fecal taxa did not appear to be predictably affected by dietary changes (Supplementary Figures 41–46).

## 4 Discussion

Pigs have a stable core fecal microbiome composition with the following genus being among the most predominant GI colonizers based on 16S rRNA compositional data: *Acidaminococcus, Blautia, Corynebacterium, Clostridium, Dorea, Eubacterium hallii, Intestinibacter, Lactobacillus, Megasphaera, Oscillospira, Prevotella, Roseburia, Ruminococcus, Solobacterium, Streptococcus, Subdoligranulum, Succinivibrio, Treponema,* and *Turicibacter* (16, 21, 24, 25). The GI microbiome contributes to pig health in a variety of ways, including, but not limited to, the regulation of peristalsis, nutrient digestion and absorption (e.g., fiber fermentation), biosynthesis of vitamins/SCFA, colonization resistance to enteric pathogens, immune development and maturation, and host metabolism in general (2, 17–22). As the GI microbiome composition can also affect inflammatory/immunological responses that are the basis for protective immunity, immunopathology, or disease tolerance (54–56), it is crucial to determine how sanitary housing conditions and infectious challenges with endemic pig pathogens such as *S.* Typhimurium can affect the microbiome community structure and animal performance and whether or not dietary changes commercially used to improve lean meat deposition (AA surplus) can mitigate dysbiosis and positively affect growth. Our previous study demonstrated that AA surplus could help in reducing the negative impact on the growth of pigs exposed to high sanitary pressure (continual exposure to fecal material from low-performance animals plus *S.* Typhimurium infection) (9, 11, 12).

*Salmonella* Typhimurium cannot only be pathogenic to growing pigs but is also a public health concern due to its zoonotic potential and multidrug resistance (57, 58). It is transmitted through the fecal-oral route across pen-mates and possibly between pens via shared water flow and/or by workers (e.g., contaminated boots) and interacts with the resident GI microbiome (22, 32, 34–36, 45, 59–65). Specifically, in the mouse model, *S.* Typhimurium, through the invasion of the ileal mucosa, can use its virulence apparatus to trigger malabsorption of dietary AA (e.g., lysine, ornithine, methionine, and ascorbic acid), shaping the downstream (ileal-cecal) nutrient landscape that influences its growth and the extent of the inflammatory process (pathology) (45, 66).

The imbalance in AA absorption in the gut is an emerging area of study in the context of *S.* Typhimurium pathogenesis and warrants further investigation. This pathogen is also known to elevate oxygen levels in the gut lumen as a result of epithelial inflammation, which leads to the depletion of SCFA-producing bacteria that typically inhibit *S.* Typhimurium growth. Therefore, it is expected that fiber fermenters (keystone members of the swine GI microbiome, specifically those in the large intestine) would be rapidly and transiently depleted post-*S.* Typhimurium infection can subsequently be restored at the genus level after the first 7–14 days post-infection, as *S.* Typhimurium population decreases in the GI tracts (ileal-cecal niche) and potentially goes silent (sub-clinical infection) in the mesenteric lymph nodes and/or liver (22, 36, 62). The previous data for this study corroborated the expected pattern for *S.* Typhimurium dynamics since most of the pigs shed it with a high load during the first-week post-exposure, with about 50% of the pigs shedding it up to 14 days post-exposure at an average of 1–2 log_10_ CFU/g of fecal material despite AA surplus in the POOR SC (*S.* Typhimurium inoculation + poor hygiene) group (9).

SCFA producers, such as *Prevotella*, *Clostridium sensu stricto 1*, *and Blautia,* among others, are known to be negatively correlated with either *S.* Typhimurium load and/or intestinal pathology in pigs (32, 67). *Prevotella* relative abundance has been previously shown to be a predictor of high shedders, suggesting that high initial *Prevotella* abundance may increase colonization resistance against *S.* Typhimurium (67). Nevertheless, little is known about *Prevotella* diversity in the swine gut at the species and strain levels. However, *P. copri* and *P. stercorea* are expected to be among the most dominant. It would be expected that a higher diversity of species and strains would be positively correlated with colonization resistance against both *S.* Typhimurium and *S*. Monophasic (29, 68, 69). In the mouse model, restoration of SCFA producers and/or SCFA can ameliorate disease (34, 35, 70). The microbiome’s ability to recover (resilience) is crucial for reestablishing SCFA and maintaining a stable community structure due to its pleiotropic effects on gut health and host metabolism (28). Notably, in this study, *Prevotella* and *Roseburia* were, on average, less abundant in the POOR (*S.* Typhimurium inoculation + poor hygiene) group compared to the GOOD SC group at DPC 10, potentially as an initial consequence of the *S.* Typhimurium infection. However, we lacked microbiome data for individual days during the first week, which would have provided a higher resolution of the community structure dynamics. Additionally, despite not reaching the statistical threshold of significance, perhaps due to the small sample size, *Prevotella* groups have a stronger positive correlation coefficient between fold changes from DPC 0 to 21 and average daily gain in this study for GOOD vs. POOR (*S.* Typhimurium inoculation + poor hygiene) SC, suggesting its potential positive impact on pig growth as a core member of the community post-weaning (16, 24, 25, 68).

Across all differentiable taxa between GOOD vs. POOR (*S.* Typhimurium inoculation + poor hygiene), SC, *Clostridium sensu stricto 1*, *Intestinibacter*, *Romboutsia*, *Terrisporobacter*, and *Turicibacter* were not only among the top hits individually but also formed a primary correlated network sub-cluster that explained most of the beta-diversity differences at DPC 21. Interestingly, for those five taxa, whether statistically significant or not, the mean relative abundance began to increase already at DPC 10 for the POOR (*S.* Typhimurium inoculation + poor hygiene) SC group. Most likely, these dynamics suggest a compensatory pattern, perhaps revealing the resilience of the resident microbiome for reestablishing GI health and homeostasis post the first 14 days of high exposure to *S.* Typhimurium and continual fecal exposure (poor hygiene). Interestingly, all five major taxa abovementioned tended on average to be lower in relative abundance for POOR AA vs. POOR CN (*S.* Typhimurium inoculation + poor hygiene), suggesting that the AA-supplemented diet did not promote the expansion of these taxa at DPC 21. These five taxa, along with the primary members of the secondary sub-cluster enriched in the POOR (*S.* Typhimurium inoculation + poor hygiene) SC group, comprise *Dorea*, *Ruminococcus*, and *Subdoligranulum, which* are capable of some SCFA production; their correlated pattern may be explained by cross-feeding or the utilization of several carbohydrates, proteins, and AAs (28, 71, 72). For instance, *Ruminococcus torques* can be a mucin degrader that generates oligosaccharides that other species can utilize (73).

Although our analytical approach was comprehensive in identifying the most differential keystone taxa, additional qPCR quantification at the genus level coupled with metagenomics would enhance our ability to assess temporal dynamics both at the genus and species level and the potential metabolic capabilities of the microbiota (74–76). Interestingly, *Turicibacter* appears to be a biomarker for autism spectrum disorder, therefore being associated with the gut–brain axis due to its potential to impact serotonin secretion in the intestine, which requires tryptophan for biosynthesis (77–79). As with *Prevotella copri,* whereby strain diversity differentially impacts polysaccharide utilization (80, 81), the genetic diversity of *Turicibacter* strains may differentially affect their ability to modify bile acids and host lipids (82). Two new studies have also demonstrated that *Turicibacter* is enriched in pigs under both social and heat stress, suggesting its potential role in host physiological adaptation, possibly through serotonin modulation (83, 84).

Despite its uniqueness, our study also presents some limitations, such as the use of 16S rRNA relative abundance data only, having had to rarefy the data at 5,000 counts as previously used by our group and others (12, 23), and the reproducibility of the sanitary conditions applied, which has been successfully used before, albeit with modifications (9, 11, 12). Therefore, we suggest the following opportunities to enhance the quality of this study and for future validating studies: (1) the need to optimize DNA extraction protocols to increase the DNA yield for 16S rRNA sequencing; (2) the need for qPCR for absolute quantification of target bacteria and temporal analysis based on appropriate power calculations for the phenotype in question; (3) the need to consider sampling different anatomic sites since biogeography can affect microbial dynamics in the *S.* Typhimurium pig infectious study (32); (4) the need to isolate the target taxon and conduct a whole-genome sequencing analysis to assess their genetic diversity and test for relevant phenotypes *in vitro*; (5) the need to consider the use of metagenomics on a subset of samples for further species characterization; (6) the need to consider how randomization might affect the compositional data analysis if a particular taxon is expected to change in relative abundance given the experimental conditions (blocking by enterotypes or specific taxon abundance prior to challenge or dietary changes); (7) the need to assess data distribution and apply a diverse array of methods to increase the confidence on the findings given the multidimensional sparse aspects of the data; (8) the need standardize how poor hygienic challenges will be done using fecal material from pig age-matching barns with low performance and/or other endemic sanitary issues (e.g., screening for predictable endemic pathogens given the geographical locations and historical data); and (9) the need for experimental ecological studies to assess the causative role of specific taxa for colonization resistance against *S.* Typhimurium and to improve pig performance under less optimal housing.

In conclusion, this study identified a collective list of bacterial genus taxa, including *Butyricoccus*, *Clostridium stricto sensu 1*, *Dorea*, *Intestinibacter*, *Romboutsia*, *Ruminococcus torques*, *Subdoligranulum*, *Terrisporobacter*, and *Turicibacter* (many of which are SCFA producers), that were enriched in the POOR SC group at the endpoint of this study, potentially suggesting an ecological recovery of the community post-*S.* Typhimurium infection and poor manure hygiene. That change occurred despite an AA surplus, suggesting that threonine, methionine, cysteine, and tryptophan did not directly affect these core members of the swine fecal microbiome, as indicated by 16S rRNA sequencing data. This suggests a potential role for SCFA producers in stabilizing the large intestine microbiome in the absence of consistently predictable positive associations with increased average daily gain by individual members.

## Data Availability

The datasets presented in this study can be found in online repositories. The names of the repository/repositories and the accession number(s) can be found at: https://www.ncbi.nlm.nih.gov/, PRJNA985664.
