# Supplementary material for: Temporal changes in fecal swine microbiome primarily reflect Salmonella Typhimurium challenge and poor sanitary housing conditions, even with functional amino acid supplementation
Source: Front Vet Sci. 2025 Jul 23;12:1597857. doi: 10.3389/fvets.2025.1597857 (PMC12327394; doi:10.3389/fvets.2025.1597857)
Supplement: Supplementary file 1 [file Data_Sheet_1.pdf]

SUPPLEMENTARY MATERIAL

A

TREATMENTS

Fecal samples	DPC 0	DPC 10	DPC 21
**GOOD CN**	10	10	8
**GOOD AA**	11	11	8
**POOR CN**	11	11	9
**POOR AA**	11	11	9

B

BARN (SC)

Fecal samples	DPC 0	DPC 10	DPC 21
**GOOD**	21	21	16
**POOR**	22	22	18

Supplementary Table 1 | Fecal microbiome sampling scheme. (**A**) Fecal microbiome sampling scheme across treatments (Diet – CN vs. AA) and housing sanitary status (Barn (SC) – GOOD vs. POOR). (**B**) Combined sampling scheme across barns (GOOD vs. POOR). DPC refers to Day Post-Challenge.

A

Barn SC	Taxa	Pearson	P-value
GOOD	*Acidaminococcus*	0.3	0.264
GOOD	*Alloprevotella*	0.38	0.149
GOOD	*Blautia*	0.017	0.95
GOOD	*Butyricicoccus*	0.17	0.521
GOOD	*Clostridium sensu stricto 1*	0.085	0.755
GOOD	*Corynebacterium*	-0.025	0.928
GOOD	*Dorea*	-0.23	0.383
GOOD	*Faecalibacterium*	-0.26	0.336
GOOD	*Intestinibacter*	0.043	0.875
GOOD	*Lactobacillus*	-0.46	0.0735
GOOD	*Megasphaera*	0.19	0.471
GOOD	*Moryella*	0.36	0.165
GOOD	*Muribaculaceae*	-0.09	0.739
GOOD	*Oscillibacter*	0.058	0.831
GOOD	*Prevotella*	0.46	0.0721
GOOD	*Prevotellaceae*	0.47	0.0635
GOOD	*Prevotellaceae NK3B31*	0.23	0.398
GOOD	*Prevotellaceae UCG 001*	0.6	**0.0137**
GOOD	*Prevotellaceae UCG 003*	-0.15	0.572
GOOD	*Prevotellaceae UCG 004*	0.15	0.591
GOOD	*Romboutsia*	0.12	0.665
GOOD	*Roseburia*	-0.032	0.905
GOOD	*Ruminococcus*	-0.14	0.595
GOOD	*Ruminococcus torques*	-0.23	0.393
GOOD	*Sphaerochaeta*	0.53	**0.0364**
GOOD	*Streptococcus*	0.25	0.341
GOOD	*Subdoligranulum*	0.39	0.134
GOOD	*Terrisporobacter*	0.21	0.435
GOOD	*Turicibacter*	-0.031	0.908

B

Barn SC	Taxa	Pearson	P-value
POOR	*Acidaminococcus*	-0.092	0.717
POOR	*Alloprevotella*	0.28	0.259
POOR	*Anaerococcus*	-0.17	0.487
POOR	*Bifidobacterium*	0.25	0.315
POOR	*Blautia*	-0.033	0.897
POOR	*Butyricicoccus*	-0.28	0.266
POOR	*Clostridium sensu stricto 1*	0.17	0.49
POOR	*Corynebacterium*	0.097	0.703
POOR	*Dorea*	-0.065	0.798
POOR	*Faecalibacterium*	0.25	0.324
POOR	*Intestinibacter*	-0.23	0.36
POOR	*Lactobacillus*	0.21	0.393
POOR	*Megasphaera*	-0.12	0.644
POOR	*Moryella*	-0.18	0.474
POOR	*Muribaculaceae*	-0.016	0.95
POOR	*Oscillibacter*	-0.51	**0.03**
POOR	*Prevotella*	0.1	0.686
POOR	*Prevotellaceae*	0.28	0.252
POOR	*Prevotellaceae NK3B31*	0.091	0.721
POOR	*Prevotellaceae UCG 001*	0.41	0.0918
POOR	*Prevotellaceae UCG 003*	0.0057	0.982
POOR	*Prevotellaceae UCG 004*	-0.1	0.685
POOR	*Prevotellaceae YAB2003*	0.063	0.804
POOR	*Romboutsia*	-0.42	0.0863
POOR	*Roseburia*	0.14	0.566
POOR	*Ruminococcus*	-0.031	0.901
POOR	*Ruminococcus torques*	-0.21	0.404
POOR	*Sphaerochaeta*	0.36	0.137
POOR	*Streptococcus*	-0.37	0.133
POOR	*Subdoligranulum*	-0.25	0.319
POOR	*Terrisporobacter*	0.17	0.508
POOR	*Turicibacter*	-0.49	**0.0402**

Supplementary Table 2 | Pearson's correlation coefficient and P-value (with significant level < 0.05) for the correlation between fold-change in taxon abundance from DPC 0 to DPC 21 vs. average daily gain for the same period, comparing GOOD (**A**) vs. POOR (**B**) barn SC groups.

A

Treatments	Taxa	Pearson	P-value
GOOD CN	*Acidaminococcus*	0.28	0.505
GOOD CN	*Alloprevotella*	0.47	0.245
GOOD CN	*Blautia*	0.14	0.747
GOOD CN	*Butyricicoccus*	0.18	0.671
GOOD CN	*Clostridium sensu stricto 1*	0.61	0.105
GOOD CN	*Corynebacterium*	0.14	0.747
GOOD CN	*Dorea*	-0.084	0.843
GOOD CN	*Faecalibacterium*	-0.064	0.88
GOOD CN	*Intestinibacter*	0.42	0.305
GOOD CN	*Lactobacillus*	-0.3	0.478
GOOD CN	*Megasphaera*	0.52	0.185
GOOD CN	*Moryella*	0.86	**0.00623**
GOOD CN	*Muribaculaceae*	-0.17	0.688
GOOD CN	*Oscillibacter*	-0.51	0.195
GOOD CN	*Prevotella*	0.62	0.101
GOOD CN	*Prevotellaceae*	0.55	0.156
GOOD CN	*Prevotellaceae NK3B31*	0.55	0.157
GOOD CN	*Prevotellaceae UCG 001*	0.58	0.133
GOOD CN	*Prevotellaceae UCG 003*	-0.21	0.614
GOOD CN	*Prevotellaceae UCG 004*	-0.46	0.254
GOOD CN	*Romboutsia*	0.61	0.109
GOOD CN	*Roseburia*	0.0096	0.982
GOOD CN	*Ruminococcus*	-0.055	0.897
GOOD CN	*Ruminococcus torques*	0.078	0.855
GOOD CN	*Sphaerochaeta*	0.73	**0.0404**
GOOD CN	*Streptococcus*	-0.22	0.598
GOOD CN	*Subdoligranulum*	0.48	0.231
GOOD CN	*Terrisporobacter*	0.2	0.642
GOOD CN	*Turicibacter*	0.34	0.405

B

Treatments	Taxa	Pearson	P-value
GOOD AA	*Acidaminococcus*	0.31	0.451
GOOD AA	*Alloprevotella*	0.36	0.387
GOOD AA	*Blautia*	-0.18	0.675
GOOD AA	*Butyricicoccus*	-0.00013	1
GOOD AA	*Clostridium sensu stricto 1*	-0.076	0.858
GOOD AA	*Corynebacterium*	-0.14	0.745
GOOD AA	*Dorea*	-0.51	0.198
GOOD AA	*Faecalibacterium*	-0.59	0.123
GOOD AA	*Intestinibacter*	-0.33	0.428
GOOD AA	*Lactobacillus*	-0.76	**0.0284**
GOOD AA	*Megasphaera*	-0.057	0.894
GOOD AA	*Muribaculaceae*	0.22	0.608
GOOD AA	*Oscillibacter*	0.4	0.33
GOOD AA	*Prevotella*	0.42	0.306
GOOD AA	*Prevotellaceae*	0.42	0.302
GOOD AA	*Prevotellaceae NK3B31*	-0.027	0.949
GOOD AA	*Prevotellaceae UCG 001*	0.61	0.112
GOOD AA	*Prevotellaceae UCG 003*	0.044	0.918
GOOD AA	*Prevotellaceae UCG 004*	0.68	0.0637
GOOD AA	*Romboutsia*	-0.25	0.547
GOOD AA	*Roseburia*	-0.046	0.914
GOOD AA	*Ruminococcus*	-0.019	0.965
GOOD AA	*Ruminococcus torques*	-0.58	0.133
GOOD AA	*Sphaerochaeta*	0.42	0.3
GOOD AA	*Streptococcus*	0.35	0.398
GOOD AA	*Subdoligranulum*	0.2	0.635
GOOD AA	*Terrisporobacter*	0.17	0.69
GOOD AA	*Turicibacter*	-0.36	0.379

Supplementary Table 3 | Pearson's correlation coefficient and P-value (with significant level < 0.05) for the correlation between fold-change in taxon abundance from DPC 0 to DPC 21 vs. average daily gain for the same period, comparing GOOD CN (**A**) vs. GOOD AA (**B**) groups.

A

Treatments	Taxa	Pearson	P-value
POOR CN	*Acidaminococcus*	0.13	0.748
POOR CN	*Alloprevotella*	-0.4	0.284
POOR CN	*Anaerococcus*	-0.37	0.329
POOR CN	*Bifidobacterium*	0.26	0.503
POOR CN	*Blautia*	-0.049	0.9
POOR CN	*Butyricicoccus*	-0.5	0.167
POOR CN	*Clostridium sensu stricto 1*	0.49	0.176
POOR CN	*Corynebacterium*	0.21	0.596
POOR CN	*Dorea*	-0.46	0.211
POOR CN	*Faecalibacterium*	0.36	0.348
POOR CN	*Intestinibacter*	-0.53	0.142
POOR CN	*Lactobacillus*	0.28	0.467
POOR CN	*Megasphaera*	-0.27	0.484
POOR CN	*Moryella*	-0.14	0.72
POOR CN	*Muribaculaceae*	-0.33	0.392
POOR CN	*Oscillibacter*	-0.44	0.239
POOR CN	*Prevotella*	-0.049	0.901
POOR CN	*Prevotellaceae*	-0.09	0.818
POOR CN	*Prevotellaceae NK3B31*	0.2	0.611
POOR CN	*Prevotellaceae UCG 001*	0.42	0.266
POOR CN	*Prevotellaceae UCG 003*	-0.45	0.228
POOR CN	*Prevotellaceae UCG 004*	0.14	0.718
POOR CN	*Romboutsia*	-0.64	0.0626
POOR CN	*Roseburia*	0.11	0.783
POOR CN	*Ruminococcus*	-0.054	0.89
POOR CN	*Ruminococcus torques*	-0.27	0.486
POOR CN	*Sphaerochaeta*	0.29	0.448
POOR CN	*Streptococcus*	-0.47	0.197
POOR CN	*Subdoligranulum*	-0.39	0.304
POOR CN	*Terrisporobacter*	0.42	0.261
POOR CN	*Turicibacter*	-0.61	0.0806

B

Treatments	Taxa	Pearson	P-value
POOR AA	*Acidaminococcus*	-0.36	0.34
POOR AA	*Alloprevotella*	0.66	0.0548
POOR AA	*Anaerococcus*	0.31	0.421
POOR AA	*Bifidobacterium*	0.32	0.404
POOR AA	*Blautia*	0.026	0.946
POOR AA	*Butyricicoccus*	-0.093	0.811
POOR AA	*Clostridium sensu stricto 1*	0.073	0.852
POOR AA	*Corynebacterium*	0.32	0.408
POOR AA	*Dorea*	0.11	0.769
POOR AA	*Faecalibacterium*	0.028	0.943
POOR AA	*Intestinibacter*	0.19	0.627
POOR AA	*Lactobacillus*	0.016	0.967
POOR AA	*Megasphaera*	0.3	0.427
POOR AA	*Moryella*	-0.16	0.69
POOR AA	*Muribaculaceae*	0.14	0.719
POOR AA	*Oscillibacter*	-0.58	0.102
POOR AA	*Prevotella*	0.071	0.857
POOR AA	*Prevotellaceae*	0.41	0.273
POOR AA	*Prevotellaceae NK3B31*	-0.08	0.837
POOR AA	*Prevotellaceae UCG 001*	0.6	0.0873
POOR AA	*Prevotellaceae UCG 003*	0.68	**0.0459**
POOR AA	*Prevotellaceae UCG 004*	-0.52	0.154
POOR AA	*Prevotellaceae YAB2003*	0.019	0.962
POOR AA	*Romboutsia*	0.00055	0.999
POOR AA	*Roseburia*	0.26	0.506
POOR AA	*Ruminococcus*	0.019	0.96
POOR AA	*Ruminococcus torques*	0.067	0.864
POOR AA	*Sphaerochaeta*	0.39	0.3
POOR AA	*Streptococcus*	-0.21	0.588
POOR AA	*Subdoligranulum*	0.15	0.7
POOR AA	*Terrisporobacter*	-0.043	0.913
POOR AA	*Turicibacter*	-0.16	0.675

Supplementary Table 4 | Pearson's correlation coefficient and P-value (with significant level < 0.05) for the correlation between fold-change in taxon abundance from DPC 0 to DPC 21 vs. average daily gain for the same period, comparing POOR CN (**A**) vs. POOR AA (**B**) groups.

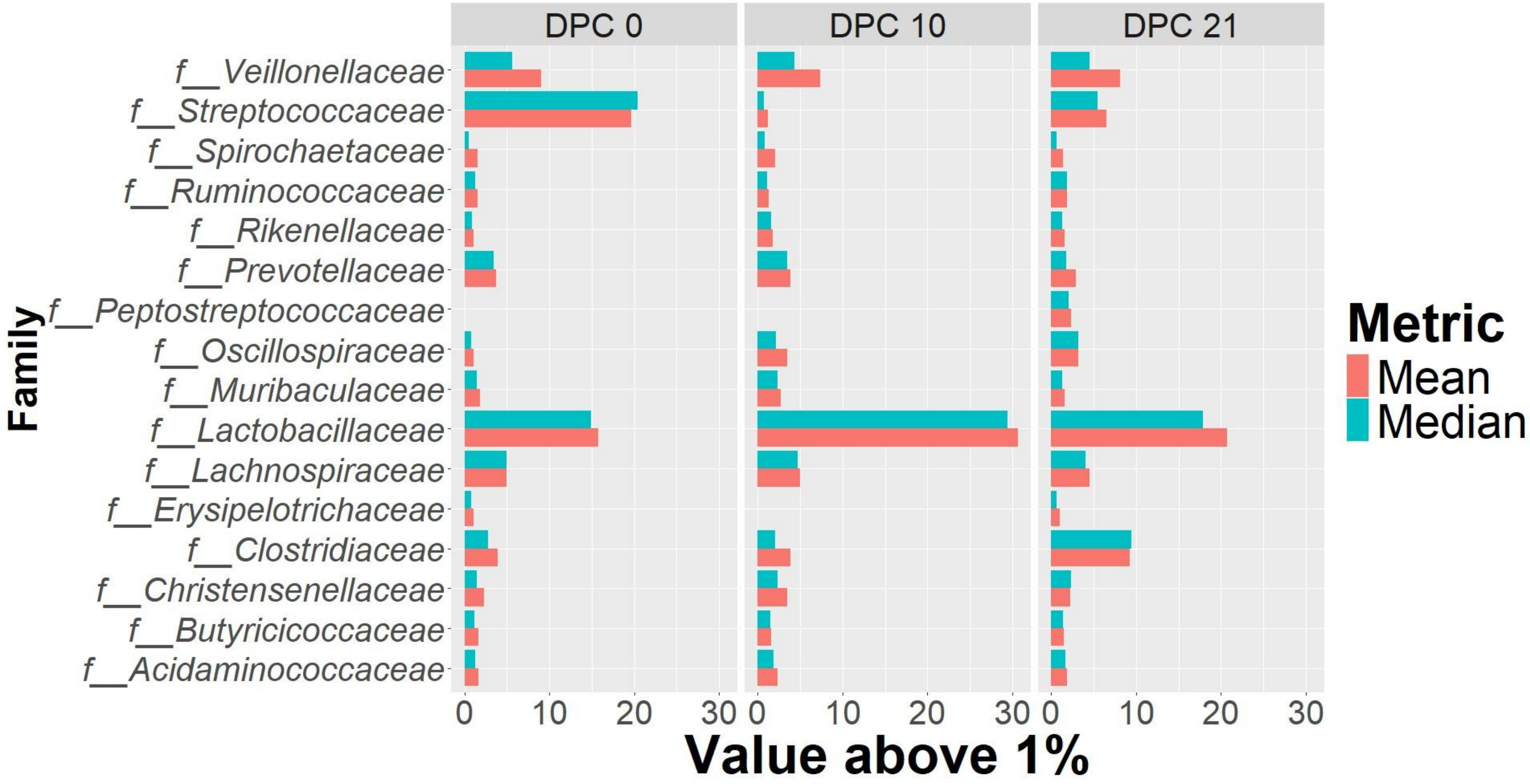

Supplementary Figure 1 | Mean- and median-based proportion of taxa, at the Family level, above 1% cut-off across DPC 0, 10, and 21 regardless of treatment.

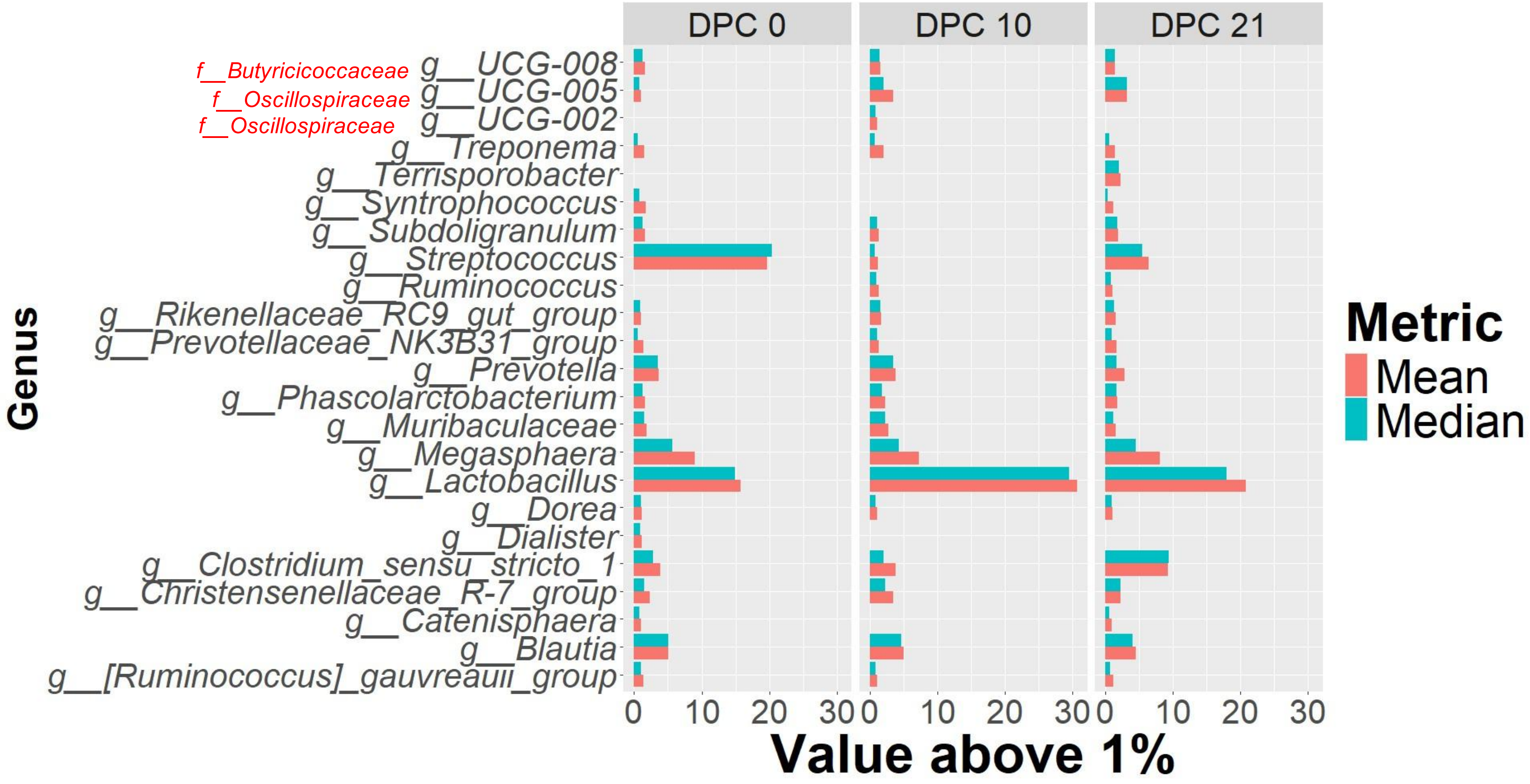

Supplementary Figure 2 | Mean- and median-based proportion of taxa, at the Genus level, above 1% cut-off across DPC 0, 10, and 21 regardless of treatment.

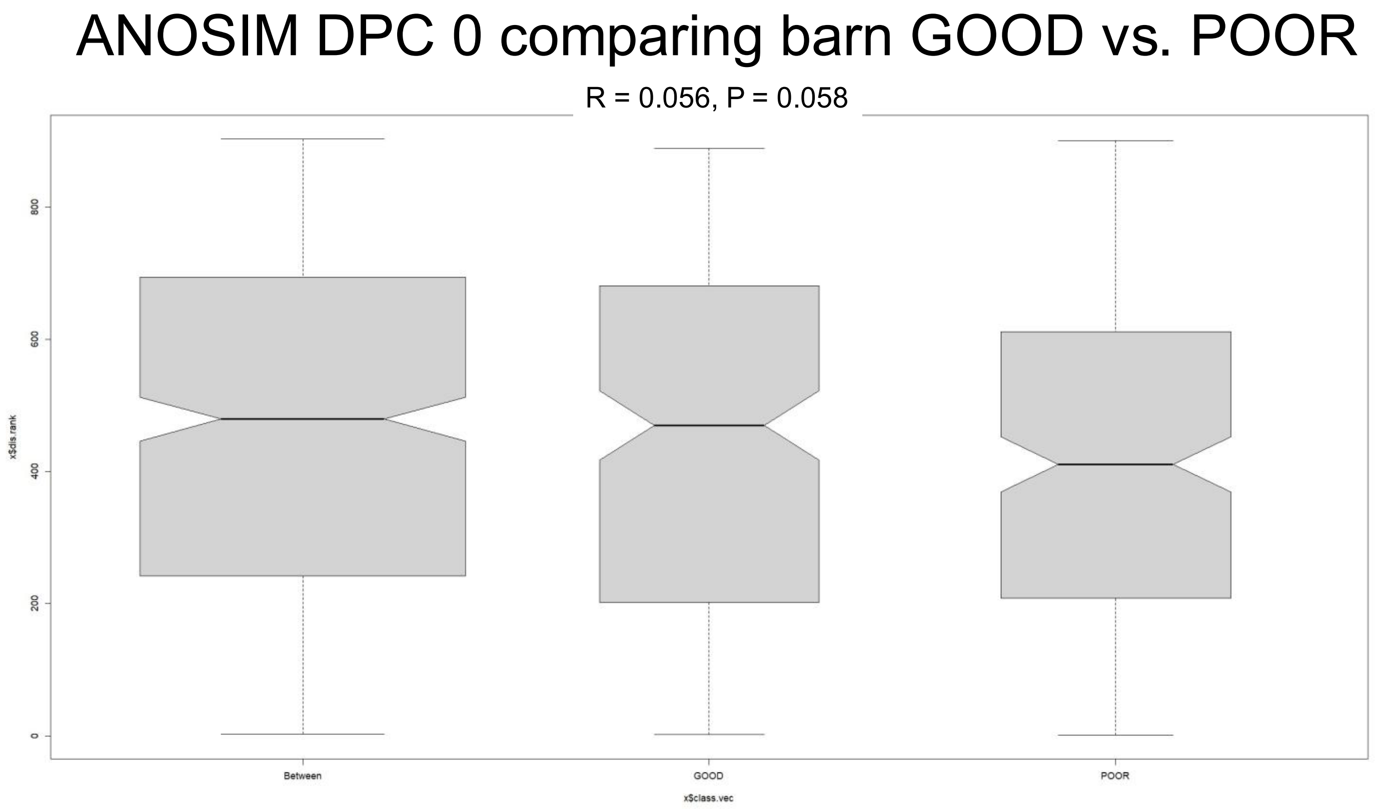

Supplementary Figure 3 | ANOSIM results for DPC 0 across barn (SC) GOOD vs. POOR. On the y-axis is the rank of dissimilarity entry. On the x-axis are all individual treatments (groups) and the between groups entry. ANOSIM was calculated using a Bray-Curtis distance matrix with rarefied counts by taxon as input data.

ANOSIM DPC 10 comparing barn GOOD vs. POOR

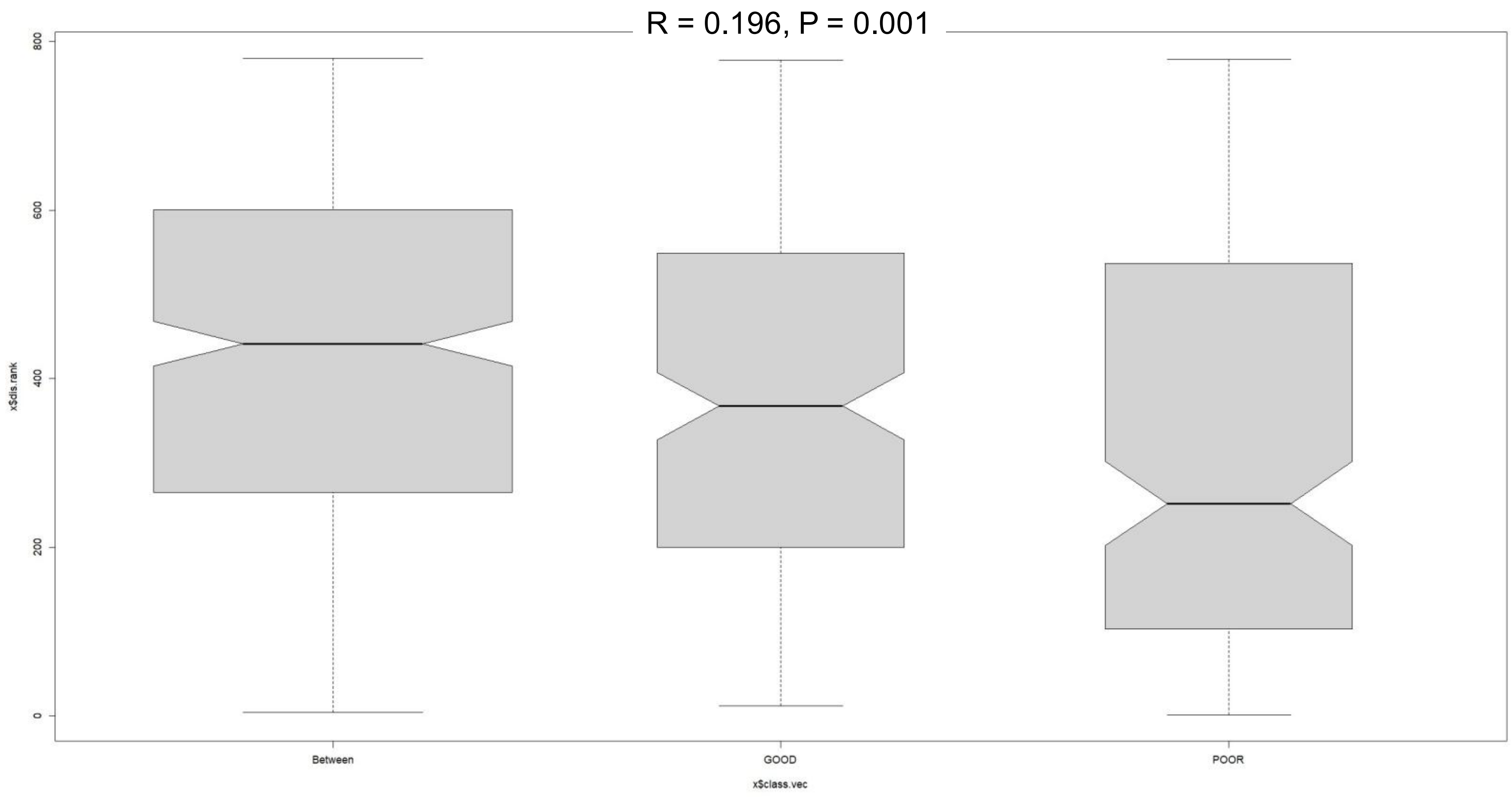

Supplementary Figure 4 | ANOSIM results for DPC 10 across barn (SC) GOOD vs. POOR. On the y-axis is the rank of dissimilarity entry. On the x-axis are all individual treatments (groups) and the between groups entry. ANOSIM was calculated using a Bray-Curtis distance matrix with rarefied counts by taxon as input data.

ANOSIM DPC 21 comparing barn GOOD vs. POOR

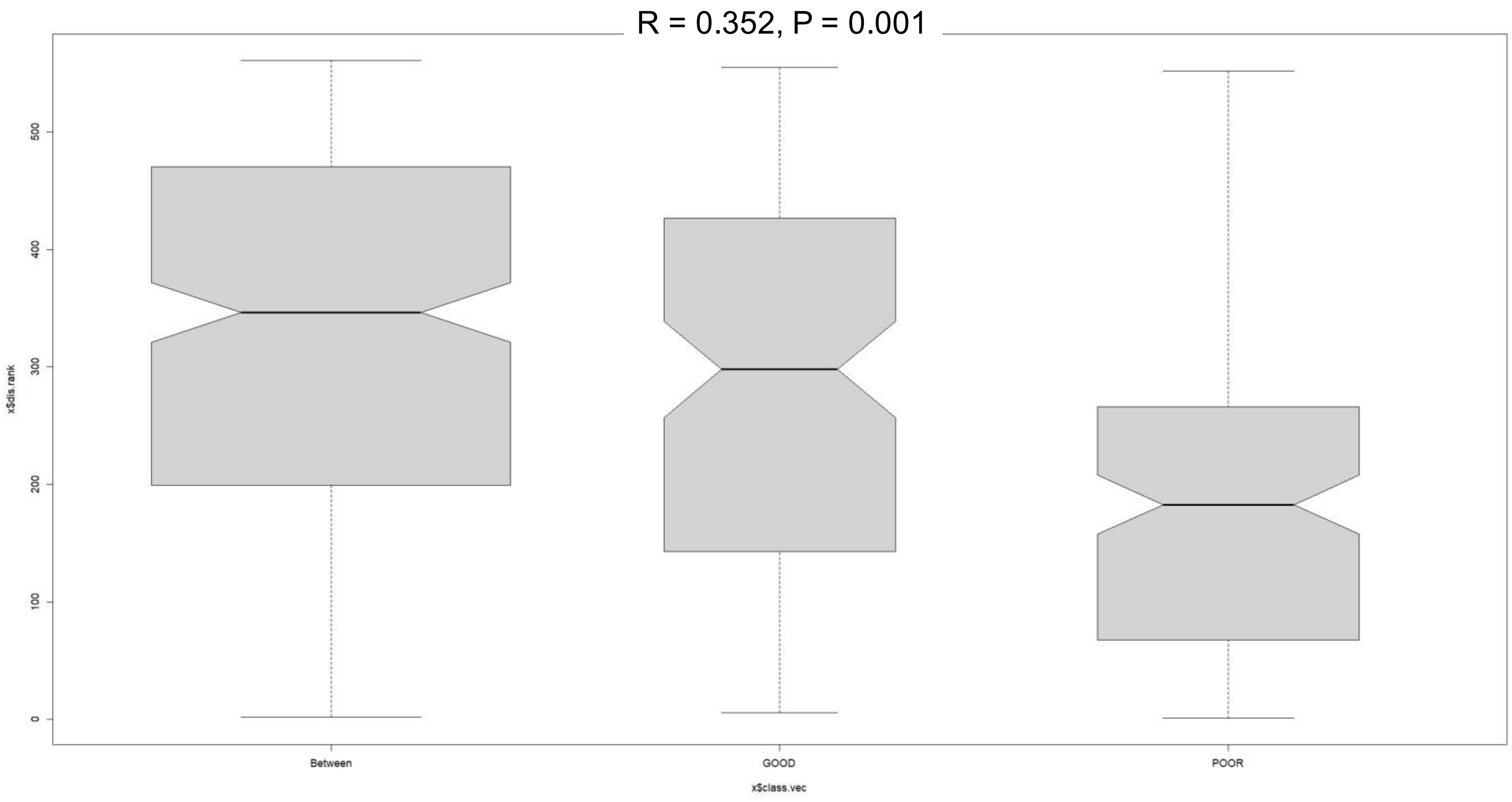

Supplementary Figure 5 | ANOSIM results for DPC 21 across barn (SC) GOOD vs. POOR. On the y-axis is the rank of dissimilarity entry. On the x-axis are all individual treatments (groups) and the between groups entry. ANOSIM was calculated using a Bray-Curtis distance matrix with rarefied counts by taxon as input data.

ANOSIM DPC 0 comparing all treatments

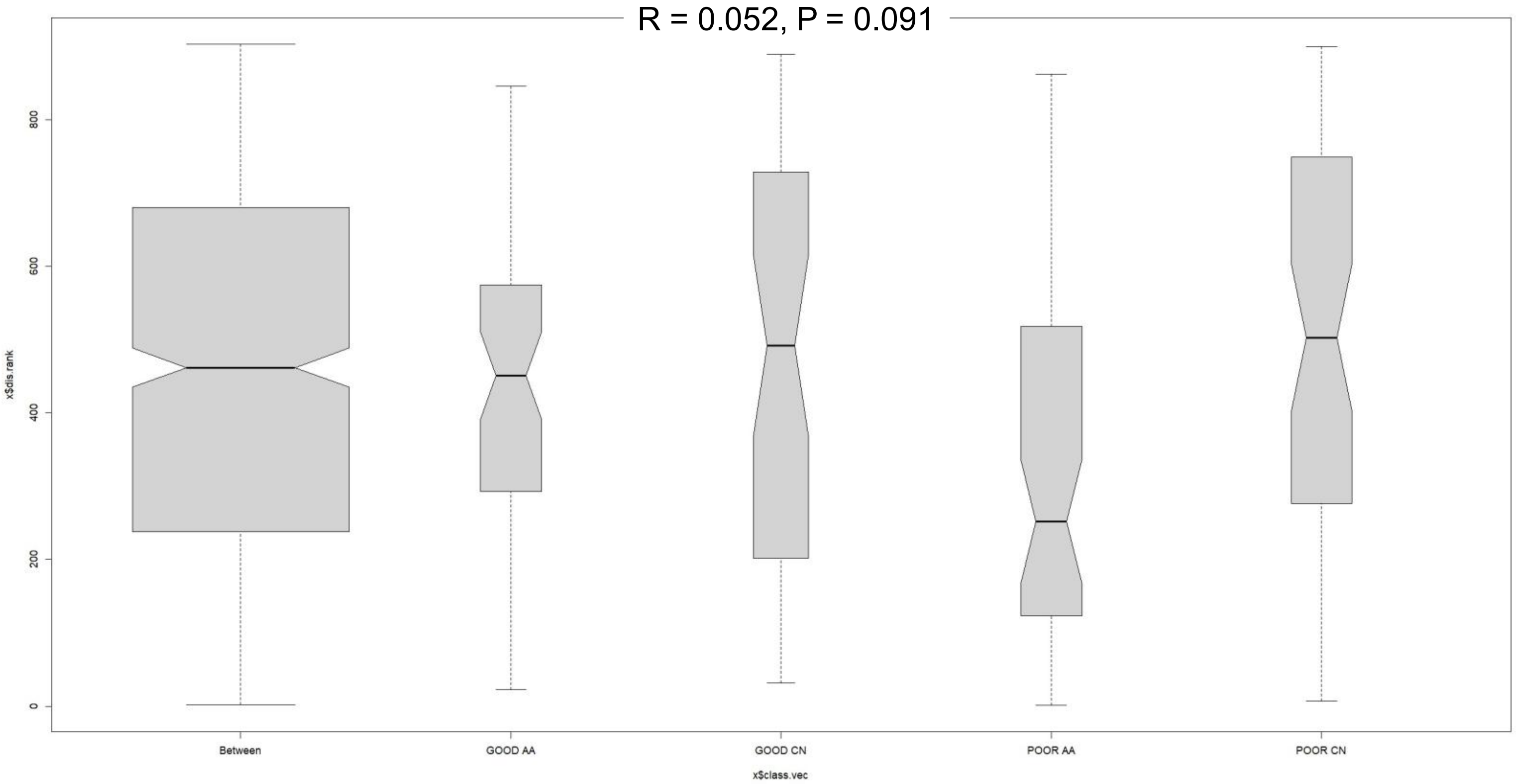

Supplementary Figure 6 | ANOSIM results for DPC 0 across all treatment combining both barn (SC) (GOOD vs. POOR) and diet (CN vs. AA). On the y-axis is the rank of dissimilarity entry. On the x-axis are all individual treatments (groups) and the between groups entry. ANOSIM was calculated using a Bray-Curtis distance matrix with rarefied counts by taxon as input data.

ANOSIM DPC 10 comparing all treatments

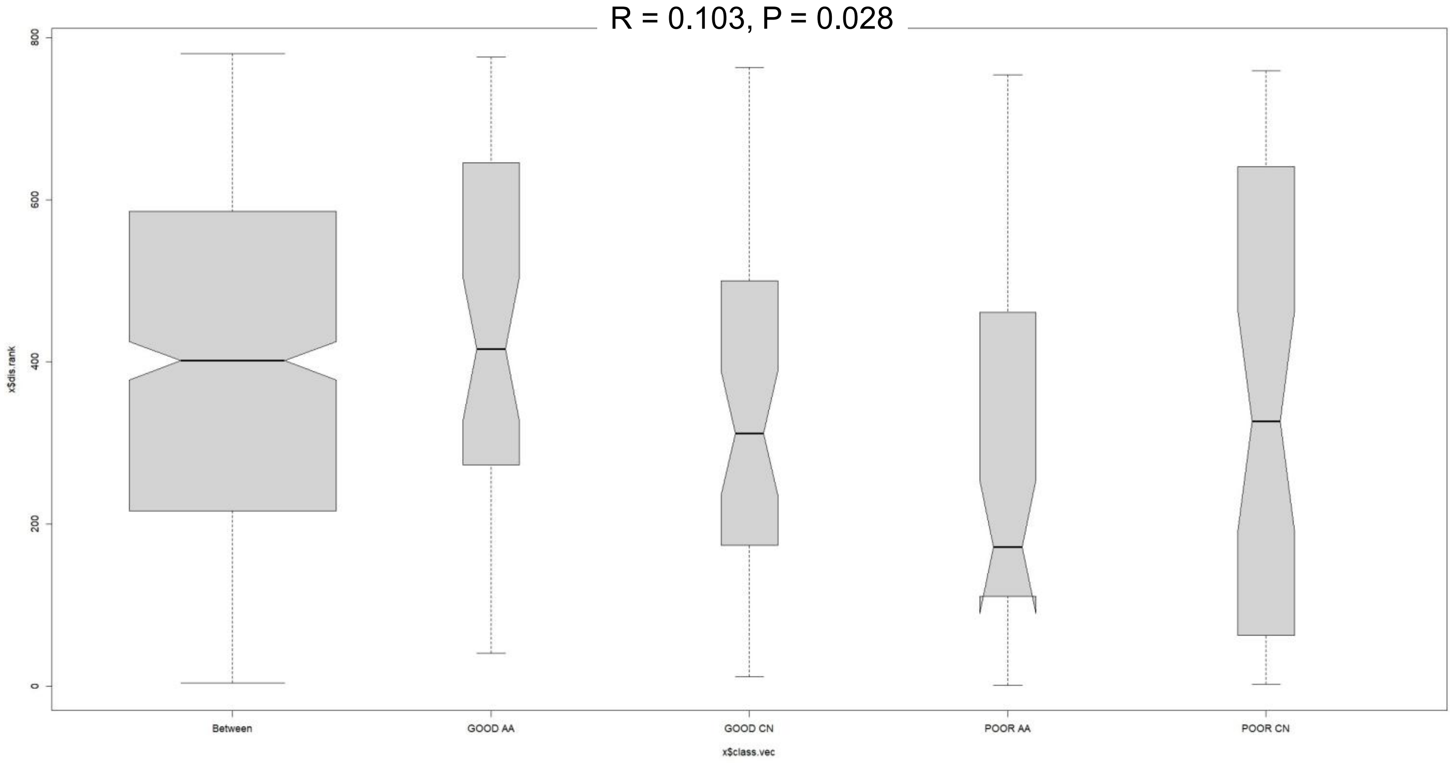

Supplementary Figure 7 | ANOSIM results for DPC 10 across all treatment combining both barn (SC) (GOOD vs. POOR) and diet (CN vs. AA). On the y-axis is the rank of dissimilarity entry. On the x-axis are all individual treatments (groups) and the between groups entry. ANOSIM was calculated using a Bray-Curtis distance matrix with rarefied counts by taxon as input data.

ANOSIM DPC 21 comparing all treatments

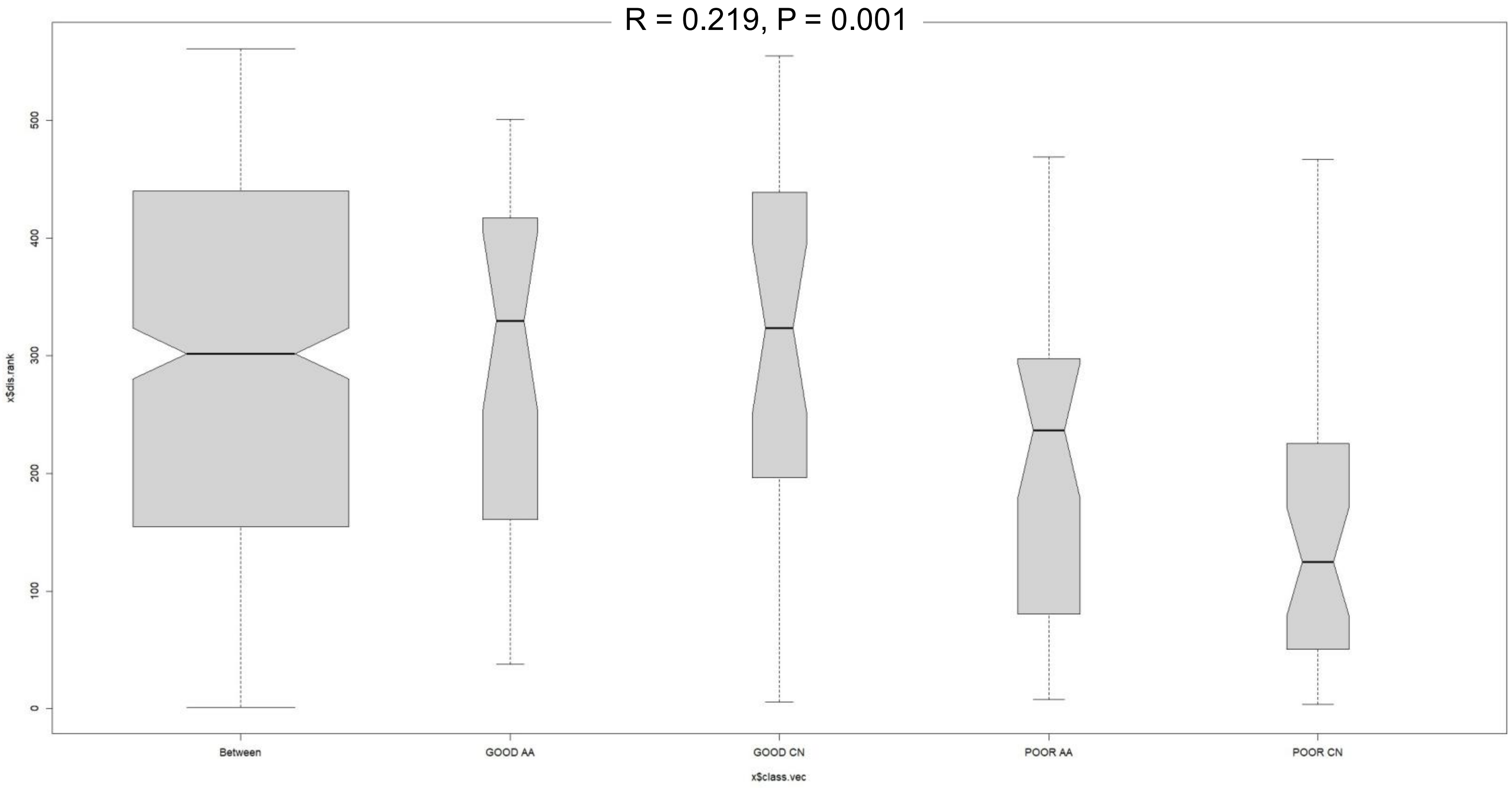

Supplementary Figure 8 | ANOSIM results for DPC 21 across all treatment combining both barn (SC) (GOOD vs. POOR) and diet (CN vs. AA). On the y-axis is the rank of dissimilarity entry. On the x-axis are all individual treatments (groups) and the between groups entry. ANOSIM was calculated using a Bray-Curtis distance matrix with rarefied counts by taxon as input data.

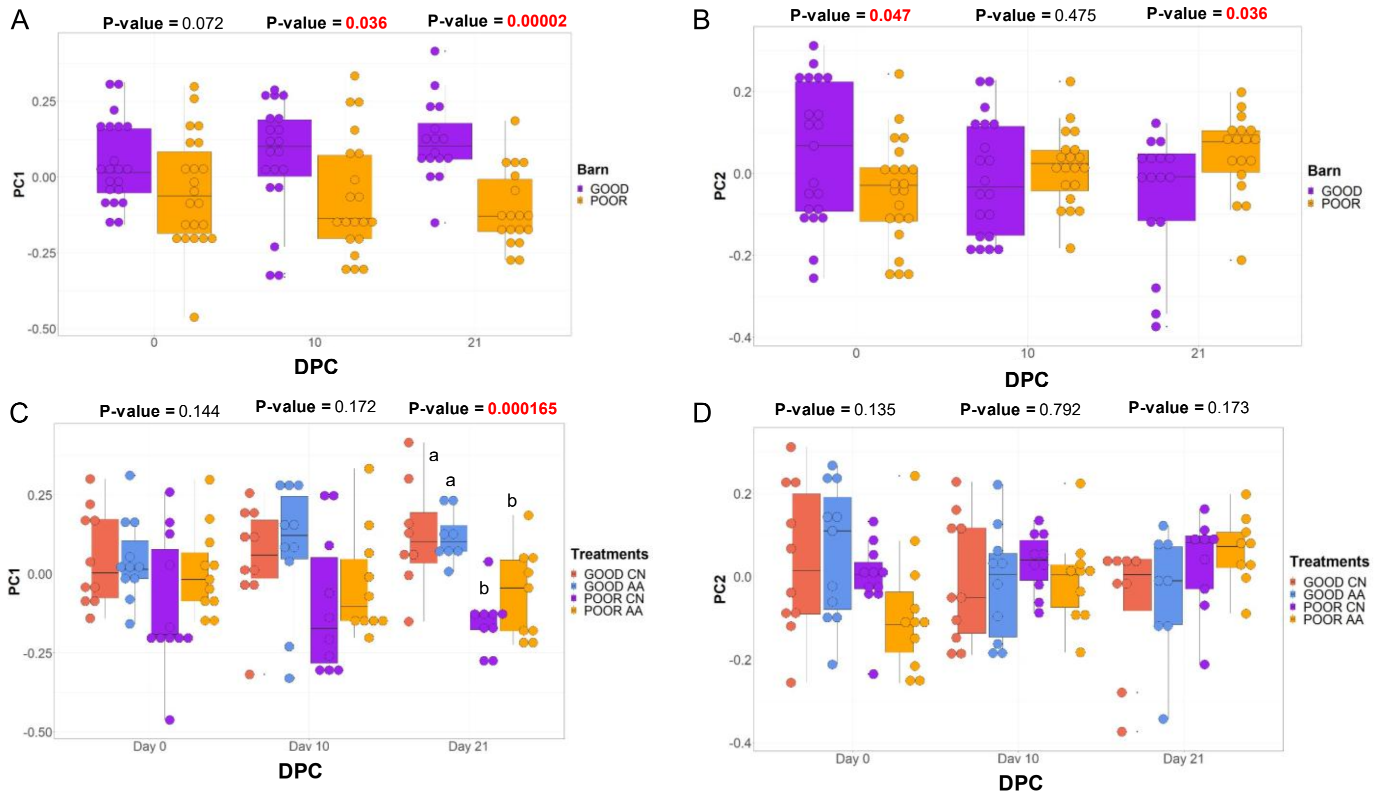

Supplementary Figure 9 | Community structure volatility analysis presented by principal coordinate 1 or 2 (PC1 or PC2) plots by day post-challenged (DPC, 0, 10, and 21) across sanitary conditions (GOOD vs. POOR) or treatments (diet × barn SC). Each PC was shown across diet and/or barns SC at DPC 0, 10, and 21 presenting the results of beta-diversity of the fecal microbiome composition using the Bray Curtis's distance matrix (input data -> rarified counts). The higher the variability of community structure across DPC, the higher the volatility. (**A-B**) Barn SC effect was considered when P < 0.05 based on a two-sided T-test (significant P-values highlighted in red). (**C-D**) Treatment effect was considered significant only when P < 0.05 using a one-way ANOVA analysis (significant P-values highlighted in red), followed by pairwise comparisons done using a pairwise two-sided T-test. Different superscript letters indicate significant differences between treatments.

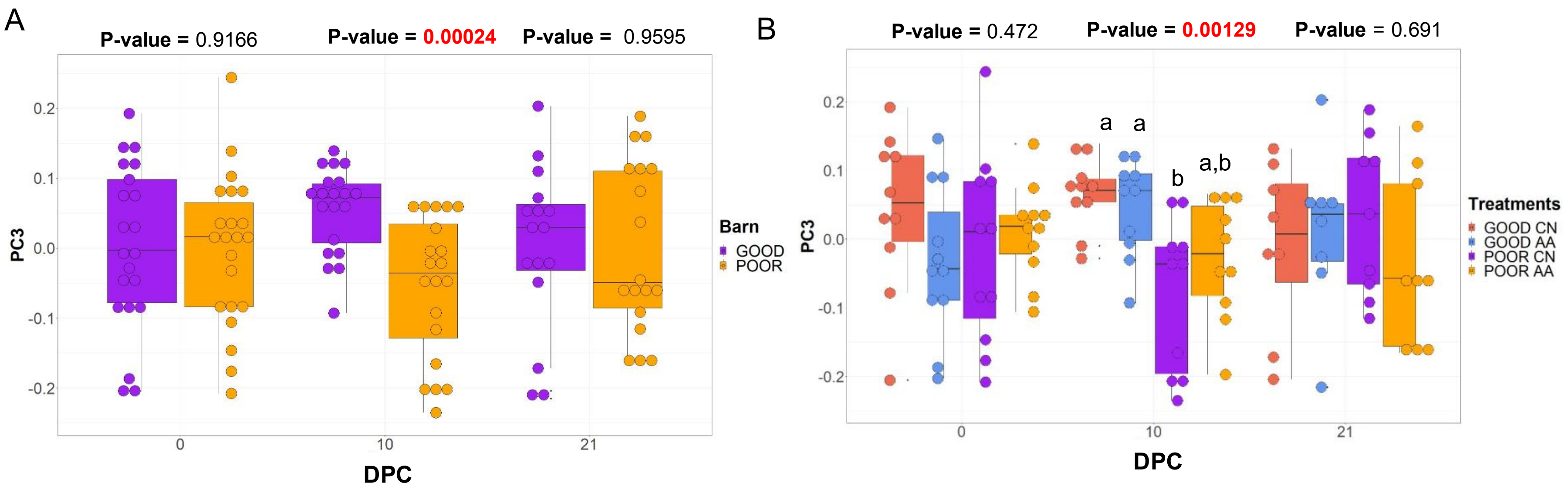

Supplementary Figure 10 | Community structure volatility analysis presented by principal coordinate 3 (PC3) plots by day post-challenged (DPC, 0, 10, and 21) across sanitary conditions (GOOD vs. POOR) or treatments (diet × barn SC). PC3 was shown across diet and/or barns SC at DPC 0, 10, and 21 presenting the results of beta-diversity of the fecal microbiome composition using the Bray Curtis's distance matrix (input data -> rarified counts). The higher the variability of community structure across DPC, the higher the volatility. (**A**) Barn SC effect was considered when P < 0.05 based on a two-sided T-test (significant P-values highlighted in red). (**B**) Treatment effect was considered significant only when P < 0.05 using a one-way ANOVA analysis (significant P-values highlighted in red), followed by pairwise comparisons done using a pairwise two-sided T-test. Different superscript letters indicate significant differences between treatments.

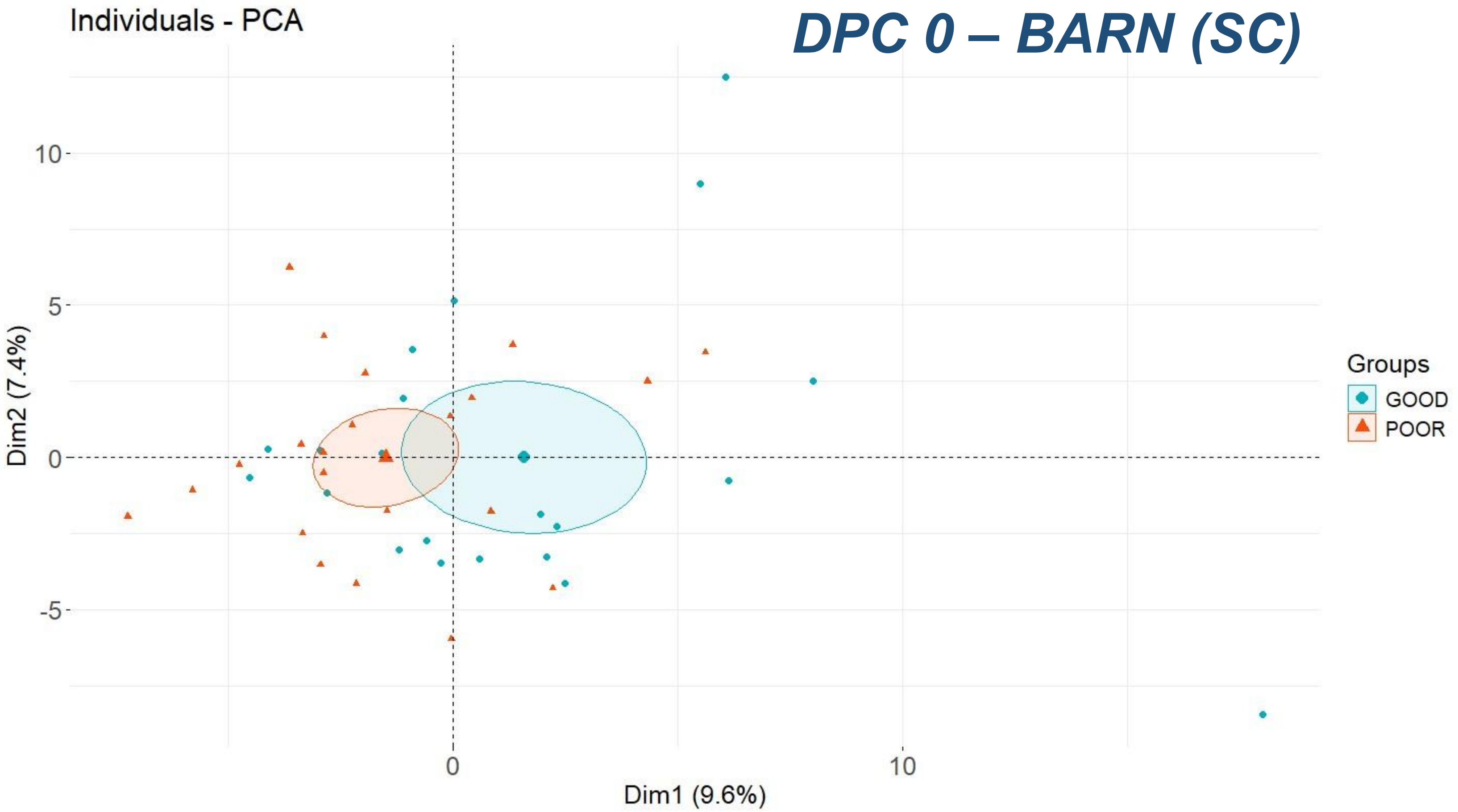

Supplementary Figure 11 | Principal component analysis across barn (SC) with different sanitary conditions (GOOD vs. POOR) at DPC 0. Principal component analysis was done using log_2 transformed proportion across bacterial taxa using rarified counts as input data. Two principal components were selected based on scree (elbow) plot (considering percentage of explained variance across dimensions).

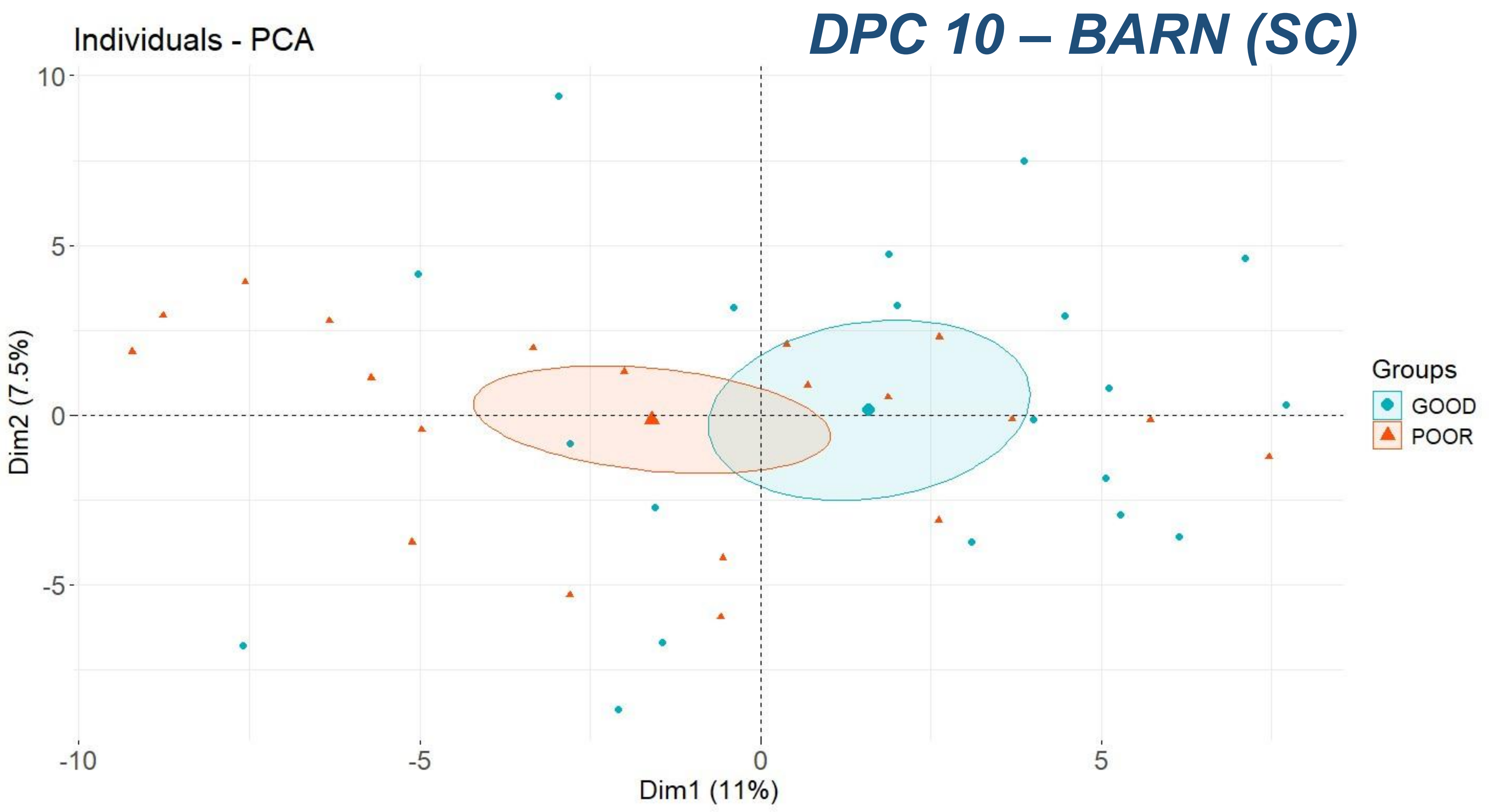

Supplementary Figure 12 | Principal component analysis across barn (SC) with different sanitary conditions (GOOD vs. POOR) at DPC 10. Principal component analysis was done using log_2 transformed proportion across bacterial taxa using rarified counts as input data. Two principal components were selected based on scree (elbow) plot (considering percentage of explained variance across dimensions).

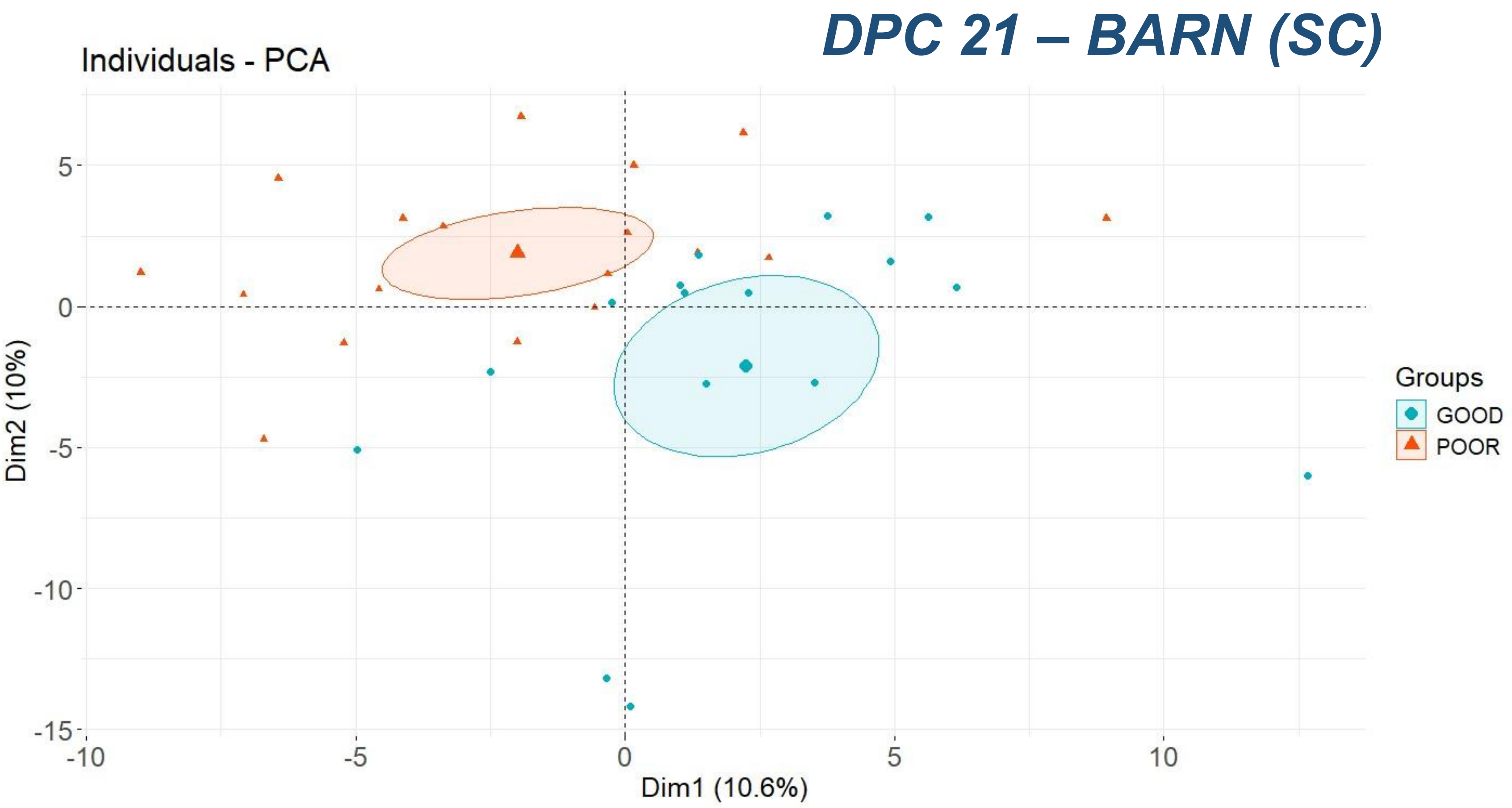

Supplementary Figure 13 | Principal component analysis across barn (SC) with different sanitary conditions (GOOD vs. POOR) at DPC 21. Principal component analysis was done using $\log_2$ transformed proportion across bacterial taxa using rarified counts as input data. Two principal components were selected based on scree (elbow) plot (considering percentage of explained variance across dimensions).

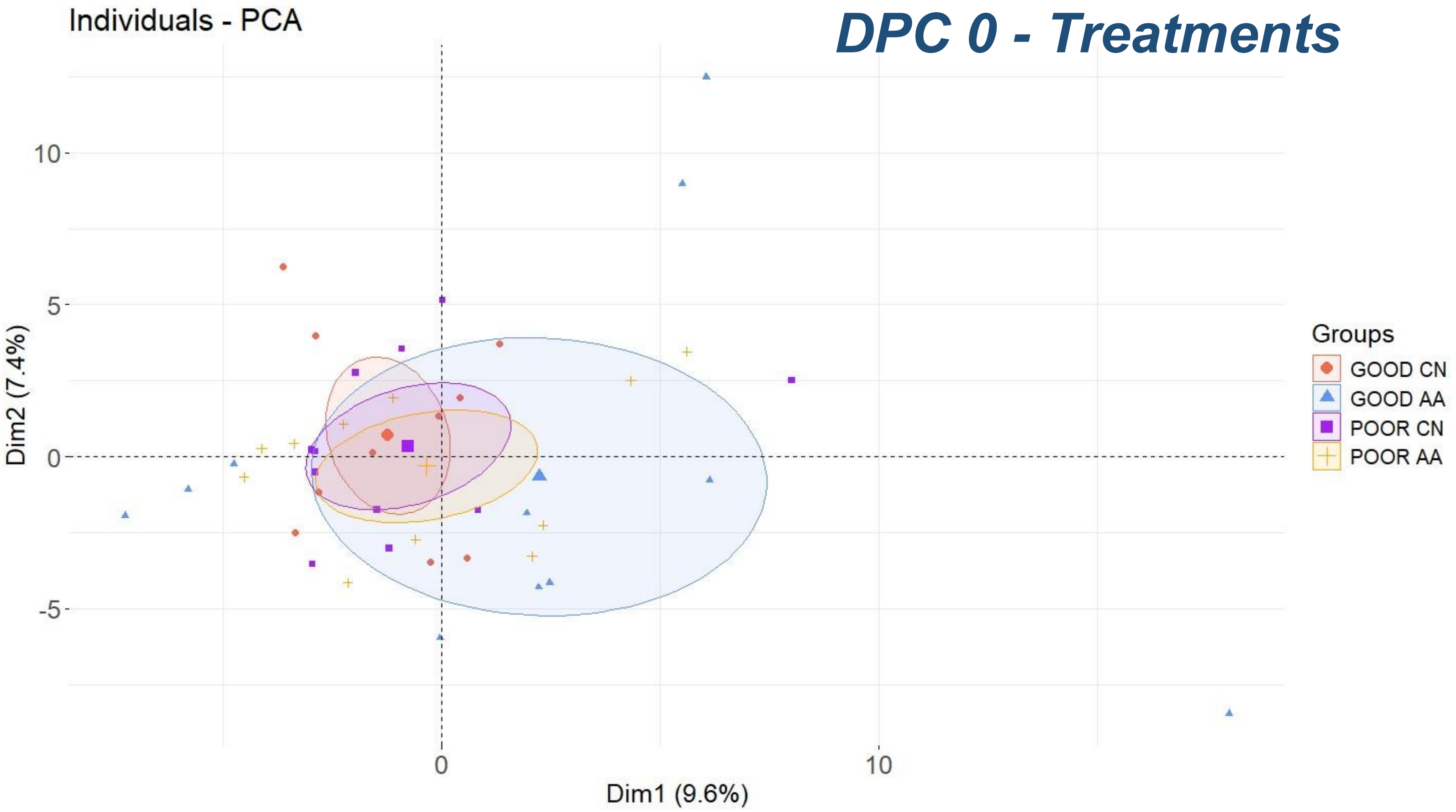

Supplementary Figure 14 | Principal component analysis across all treatments: combination of both barn (SC) with different sanitary conditions (GOOD vs. POOR) and diet (CON for conventional and AA for supplemented with functional amino acids) at DPC 0. Principal component analysis was done using log_2 transformed proportion across bacterial taxa using rarified counts as input data. Two principal components were selected based on scree (elbow) plot (considering percentage of explained variance across dimensions).

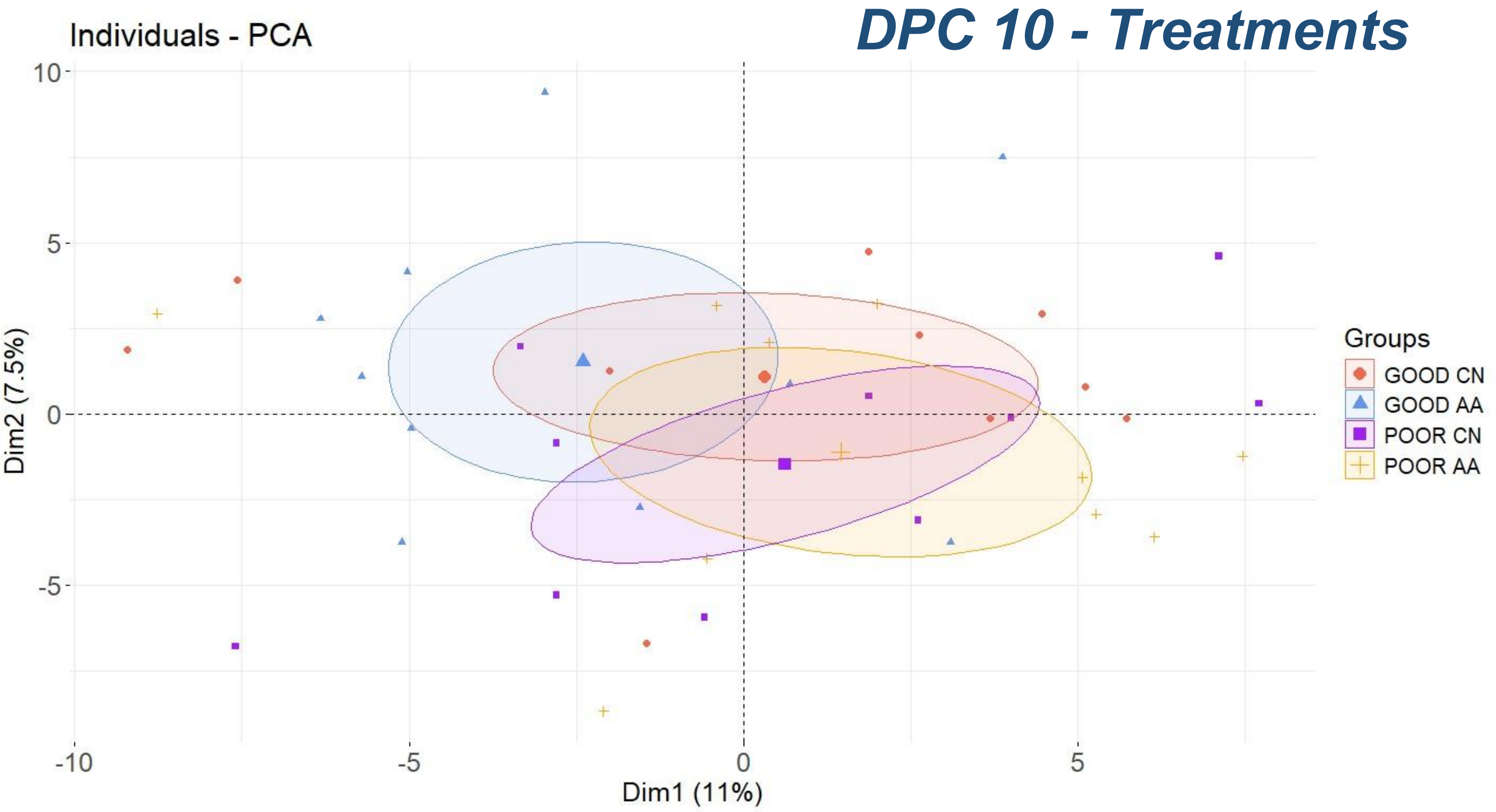

Supplementary Figure 15 | Principal component analysis across all treatments: combination of both barn (SC) with different sanitary conditions (GOOD vs. POOR) and diet (CON for conventional and AA for supplemented with functional amino acids) at DPC 10. Principal component analysis was done using log_2 transformed proportion across bacterial taxa using rarified counts as input data. Two principal components were selected based on scree (elbow) plot (considering percentage of explained variance across dimensions).

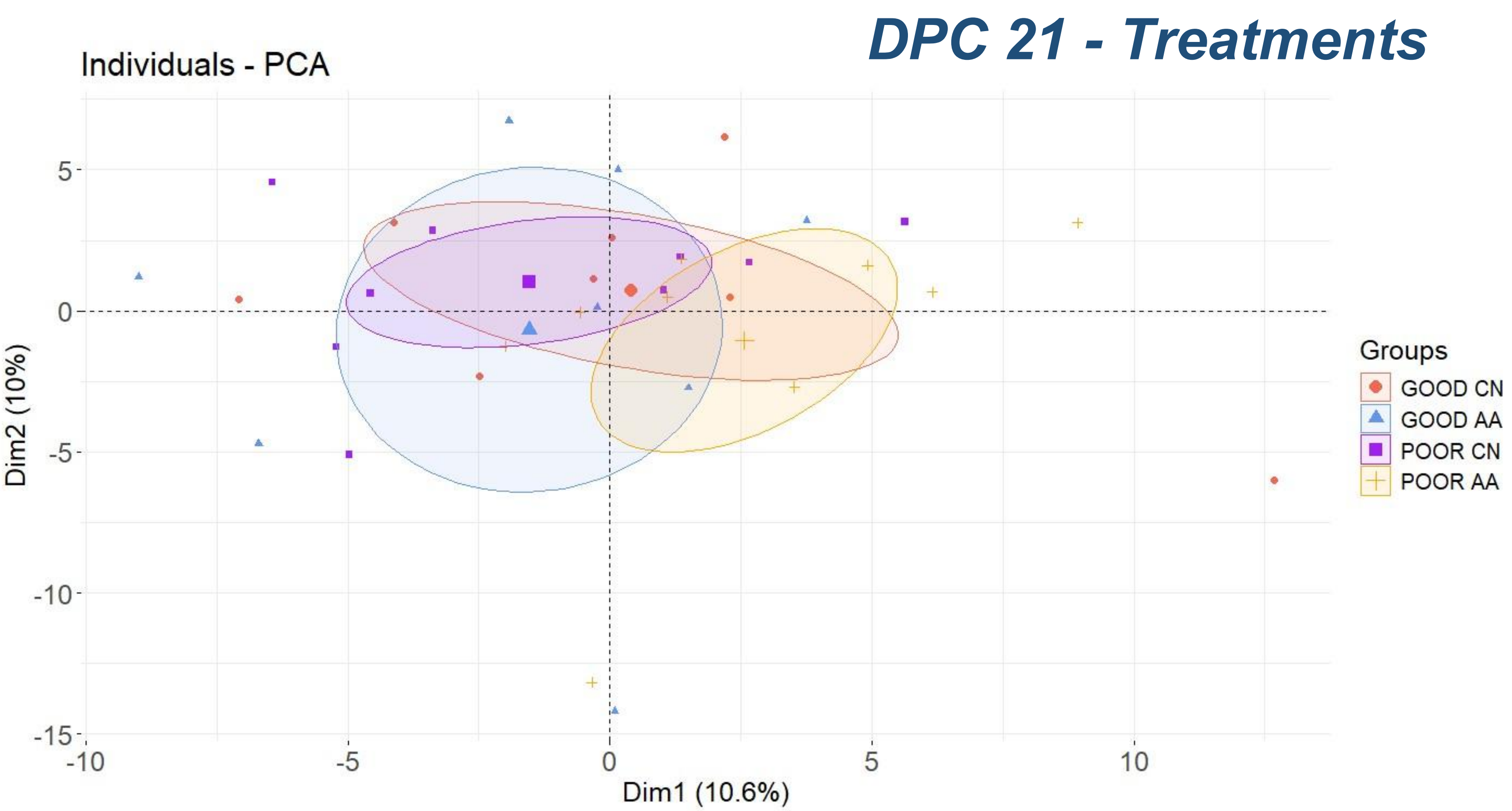

Supplementary Figure 16 | Principal component analysis across all treatments: combination of both barn (SC) with different sanitary conditions (GOOD vs. POOR) and diet (CON for conventional and AA for supplemented with functional amino acids) at DPC 0. Principal component analysis was done using $\log_2$ transformed proportion across bacterial taxa using rarified counts as input data. Two principal components were selected based on scree (elbow) plot (considering percentage of explained variance across dimensions).

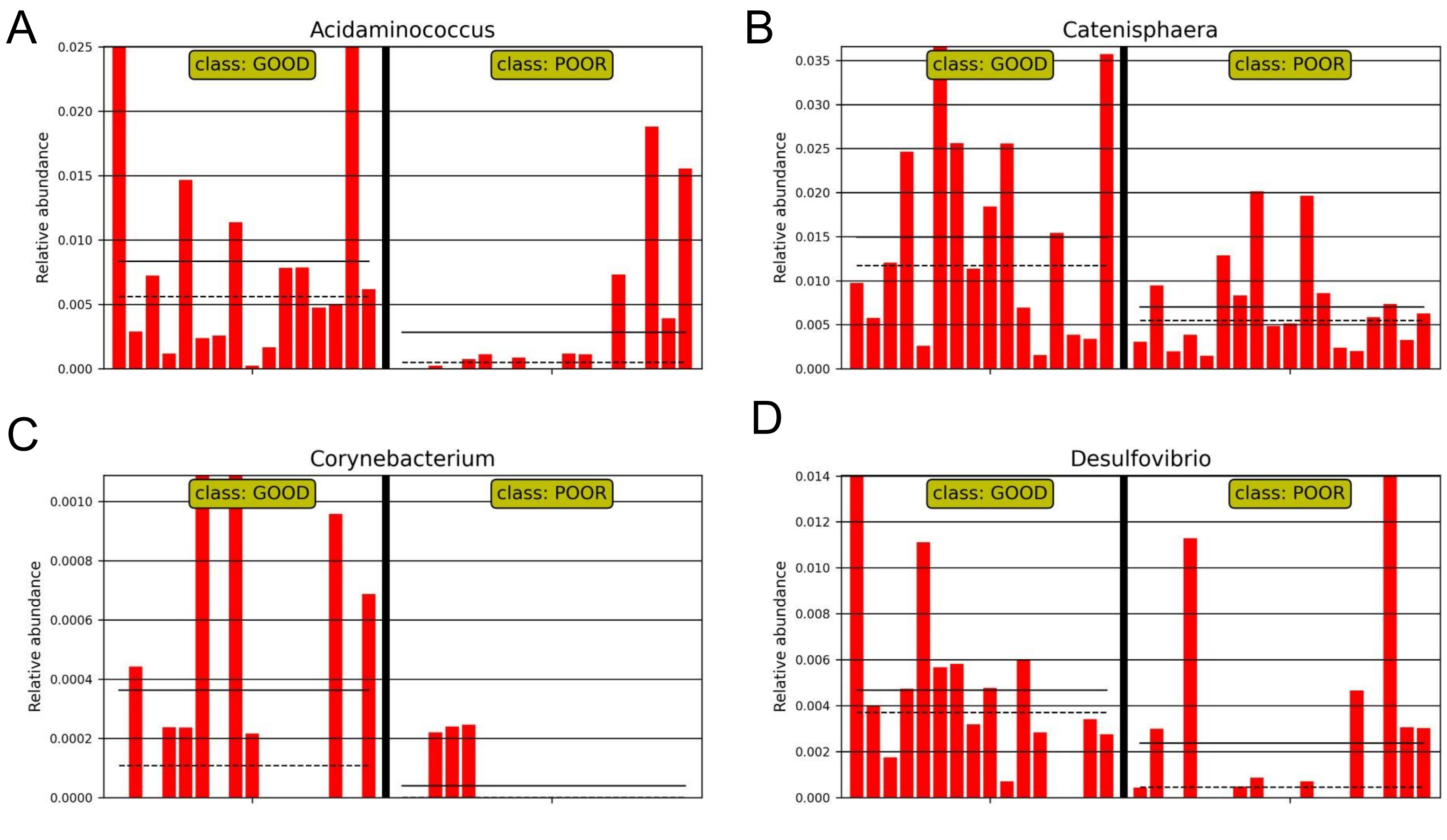

Supplementary Figure 17 | Individual animal relative frequency-based distribution of taxa across barn (SC) conditions (GOOD vs. POOR) at DPC 21. Relative abundance of taxa (**A** - *Acidaminococcus*, **B** - *Catenisphaera*, **C** - *Corynebacterium*, and **D** - *Desulfovibrio*) as part of the LEfSe analysis to identify major differentiating taxa between GOOD vs. POOR at the endpoint (DPC 21).

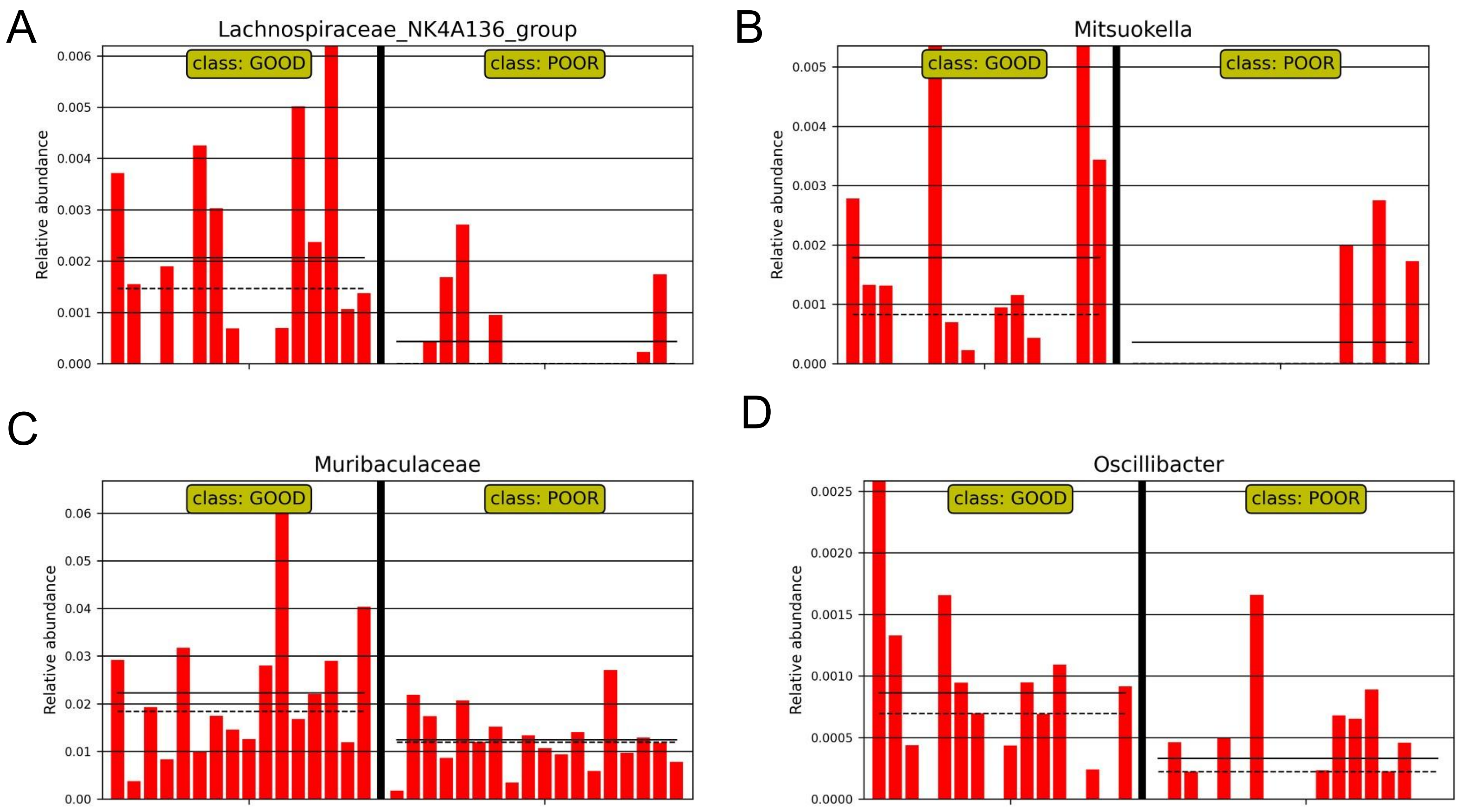

Supplementary Figure 18 | Individual animal relative frequency-based distribution of taxa across barn (SC) conditions (GOOD vs. POOR) at DPC 21. Relative abundance of taxa (**A** – *Lachnospiraceae NK4A136 group*, **B** - *Mitsuokella*, **C** - *Muribaculaceae*, and **D** - *Oscillibacter*) as part of the LEfSe analysis to identify major differentiating taxa between GOOD vs. POOR at the endpoint (DPC 21).

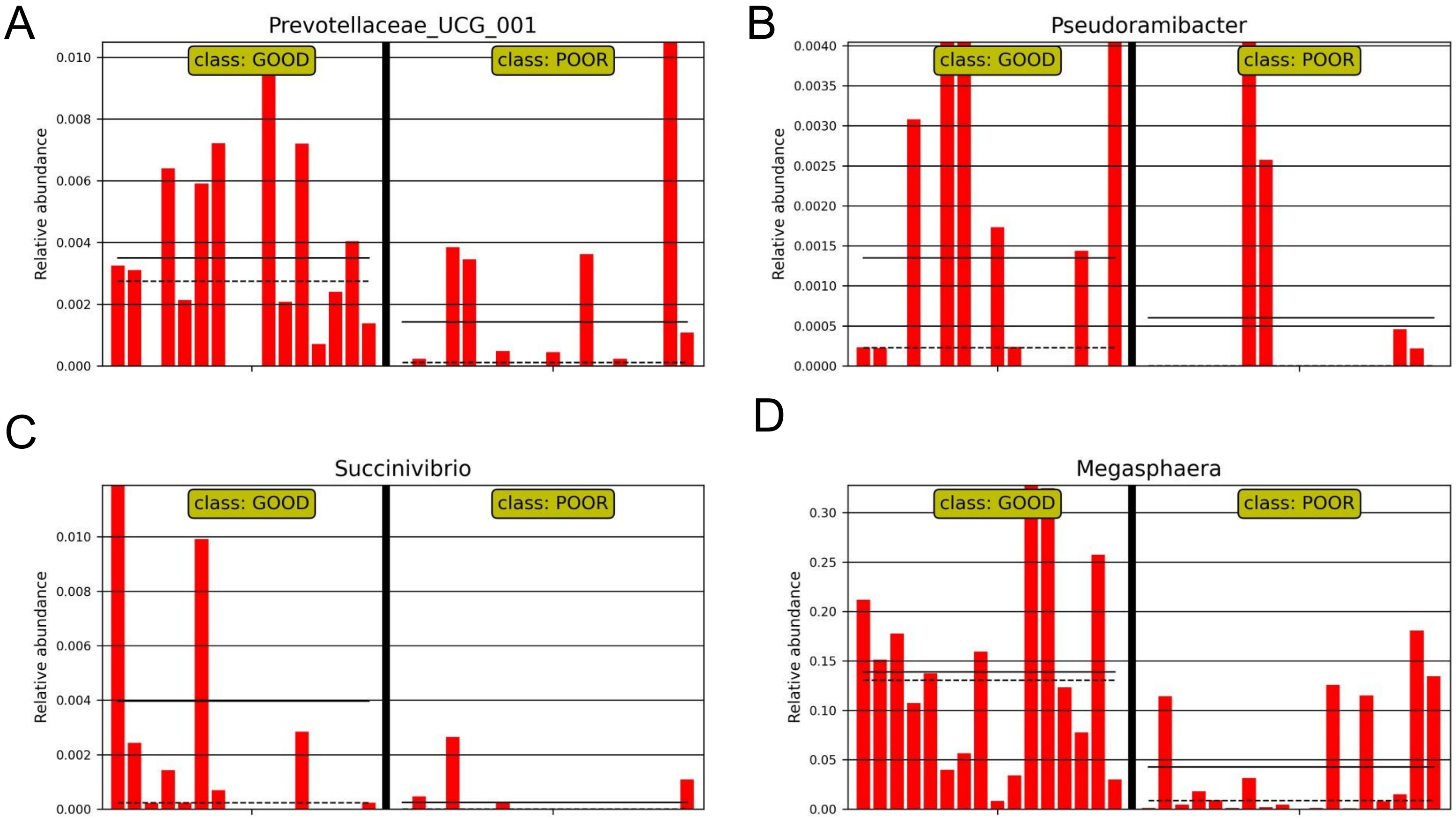

Supplementary Figure 19 | Individual animal relative frequency-based distribution of taxa across barn (SC) conditions (GOOD vs. POOR) at DPC 21. Relative abundance of taxa (**A** – *Prevotellaceae UCG 001*, **B** - *Pseudoramibacter*, **C** - *Succinivibrio*, and **D** - *Megasphaera*) as part of the LEfSe analysis to identify major differentiating taxa between GOOD vs. POOR at the endpoint (DPC 21).

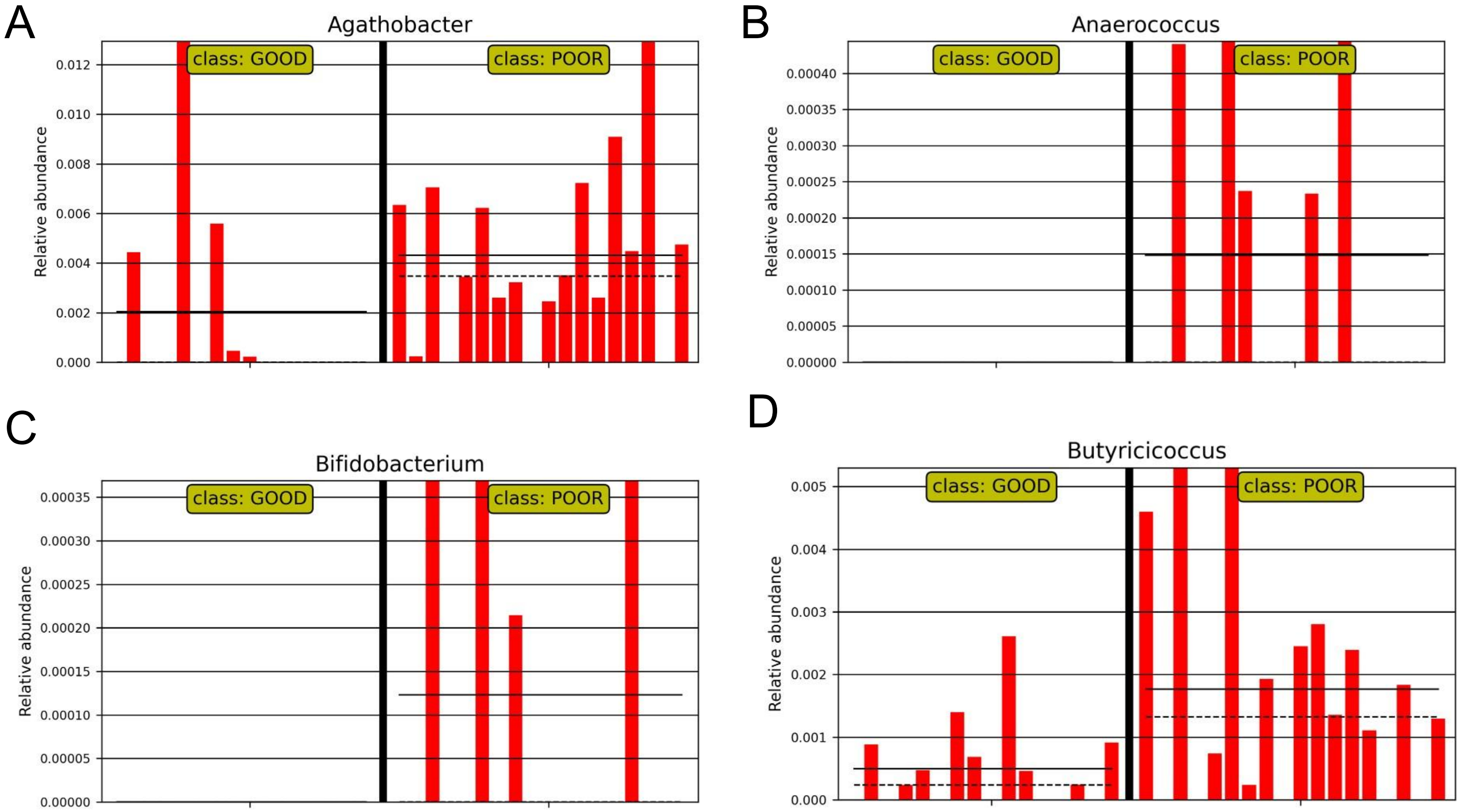

Supplementary Figure 20 | Individual animal relative frequency-based distribution of taxa across barn (SC) conditions (GOOD vs. POOR) at DPC 21. Relative abundance of taxa (**A** – *Agathobacter*, **B** - *Anaerococcus*, **C** - *Bifidobacterium*, and **D** - *Butyricicoccus*) as part of the LEfSe analysis to identify major differentiating taxa between GOOD vs. POOR at the endpoint (DPC 21).

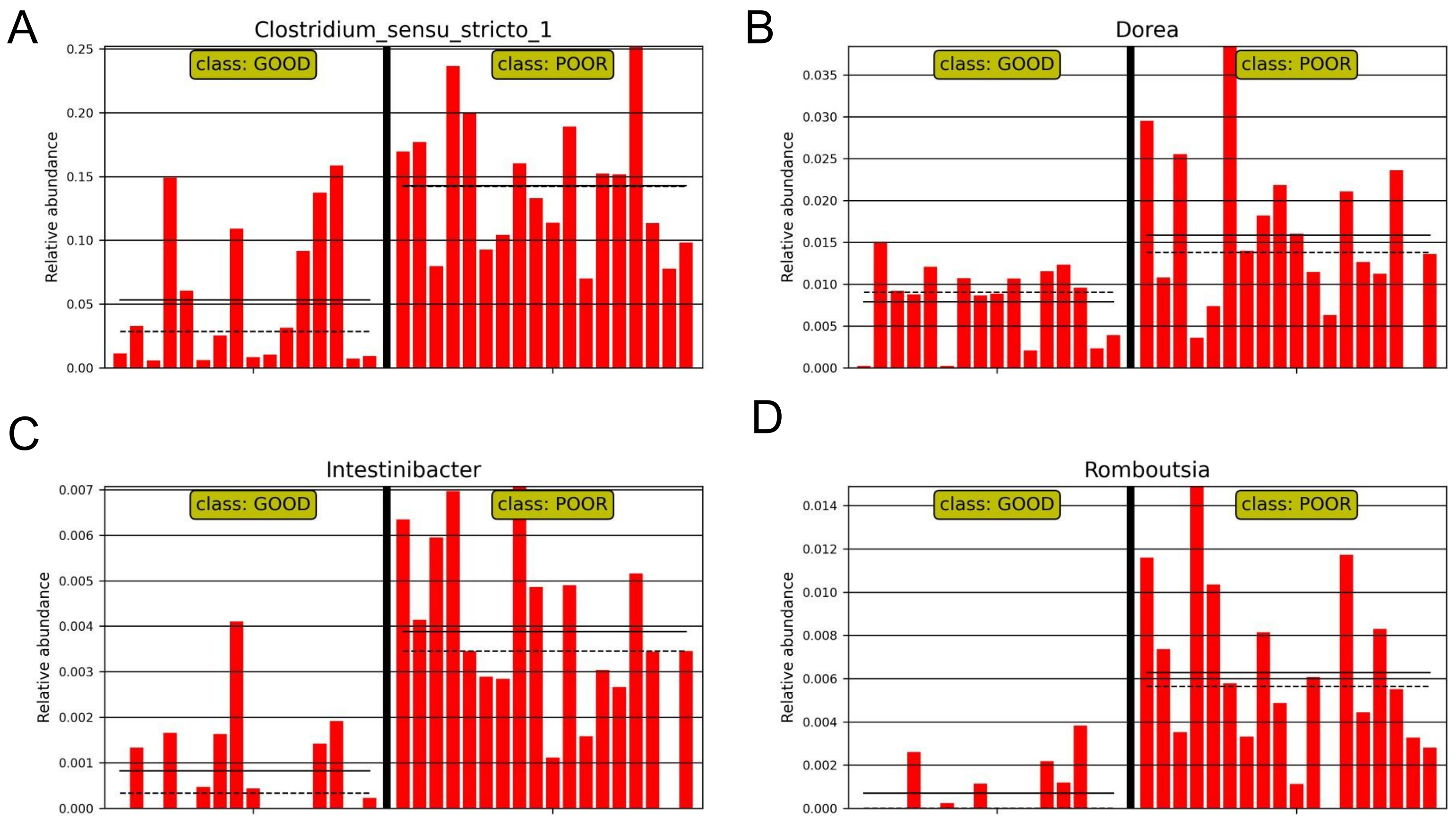

Supplementary Figure 21 | Individual animal relative frequency-based distribution of taxa across barn (SC) conditions (GOOD vs. POOR) at DPC 21. Relative abundance of taxa (**A** – *Clostridium stricto sensu 1*, **B** - *Dorea*, **C** - *Instestinibacter*, and **D** - *Romboutsia*) as part of the LEfSe analysis to identify major differentiating taxa between GOOD vs. POOR at the endpoint (DPC 21).

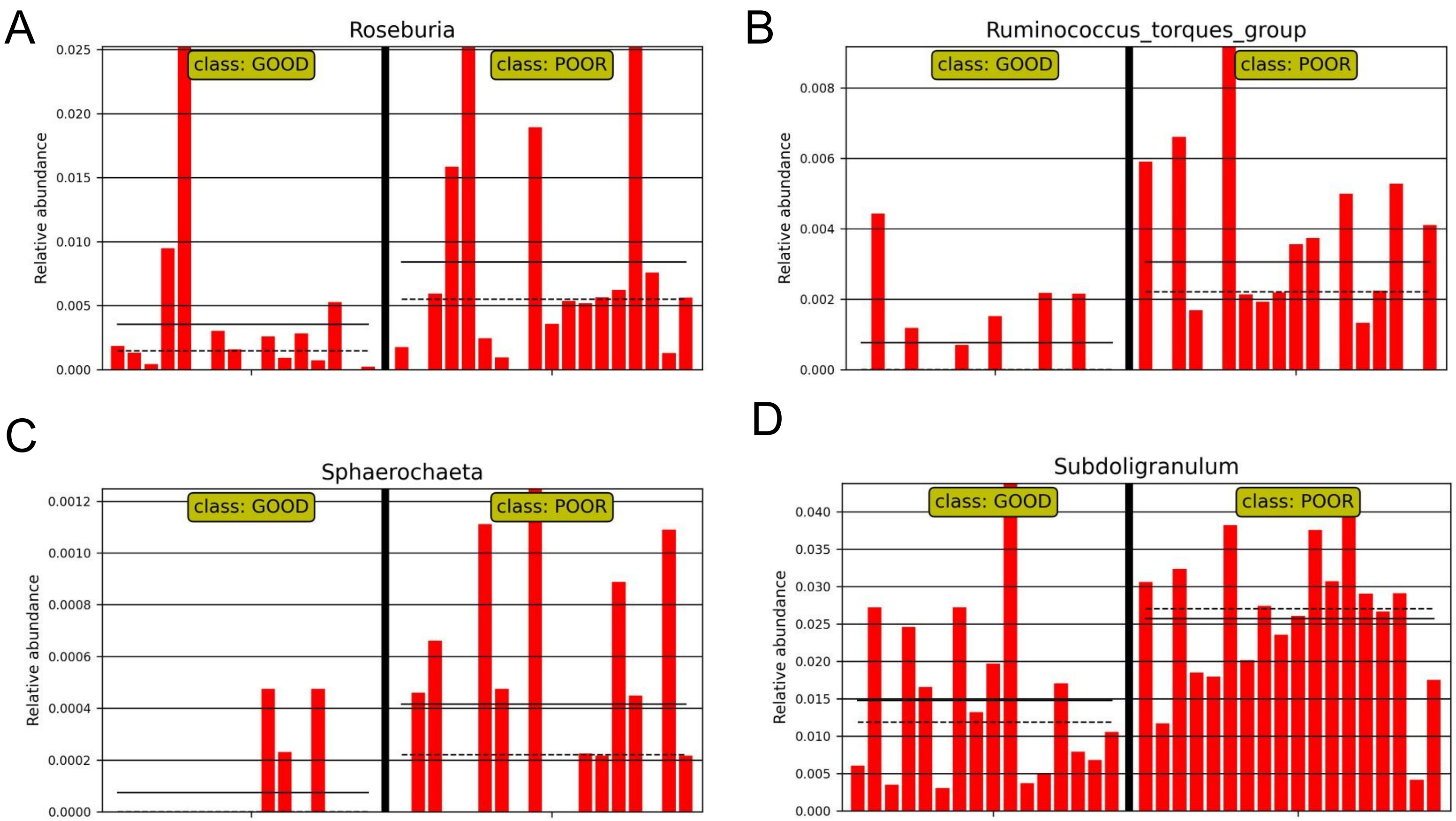

Supplementary Figure 22 | Individual animal relative frequency-based distribution of taxa across barn (SC) conditions (GOOD vs. POOR) at DPC 21. Relative abundance of taxa (**A** – *Roseburia*, **B** – *Ruminococcus torques group*, **C** - *Sphaerochaeta*, and **D** - *Subdoligranulum*) as part of the LEfSe analysis to identify major differentiating taxa between GOOD vs. POOR at the endpoint (DPC 21).

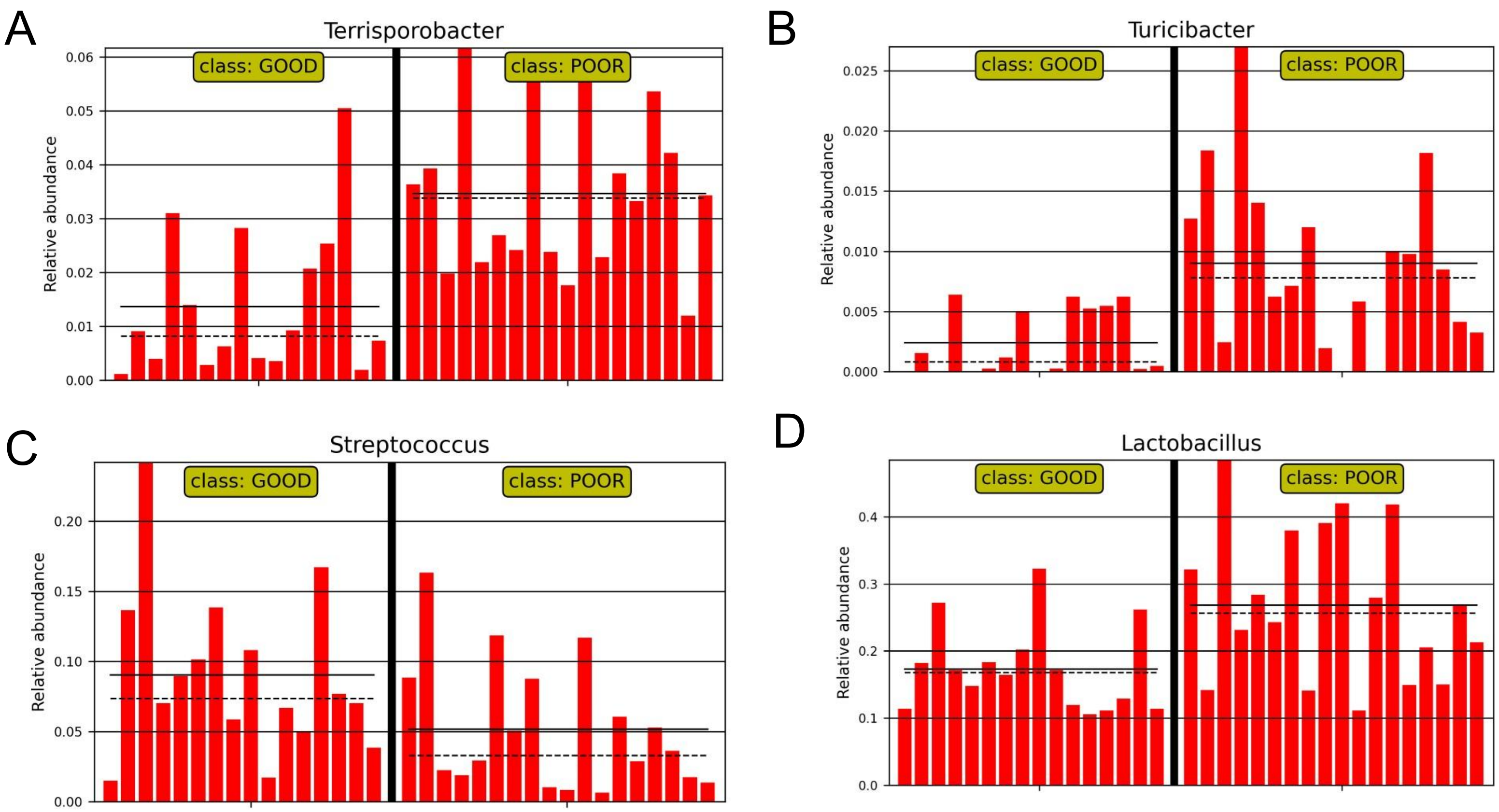

Supplementary Figure 23 | Individual animal relative frequency-based distribution of taxa across barn (SC) conditions (GOOD vs. POOR) at DPC 21. Relative abundance of taxa (**A** – *Terrisporobacter*, **B** - *Turicibacter*, **C** - *Streptococcus*, and **D** - *Lactobacillus*) as part of the LEfSe analysis to identify major differentiating taxa between GOOD vs. POOR at the endpoint (DPC 21).

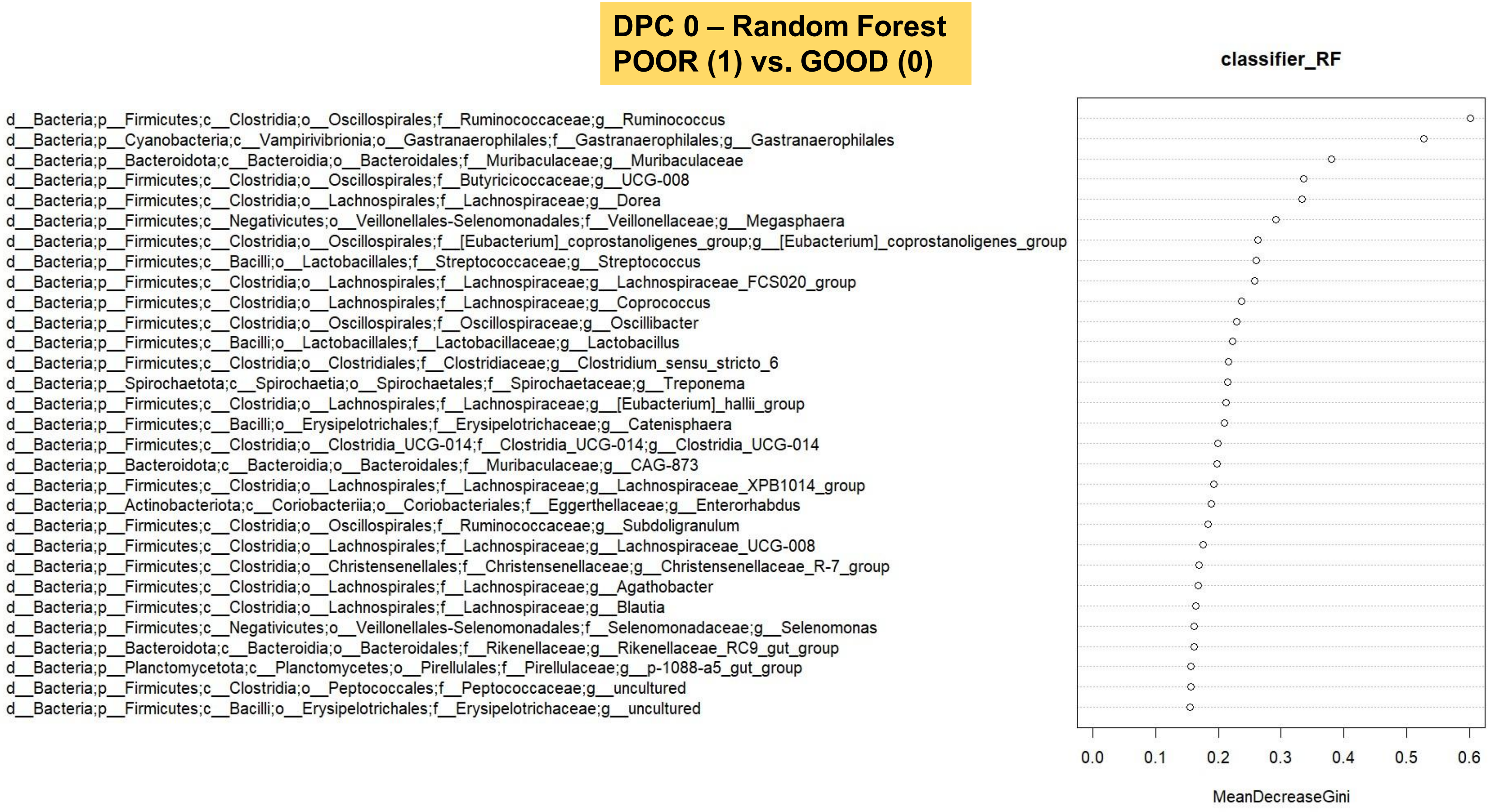

Supplementary Figure 24 | Random Forest classification results comparing Barn-SC POOR vs. GOOD at DPC 0. Predictor variables were coded as 1 (POOR) and 0 (GOOD) as factor. The importance variable metric used was mean decrease impurity or Gini importance (x-axis).

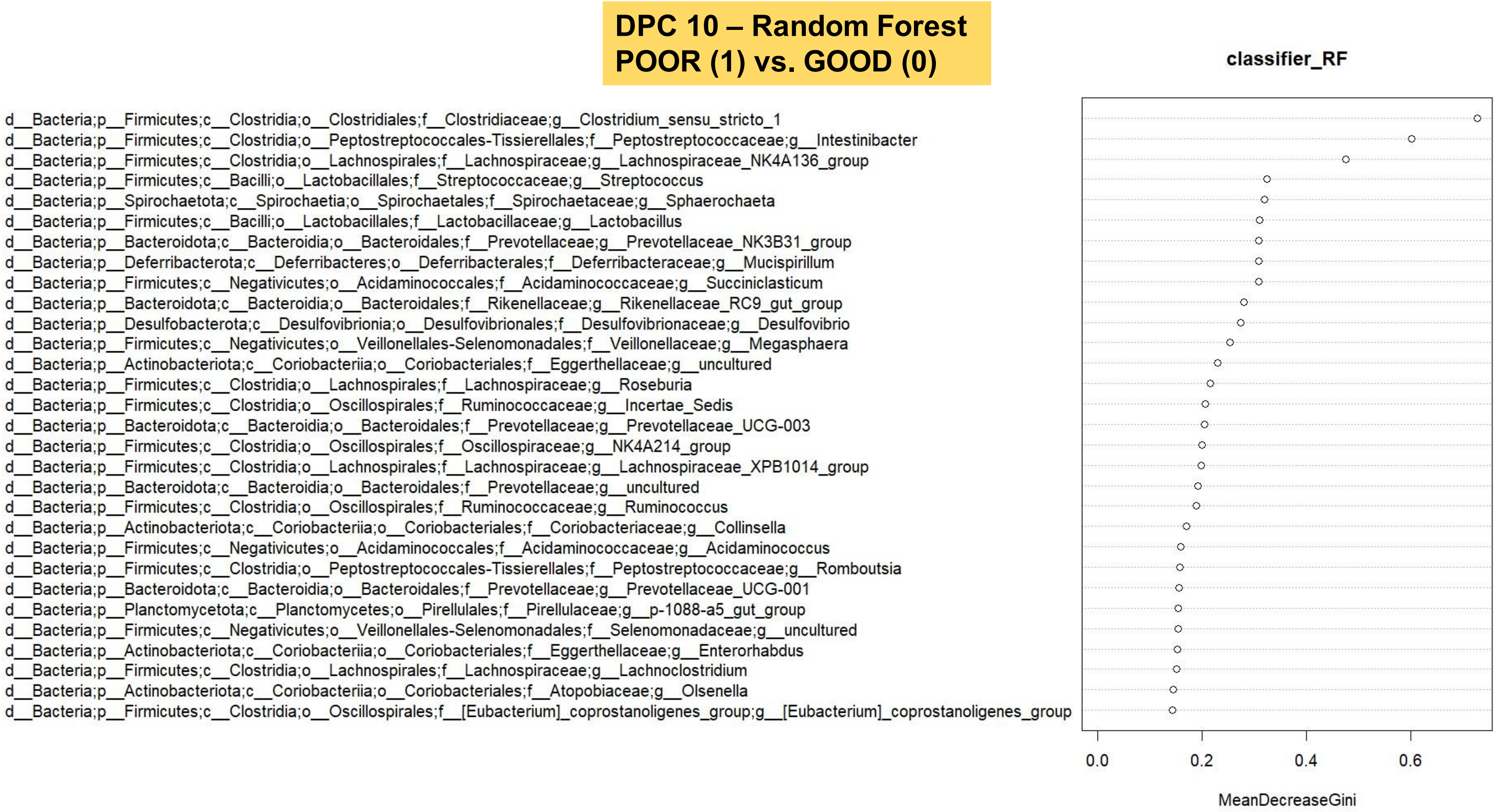

Supplementary Figure 25 | Random Forest classification results comparing Barn-SC POOR vs. GOOD at DPC 10. Predictor variables were coded as 1 (POOR) and 0 (GOOD) as factor. The importance variable metric used was mean decrease impurity or Gini importance (x-axis).

DPC 21 – Random Forest
POOR (1) vs. GOOD (0)

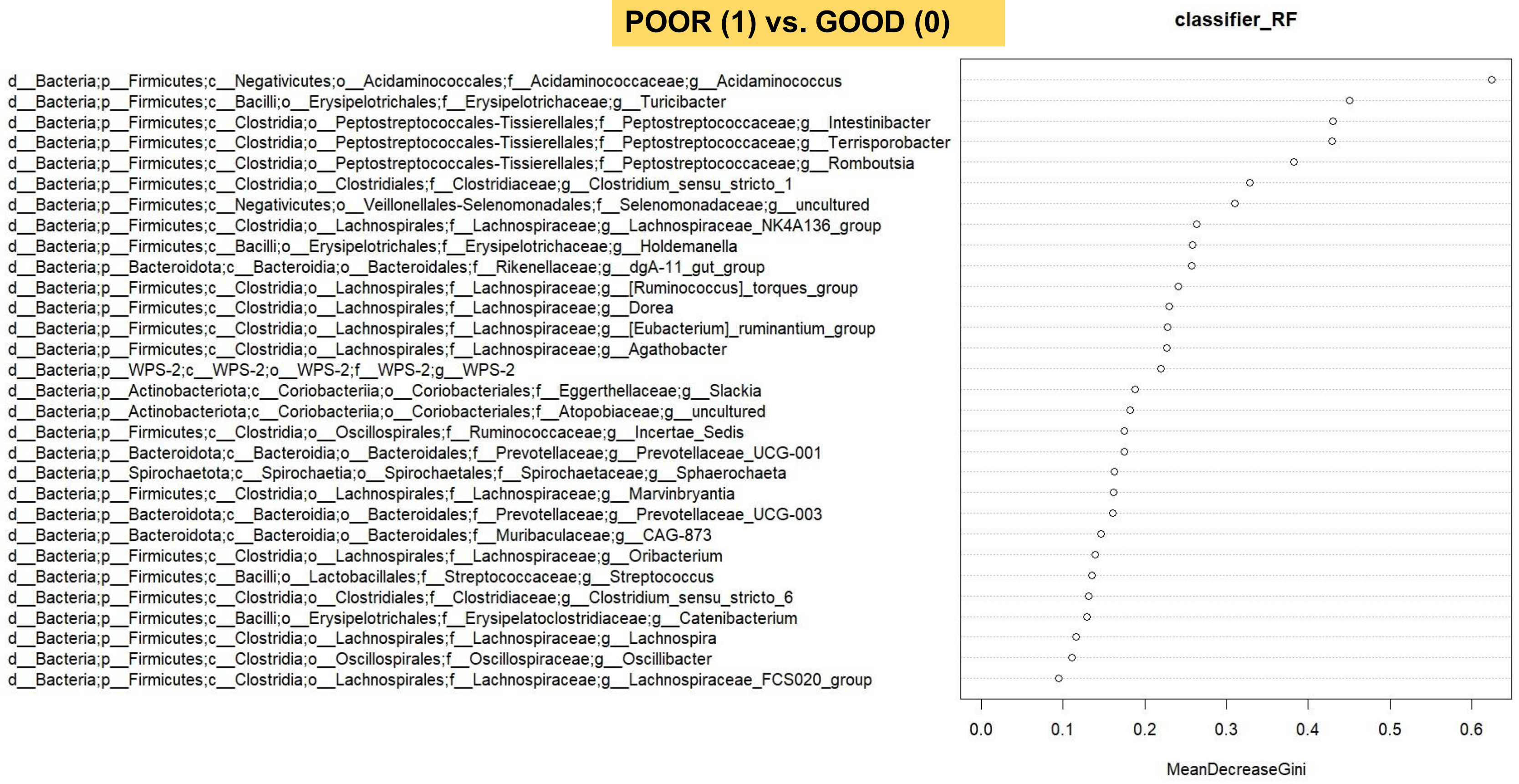

Supplementary Figure 26 | Random Forest classification results comparing Barn-SC POOR vs. GOOD at DPC 21. Predictor variables were coded as 1 (POOR) and 0 (GOOD) as factor. The importance variable metric used was mean decrease impurity or Gini importance (x-axis).

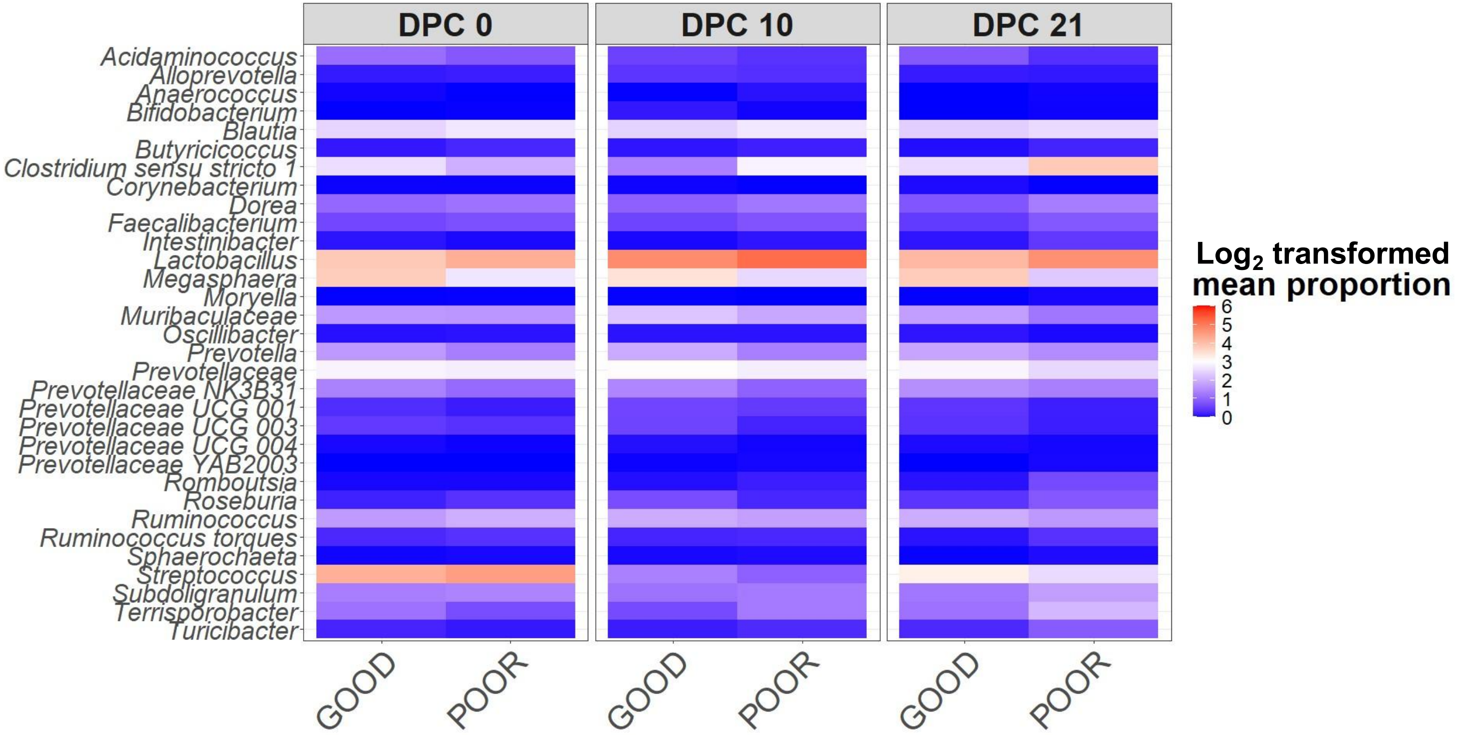

Supplementary Figure 27 | Fecal microbiome keystone taxa distribution across barn (SC) groups by DPC. Log_2 transformed mean proportion of keystone taxa across housing sanitary conditions (GOOD vs. POOR) across DPC 0, 10, and 21. Rarified counts of each taxon were used as input data. Taxon were grouped at different levels of taxonomic resolution capturing the most important ones present in the data.

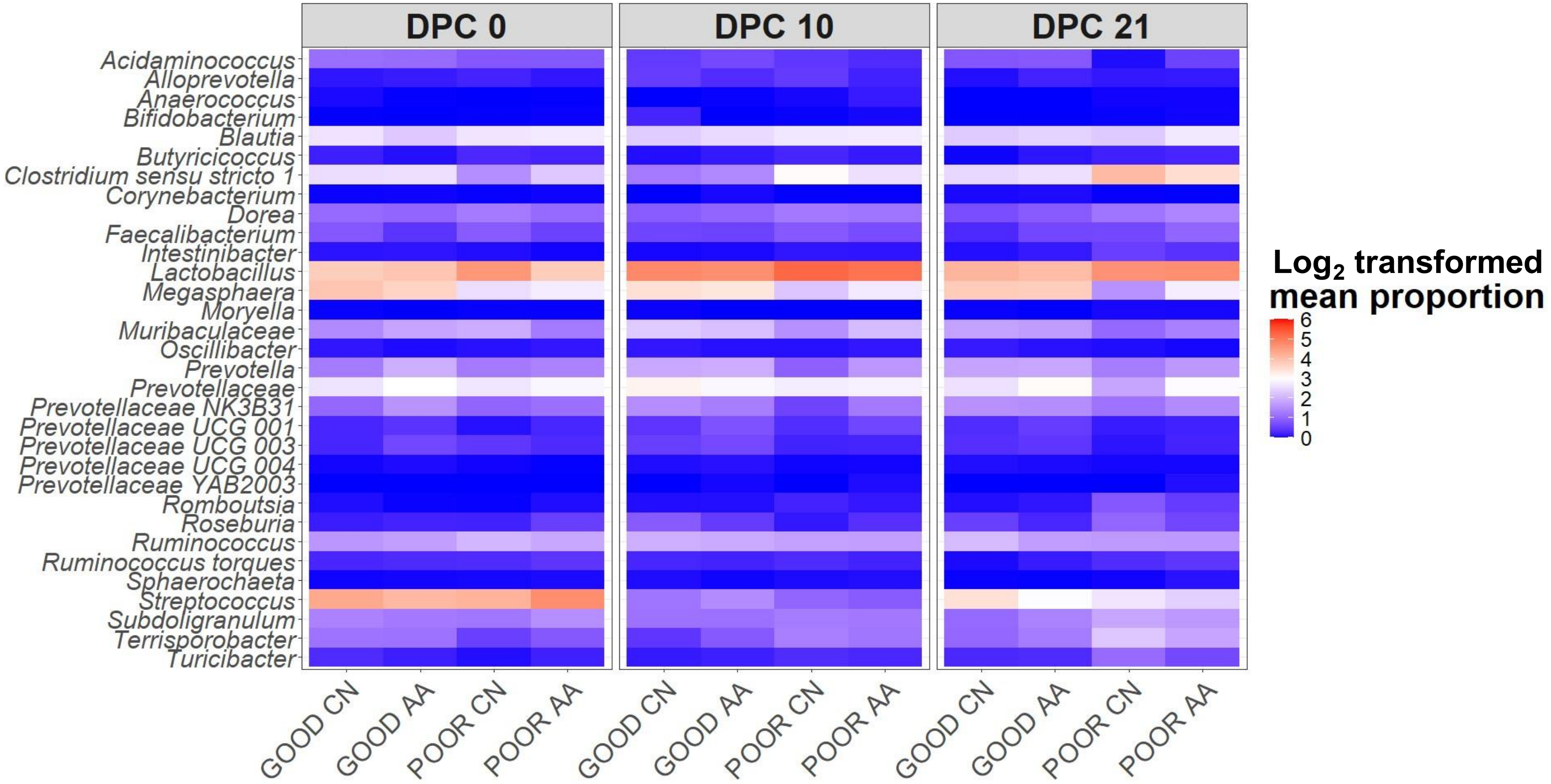

Supplementary Figure 28 | Fecal microbiome keystone taxa distribution across all treatments combining both barn (SC) and diet groups by DPC. Log_2 transformed mean proportion of keystone taxa across all treatments combining housing sanitary conditions (GOOD vs. POOR) and diet (CON vs. AA) across DPC 0, 10, and 21. Rarified counts of each taxon were used as input data. Taxon were grouped at different levels of taxonomic resolution capturing the most important ones present in the data.

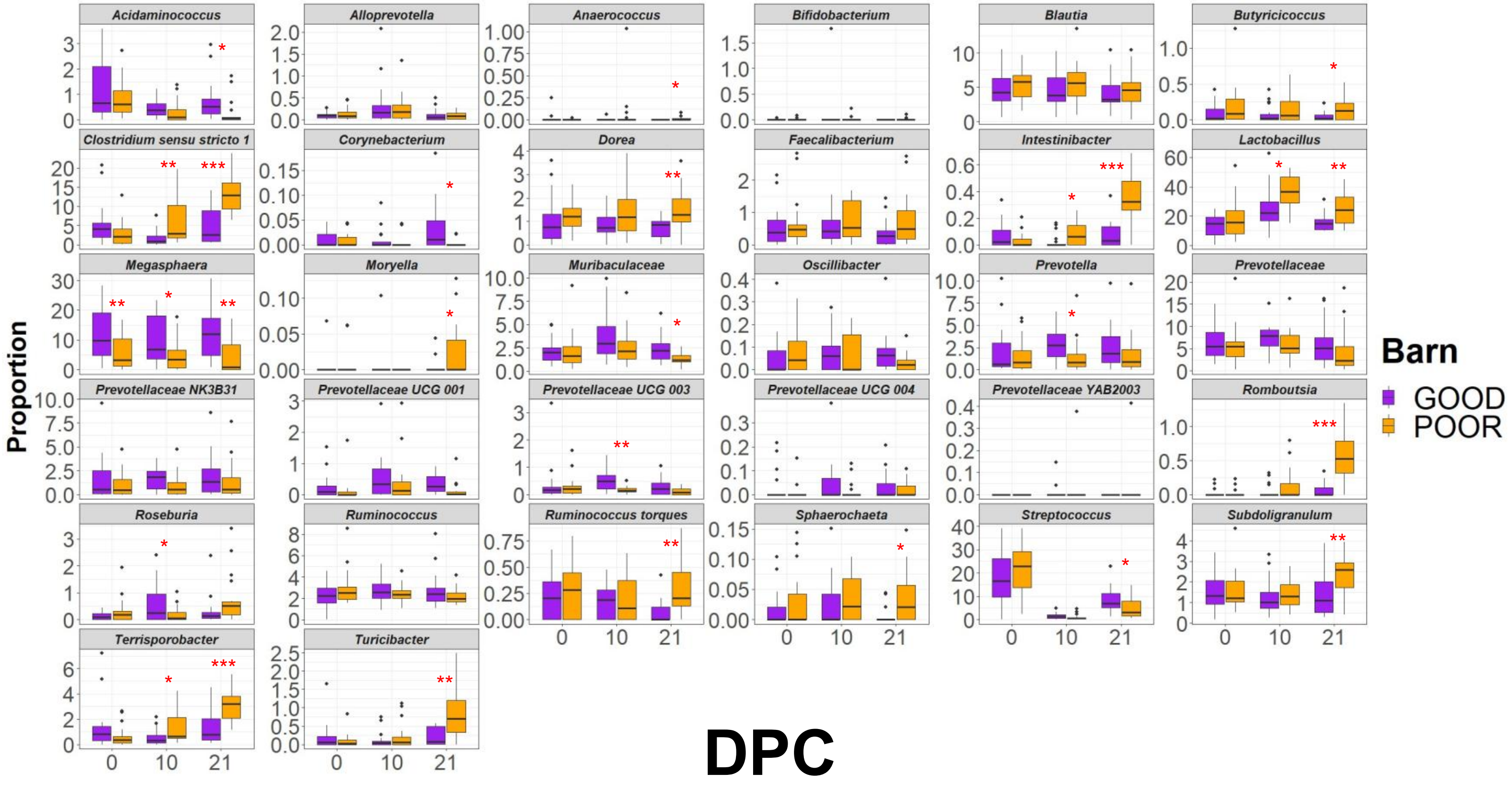

Supplementary Figure 29 | Keystone taxa proportion-based distribution across fecal samples for pigs housed under GOOD vs. POOR sanitary conditions across DPC. A two-sided T-test was used to assess difference between housing sanitary conditions at each DPC ($P < 0.05$). * For $P < 0.05$; ** for $P < 0.01$, and *** for $P < 0.001$. Rarefied counts were used for proportion calculations.

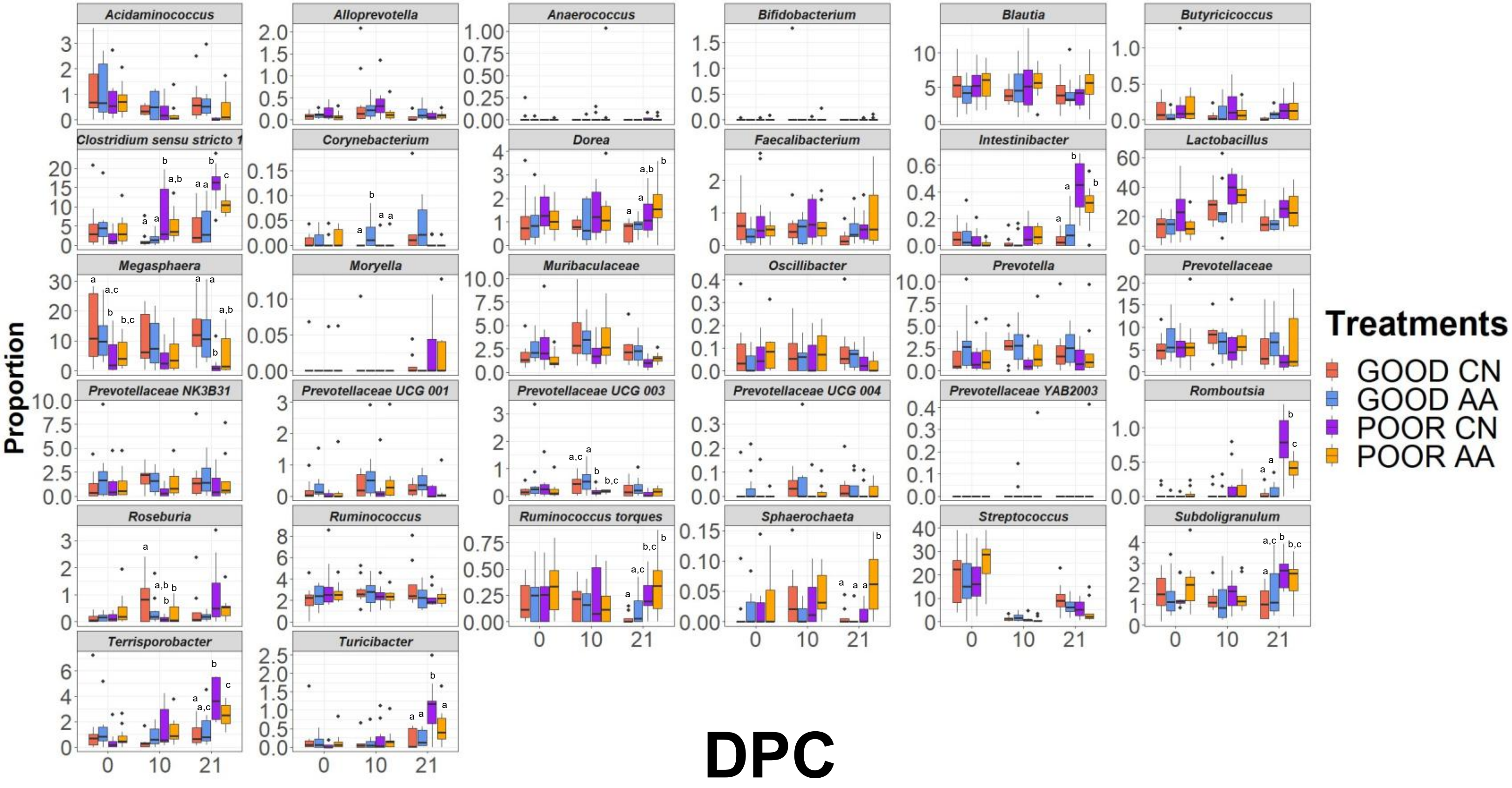

Supplementary Figure 30 | Keystone taxa proportion-based distribution across all treatments combining barn (SC) (GOOD vs. POOR) and diet (CN vs. AA) across DPC. A one-way ANOVA was initially used to assess the treatment effect, followed by a pairwise two-sided T-test to assess differences between treatments at each DPC ($P < 0.05$). Superscript letters represent differences between treatment groups by DPC, which were only measured if $p < 0.05$ for ANOVA (effect of treatments). Rarefied counts were used for proportion calculations.

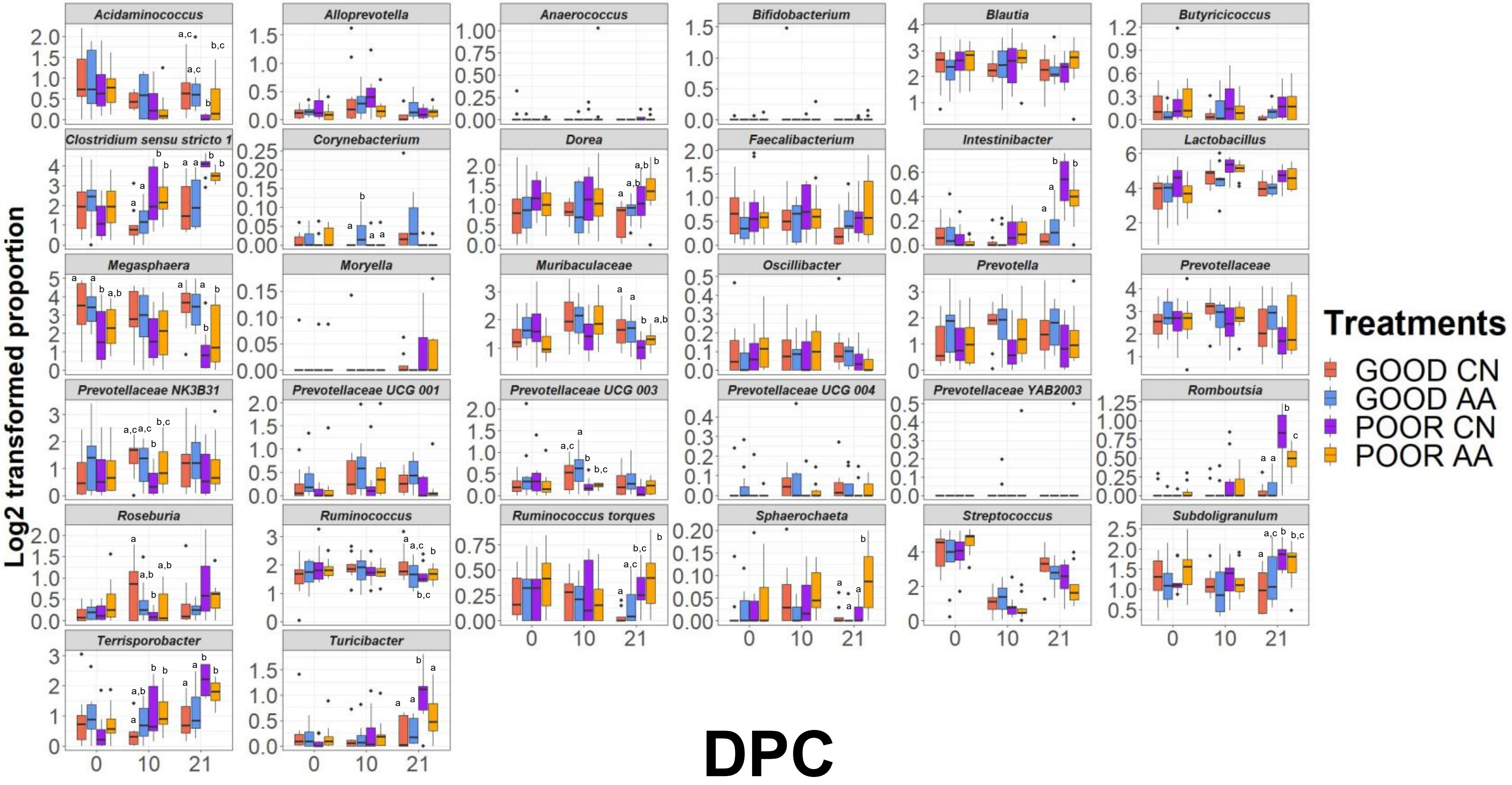

Supplementary Figure 31 | Keystone taxa log_2 proportion-based distribution across all treatments combining barn (SC) (GOOD vs. POOR) and diet (CN vs. AA) across DPC. A one-way ANOVA was initially used to assess the treatment effect, followed by a pairwise two-sided T-test to assess differences between treatments at each DPC ($P < 0.05$). Superscript letters represent differences between treatment groups by DPC, which were only measured if $p < 0.05$ for ANOVA (effect of treatments). Rarefied counts were used for proportion calculations prior to log_2 transformation of the data.

7

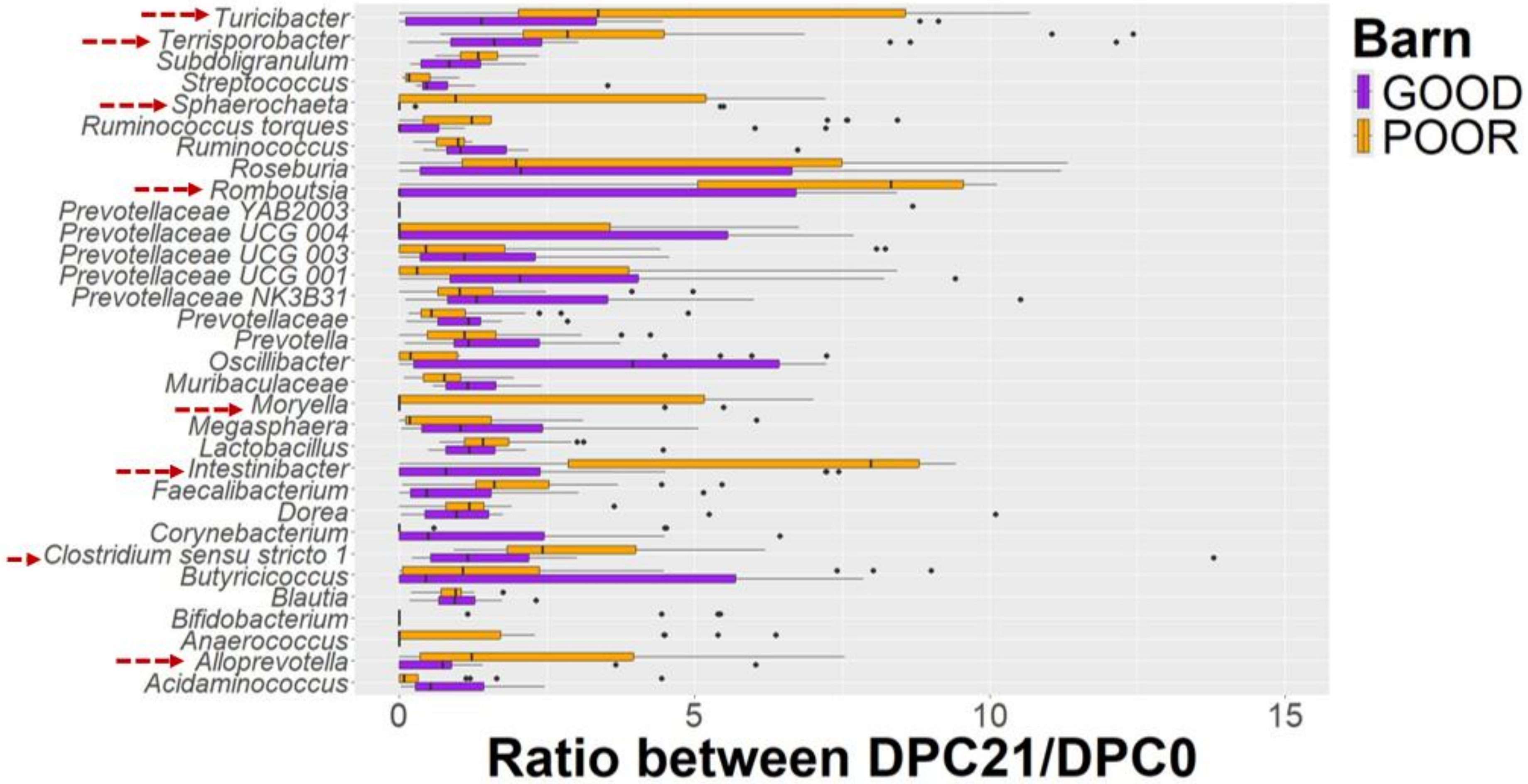
Turicibacter
Terrisporobacter
Subdoligranulum
Streptococcus
Sphaerochaeta
Ruminococcus torques
Ruminococcus
Roseburia
Romboutsia
Prevotellaceae YAB2003
Prevotellaceae UCG 004
Prevotellaceae UCG 003
Prevotellaceae UCG 001
Prevotellaceae NK3B31
Prevotellaceae
Prevotella
Oscillibacter
Muribaculaceae
Moryella
Megasphaera
Lactobacillus
Intestinibacter
Faecalibacterium
Dorea
Corynebacterium
Clostridium sensu stricto 1
Butyricicoccus
Blautia
Bifidobacterium
Anaerococcus
Alloprevotella
Acidaminococcus
0
5
10
15
Ratio between DPC21/DPC0
Barn
GOOD
POOR

Supplementary Figure 32 | Box-and-whiskers plot showing a keystone-based taxa enrichment analysis at endpoint in comparison to baseline between pigs housed under GOOD vs. POOR sanitary conditions. Log2 based transformed proportional ratio of keystone taxa across sanitary conditions (Barn) are depicted in the plot. All ratios were calculated using rarefied data as input, followed by calculating the relative abundance of each taxon by animal DPC 21 in relationship to DPC 0, and then a log2 transformation was done. A value of 0.01 was inputted for proportions that were zero at DPC 0. Red dotted arrows on the left side mark all taxa with a marked enrichment at endpoint for POOR sanitary condition.

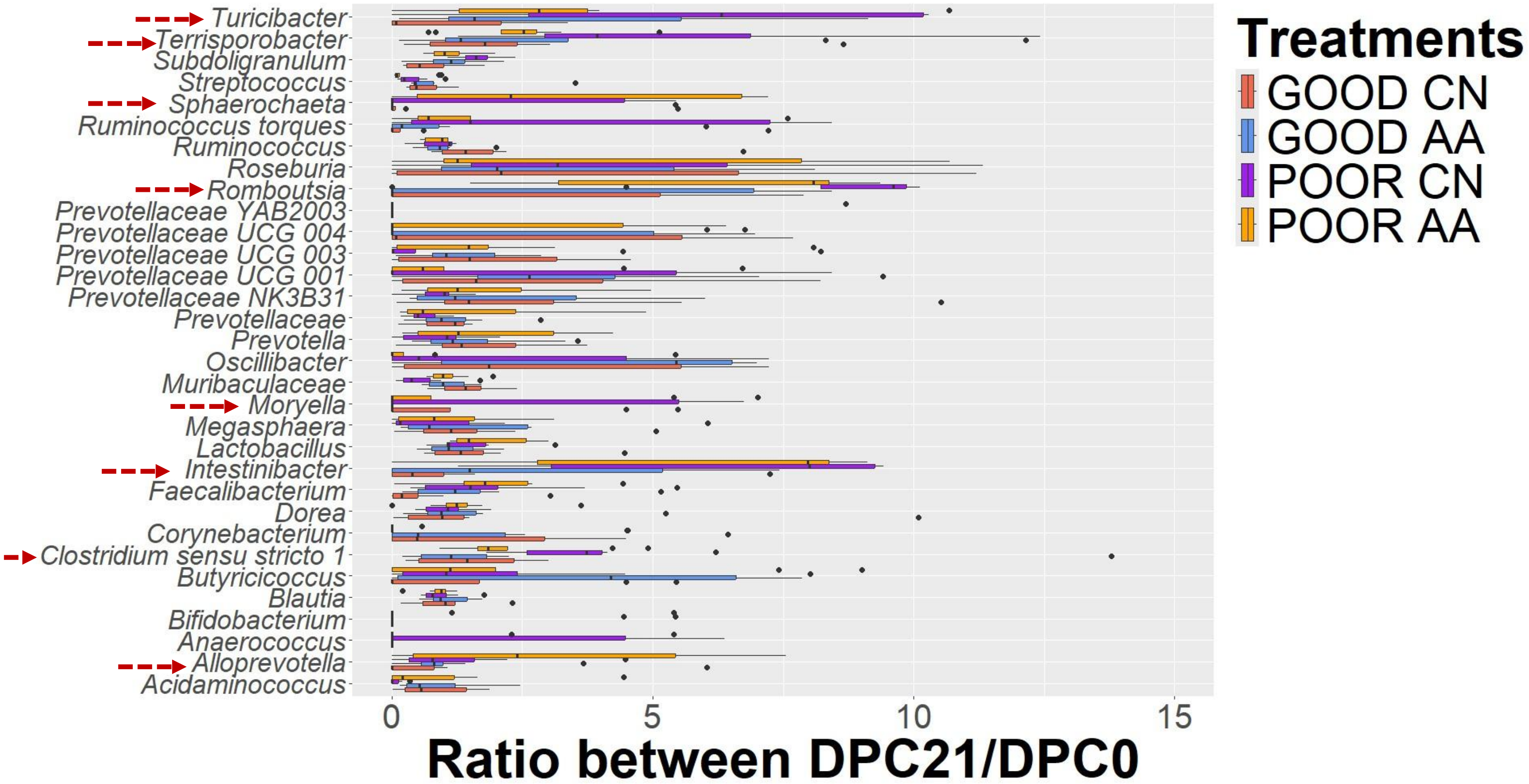

Turicibacter
Terrisporobacter
Subdoligranulum
Streptococcus
Sphaerochaeta
Ruminococcus torques
Ruminococcus
Roseburia
Romboutsia
Prevotellaceae YAB2003
Prevotellaceae UCG 004
Prevotellaceae UCG 003
Prevotellaceae UCG 001
Prevotellaceae NK3B31
Prevotellaceae
Prevotella
Oscillibacter
Muribaculaceae
Moryella
Megasphaera
Lactobacillus
Intestinibacter
Faecalibacterium
Dorea
Corynebacterium
Clostridium sensu stricto 1
Butyricicoccus
Blautia
Bifidobacterium
Anaerococcus
Alloprevotella
Acidaminococcus
0
5
10
15
Ratio between DPC21/DPC0
Treatments
GOOD CN
GOOD AA
POOR CN
POOR AA

Supplementary Figure 33 | Box-and-whiskers plot showing a keystone-based taxa enrichment analysis at endpoint in comparison to baseline across all treatments combining Barn (GOOD vs. POOR) and diet (CN vs. AA). Log_2 based transformed proportional ratio of keystone taxa across all treatments are depicted in the plot. All ratios were calculated using rarefied data as input, followed by calculating the relative abundance of each taxon by animal DPC 21 in relationship to DPC 0, and then a log_2 transformation was done. A value of 0.01 was inputted for proportions that were zero at DPC 0. Red dotted arrows on the left side mark all taxa with a marked enrichment at endpoint for POOR sanitary condition for both diets.

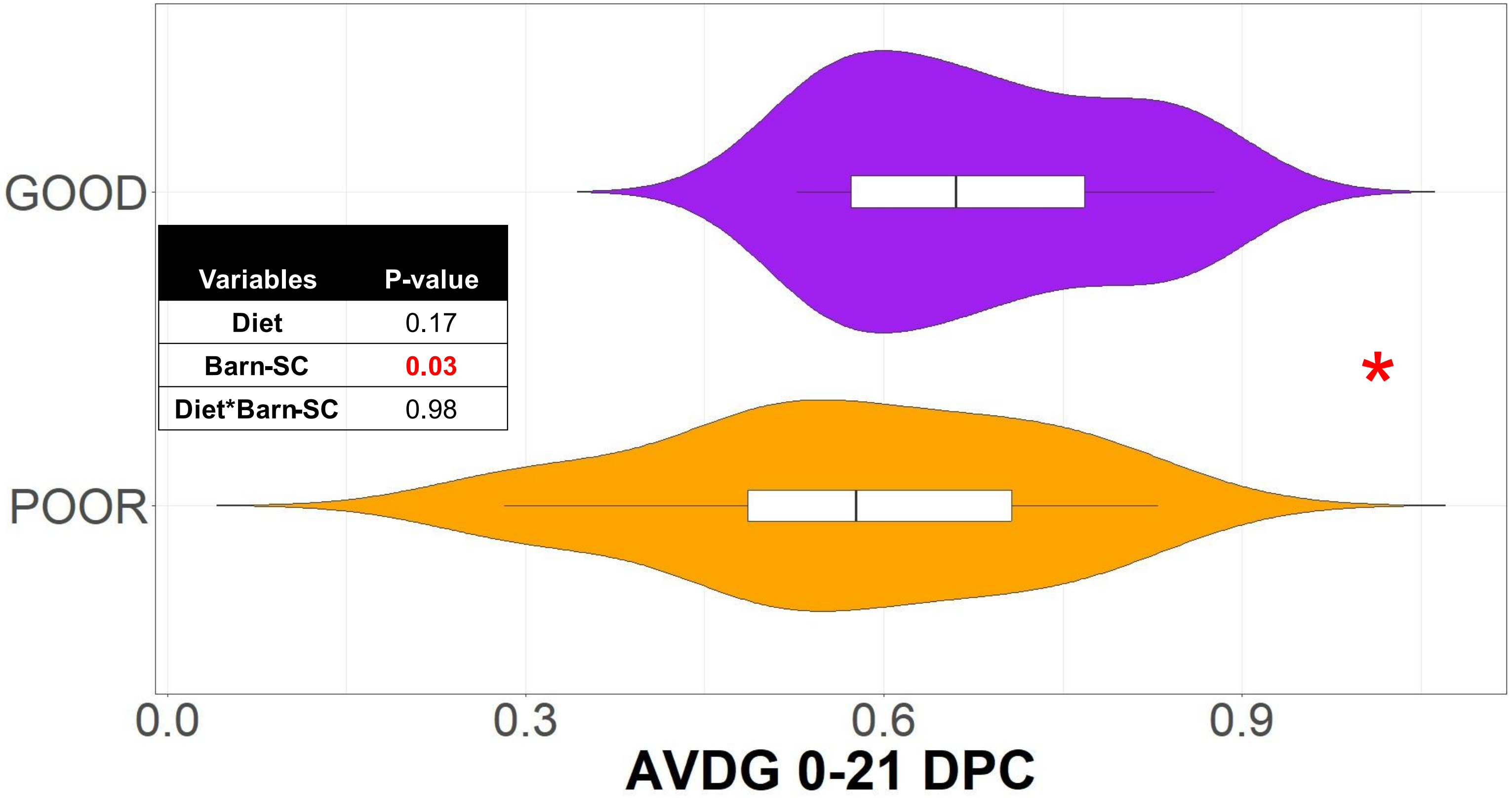

Supplementary Figure 34 | Violin and box-and-whiskers plot showing the distribution average daily gain (weight) for pigs from DPC 0 to 21 between GOOD vs. POOR Barn-SC. An ANOVA model was used to assess the significant effect of each main variable and interaction ($P < 0.05$). * For $P < 0.05$; ** for $P < 0.01$, and *** for $P < 0.001$.

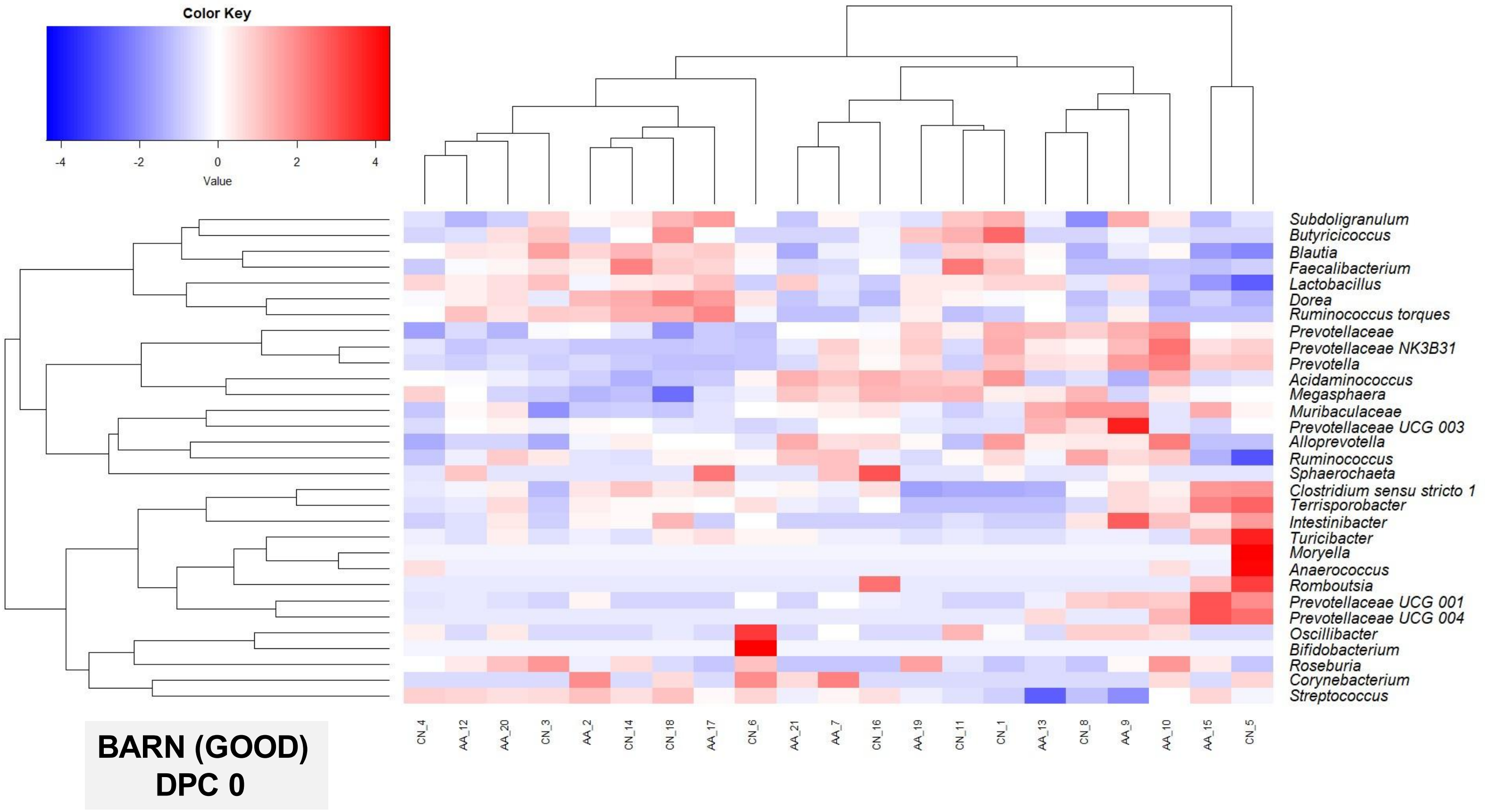

Supplementary Figure 35 | Heatmap clustering major taxa abundance in fecal microbiome of pigs that will be housed in a barn under GOOD sanitary condition (SC) at DPC 0 and that were fed a control (CN) or a diet supplemented with functional amino acids supplemented (AA). Each row represents a distinct taxon, while each column represents an individual pig housed in a barn under GOOD SC and that will feed a CN or AA diet over the experiment. The stronger red color, the more abundant the taxon is; while the stronger blue color, less abundant the taxon is. Log_2 rarefied transformed proportions were used as input data for scaling.

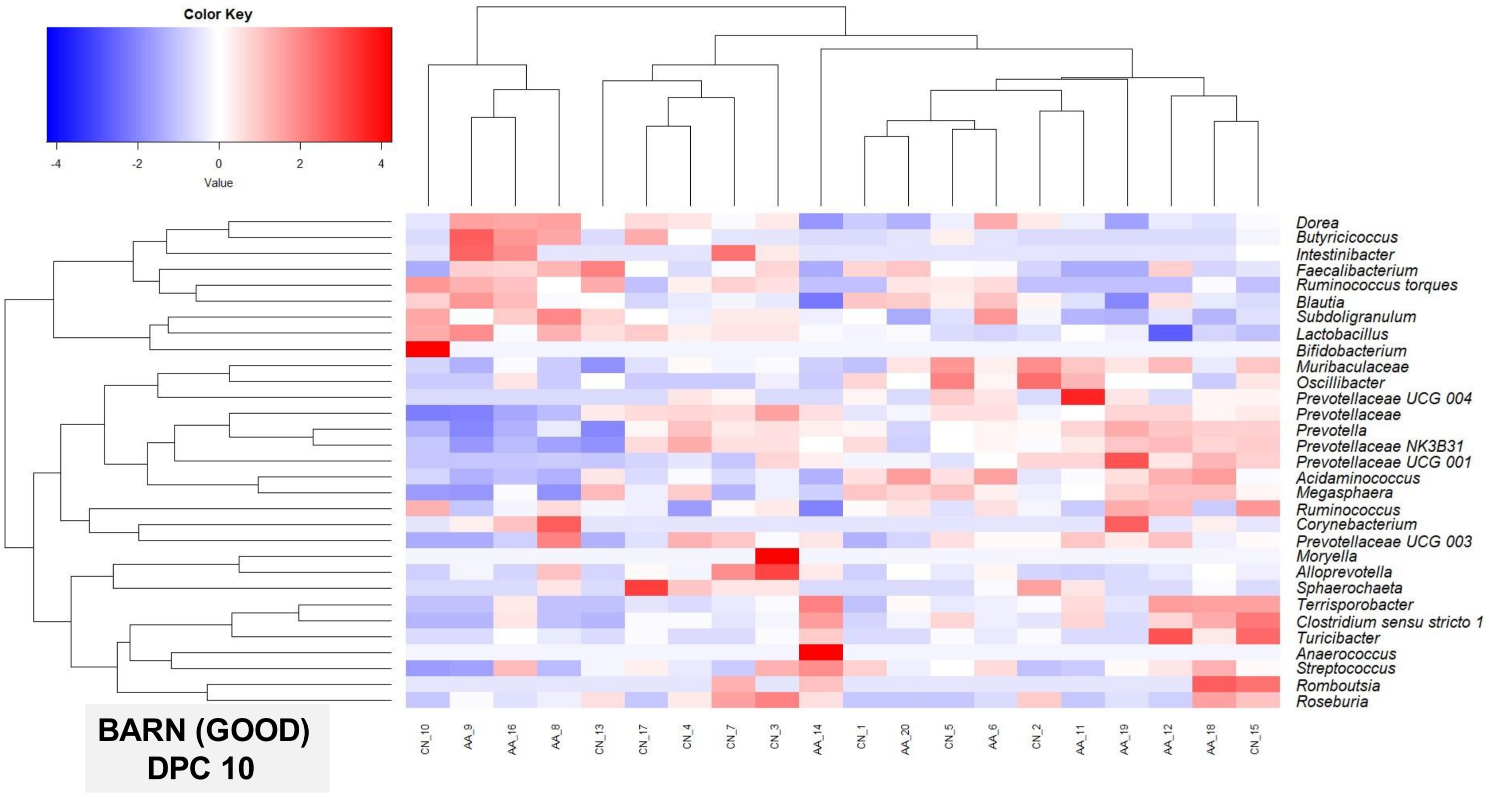

Supplementary Figure 36 | Heatmap clustering major taxa abundance in fecal microbiome of pigs that will be housed in a barn under GOOD sanitary condition (SC) at DPC 10 and that were fed a control (CN) or a diet supplemented with functional amino acids supplemented (AA). Each row represents a distinct taxon, while each column represents an individual pig housed in a barn under GOOD SC and that will feed a CN or AA diet over the experiment. The stronger red color, the more abundant the taxon is; while the stronger blue color, less abundant the taxon is. Log_2 rarefied transformed proportions were used as input data for scaling.

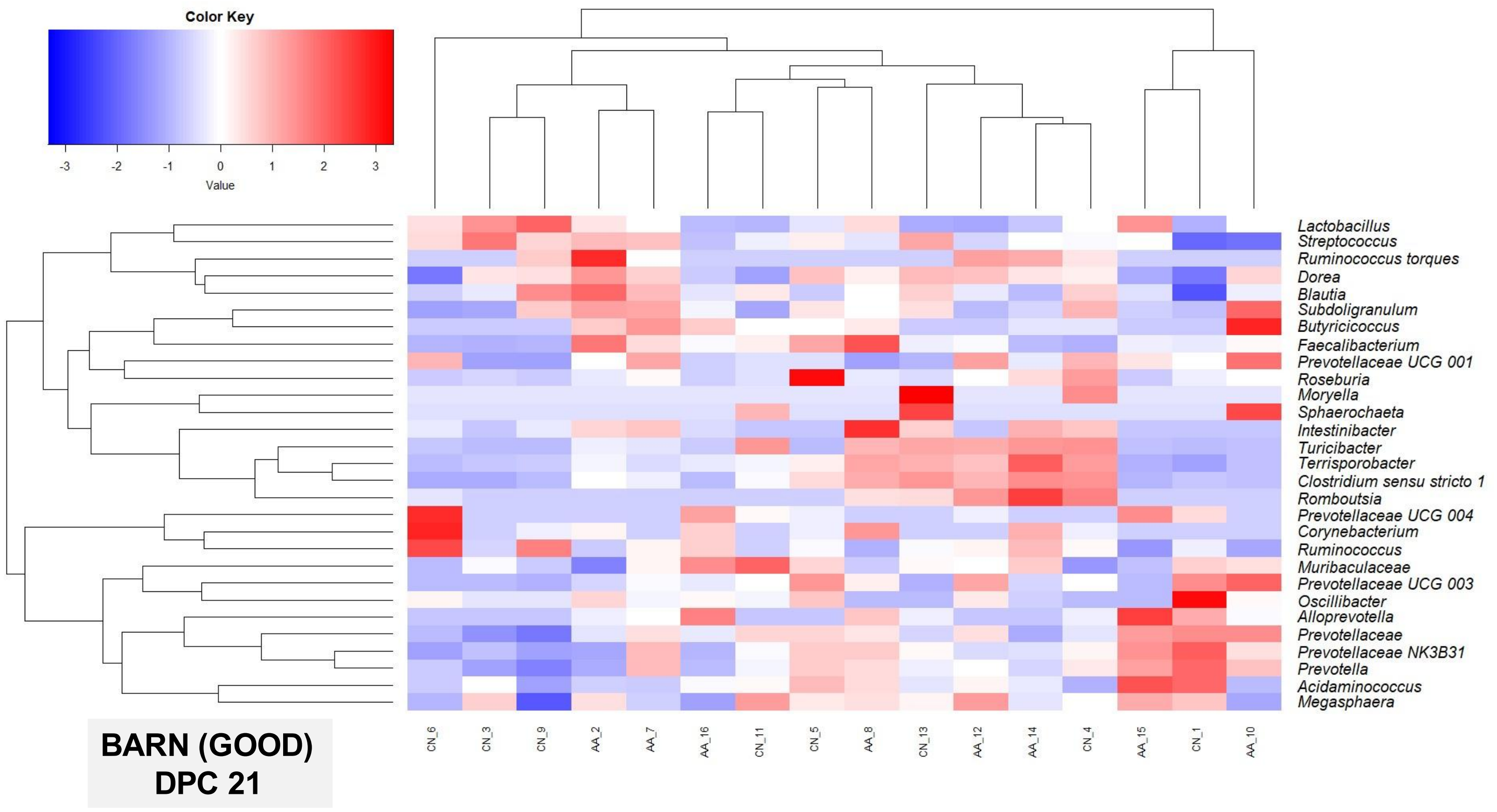

Supplementary Figure 37 | Heatmap clustering major taxa abundance in fecal microbiome of pigs that will be housed in a barn under GOOD sanitary condition (SC) at DPC 21 and that were fed a control (CN) or a diet supplemented with functional amino acids supplemented (AA). Each row represents a distinct taxon, while each column represents an individual pig housed in a barn under GOOD SC and that will feed a CN or AA diet over the experiment. The stronger red color, the more abundant the taxon is; while the stronger blue color, less abundant the taxon is. Log_2 rarefied transformed proportions were used as input data for scaling.

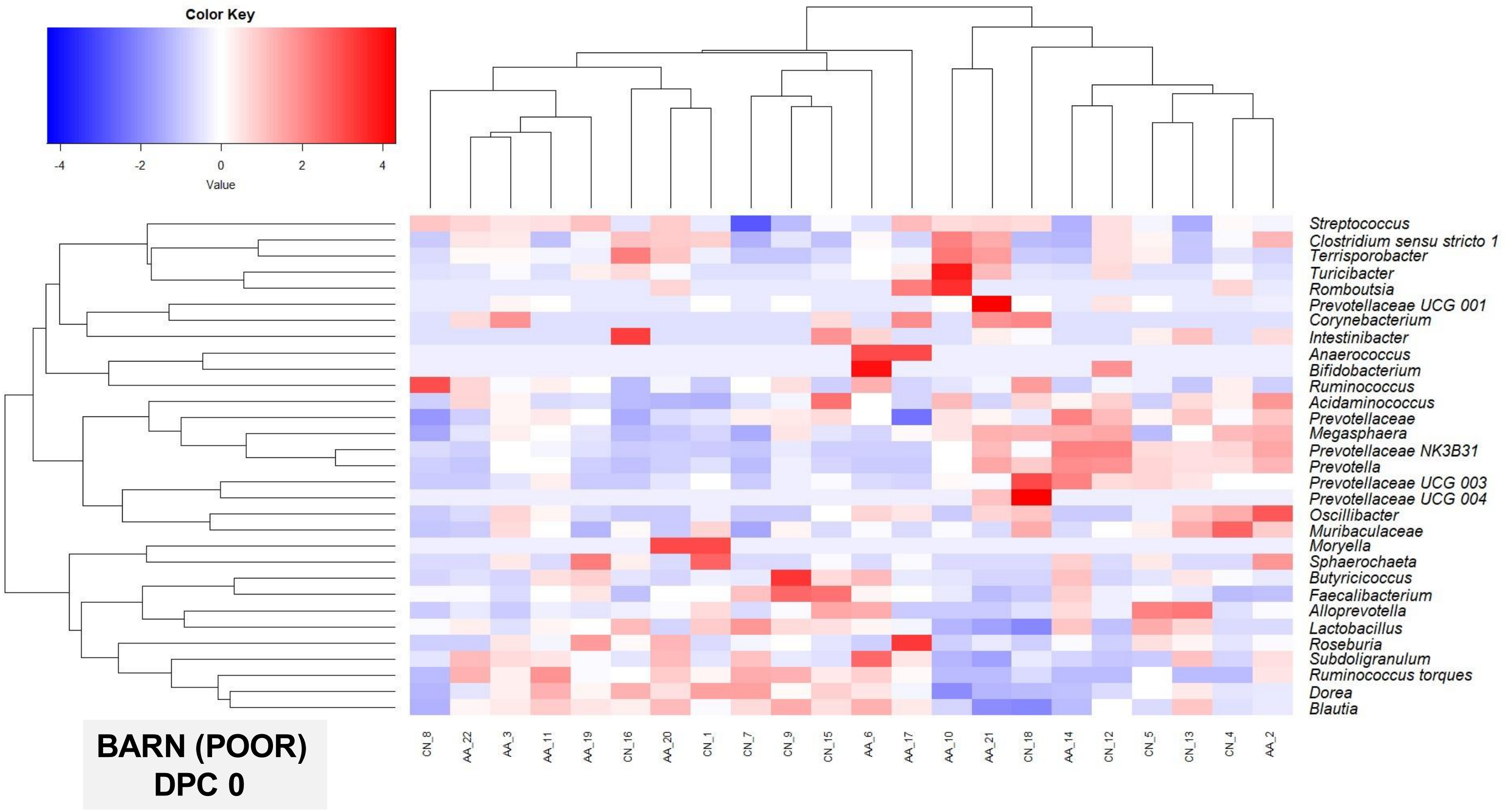

Supplementary Figure 38 | Heatmap clustering major taxa abundance in fecal microbiome of pigs that will be housed in a barn under POOR sanitary condition (SC) at DPC 0 and that were fed a control (CN) or a diet supplemented with functional amino acids supplemented (AA). Each row represents a distinct taxon, while each column represents an individual pig housed in a barn under POOR SC and that will feed a CN or AA diet over the experiment. The stronger red color, the more abundant the taxon is; while the stronger blue color, less abundant the taxon is. Log_2 rarefied transformed proportions were used as input data for scaling.

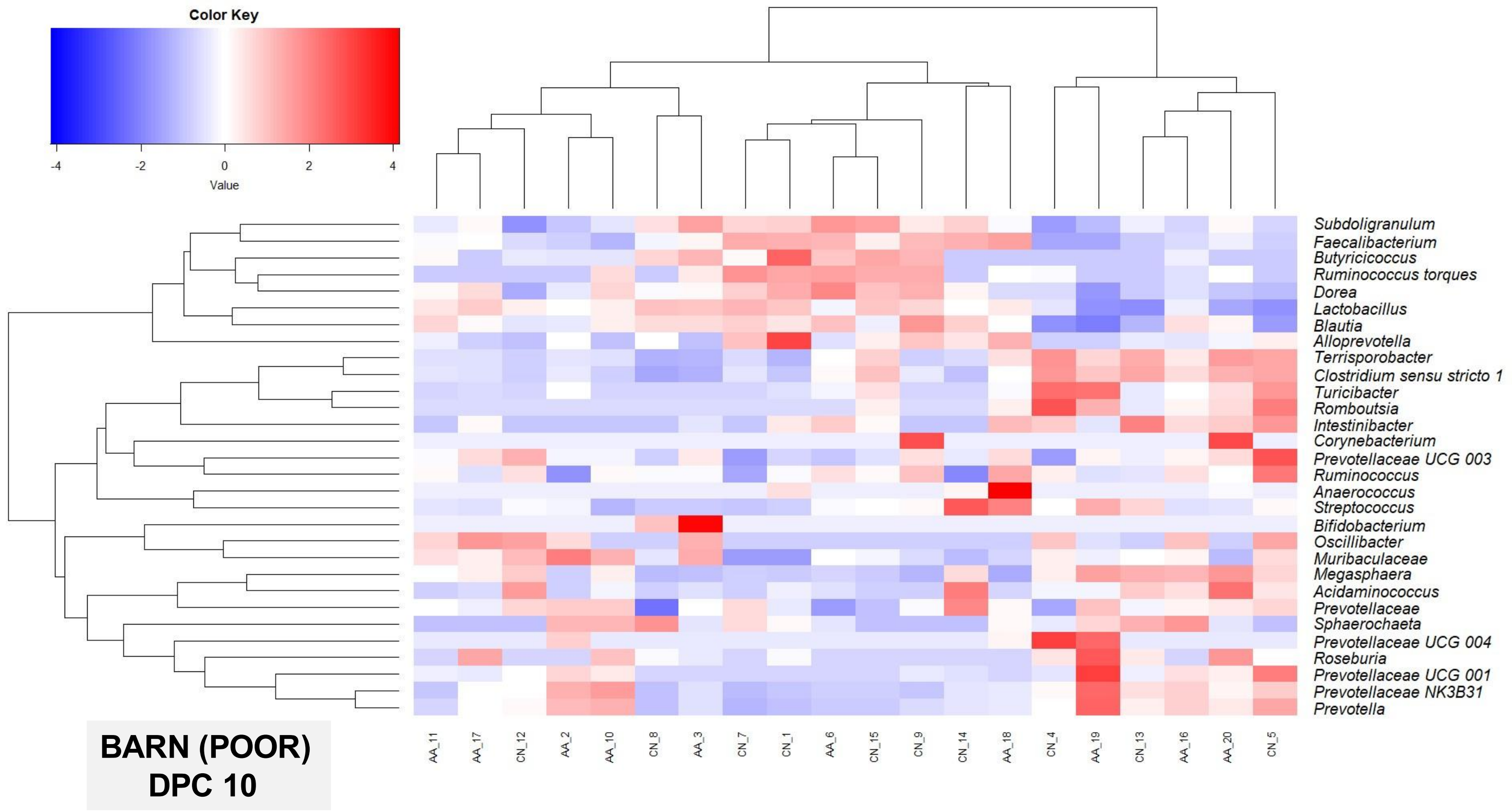

Supplementary Figure 39 | Heatmap clustering major taxa abundance in fecal microbiome of pigs that will be housed in a barn under POOR sanitary condition (SC) at DPC 10 and that were fed a control (CN) or a diet supplemented with functional amino acids supplemented (AA). Each row represents a distinct taxon, while each column represents an individual pig housed in a barn under POOR SC and that will feed a CN or AA diet over the experiment. The stronger red color, the more abundant the taxon is; while the stronger blue color, less abundant the taxon is. Log_2 rarefied transformed proportions were used as input data for scaling.

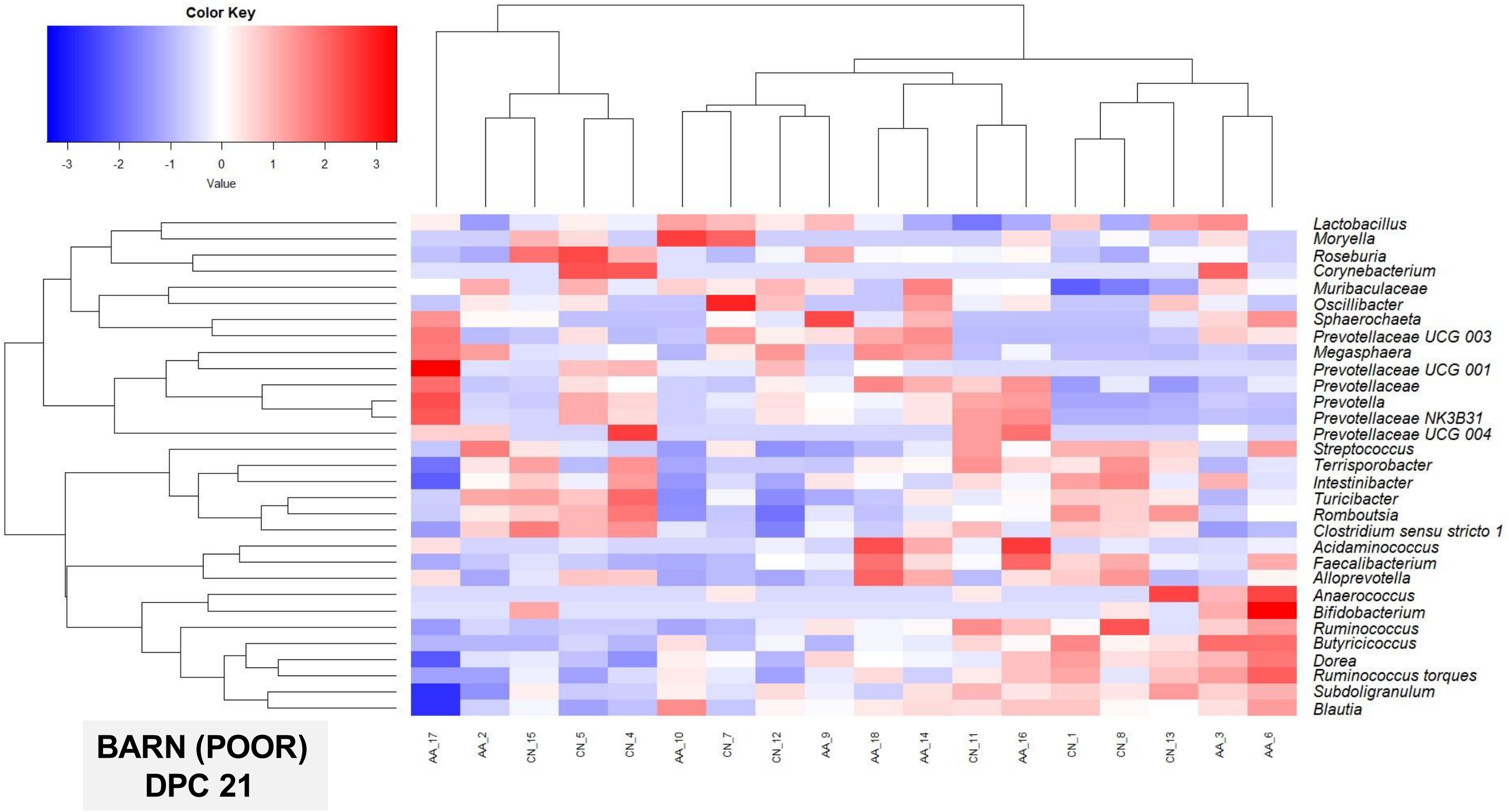

Supplementary Figure 40 | Heatmap clustering major taxa abundance in fecal microbiome of pigs that will be housed in a barn under POOR sanitary condition (SC) at DPC 21 and that were fed a control (CN) or a diet supplemented with functional amino acids supplemented (AA). Each row represents a distinct taxon, while each column represents an individual pig housed in a barn under POOR SC and that will feed a CN or AA diet over the experiment. The stronger red color, the more abundant the taxon is; while the stronger blue color, less abundant the taxon is. Log_2 rarefied transformed proportions were used as input data for scaling.

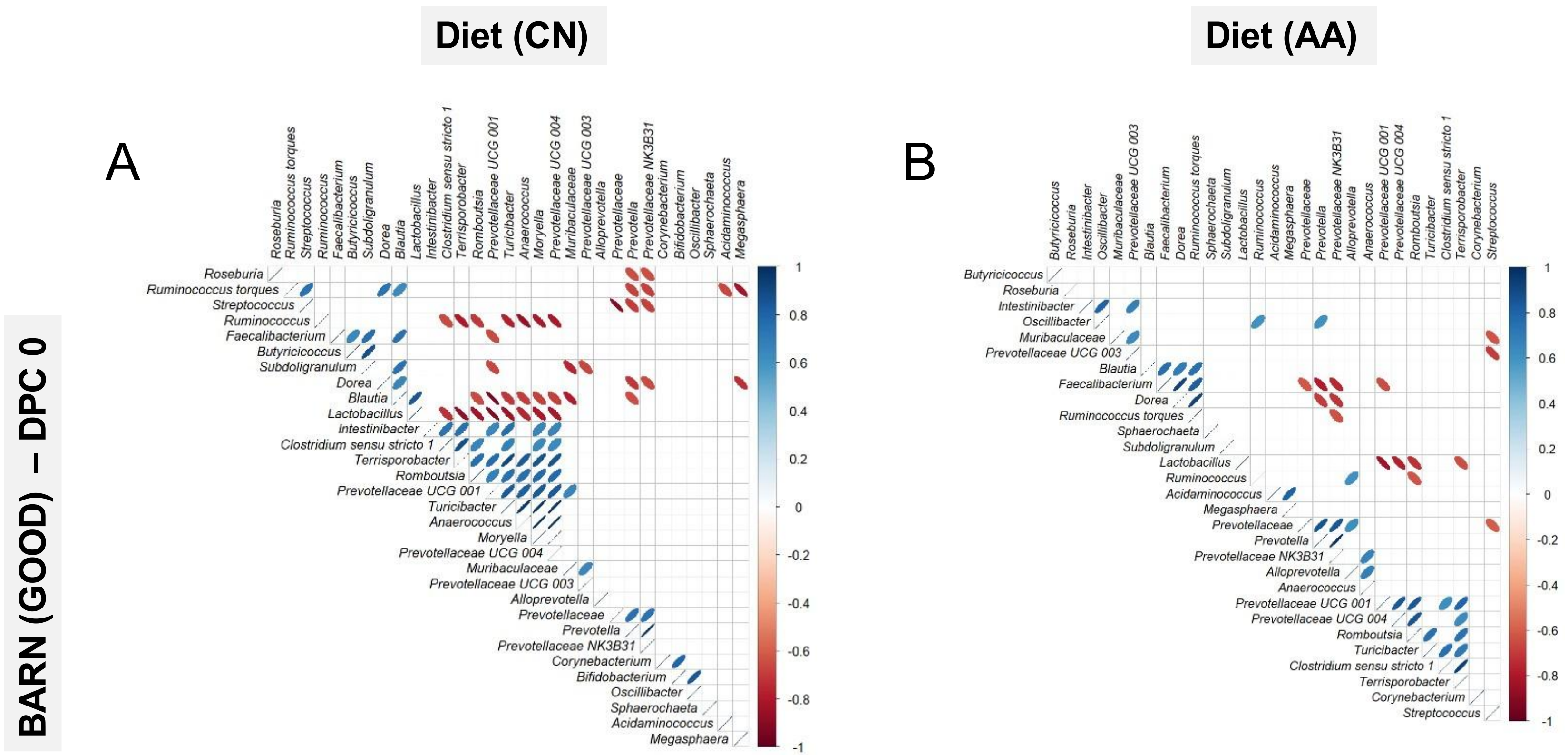

Supplementary Figure 41 | Correlation analysis between fecal microbiome taxa of pigs that will be housed in a barn under GOOD sanitary condition (SC) at DPC 0 and that were fed a control (CN) or a diet supplemented with functional amino acids (AA) (**A-B**). Blue ellipses indicate a positive correlation, while red ellipses indicate a negative correlation. The more circular the symbol, the weaker the correlation between taxa, while the straighter the symbol, the stronger the correlation between taxa. Only Pearson's correlations that passed the P < 0.05 cut-off were kept in the plot. Log_2 transformed rarefied proportions were used as input values.

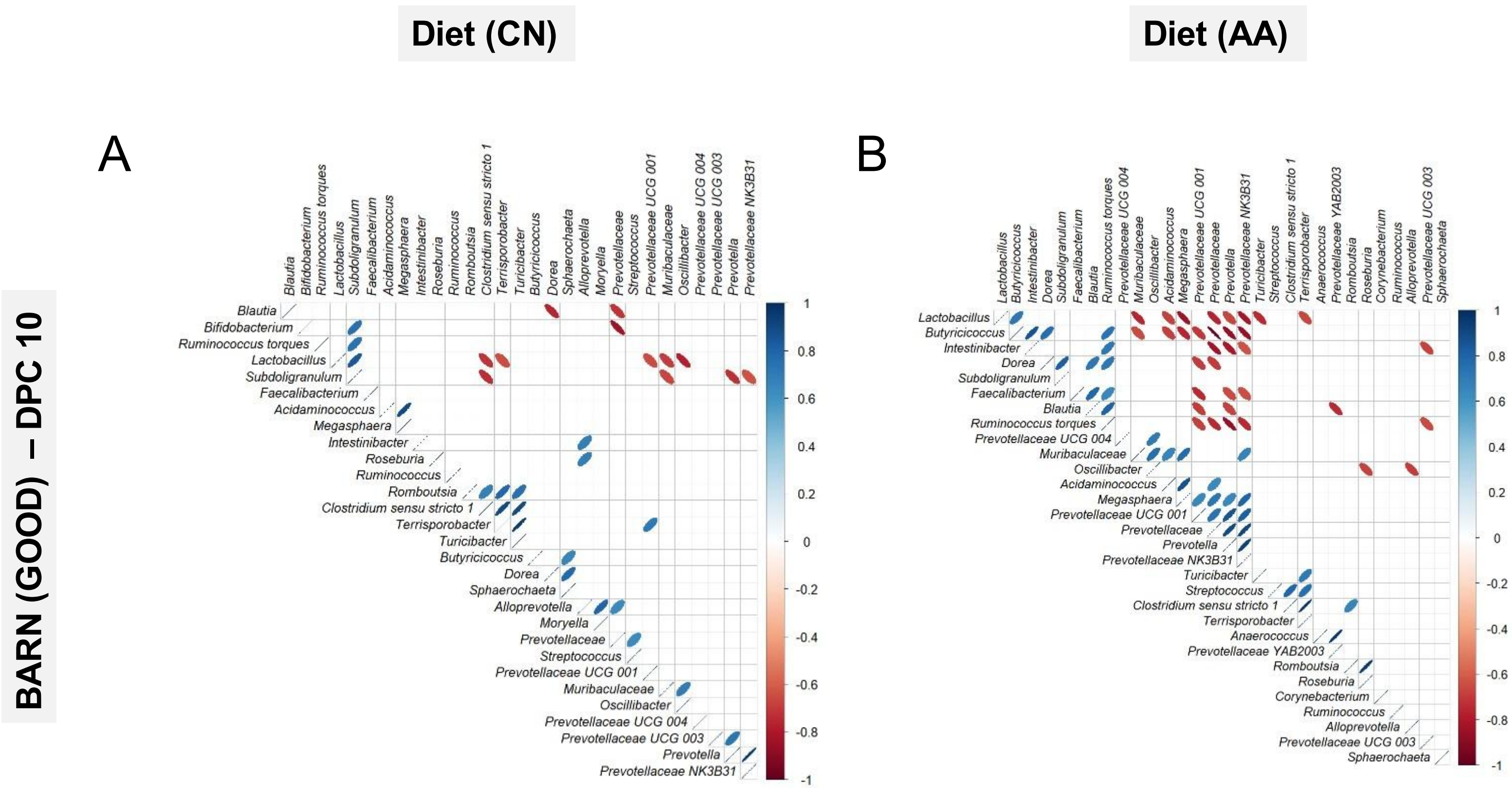

Supplementary Figure 42 | Correlation analysis between fecal microbiome taxa of pigs that will be housed in a barn under GOOD sanitary condition (SC) at DPC 10 and that were fed a control (CN) or a diet supplemented with functional amino acids (AA) (**A-B**). Blue ellipses indicate a positive correlation, while red ellipses indicate a negative correlation. The more circular the symbol, the weaker the correlation between taxa, while the straighter the symbol, the stronger the correlation between taxa. Only Pearson's correlations that passed the $P < 0.05$ cut-off were kept in the plot. Log_2 transformed rarefied proportions were used as input values.

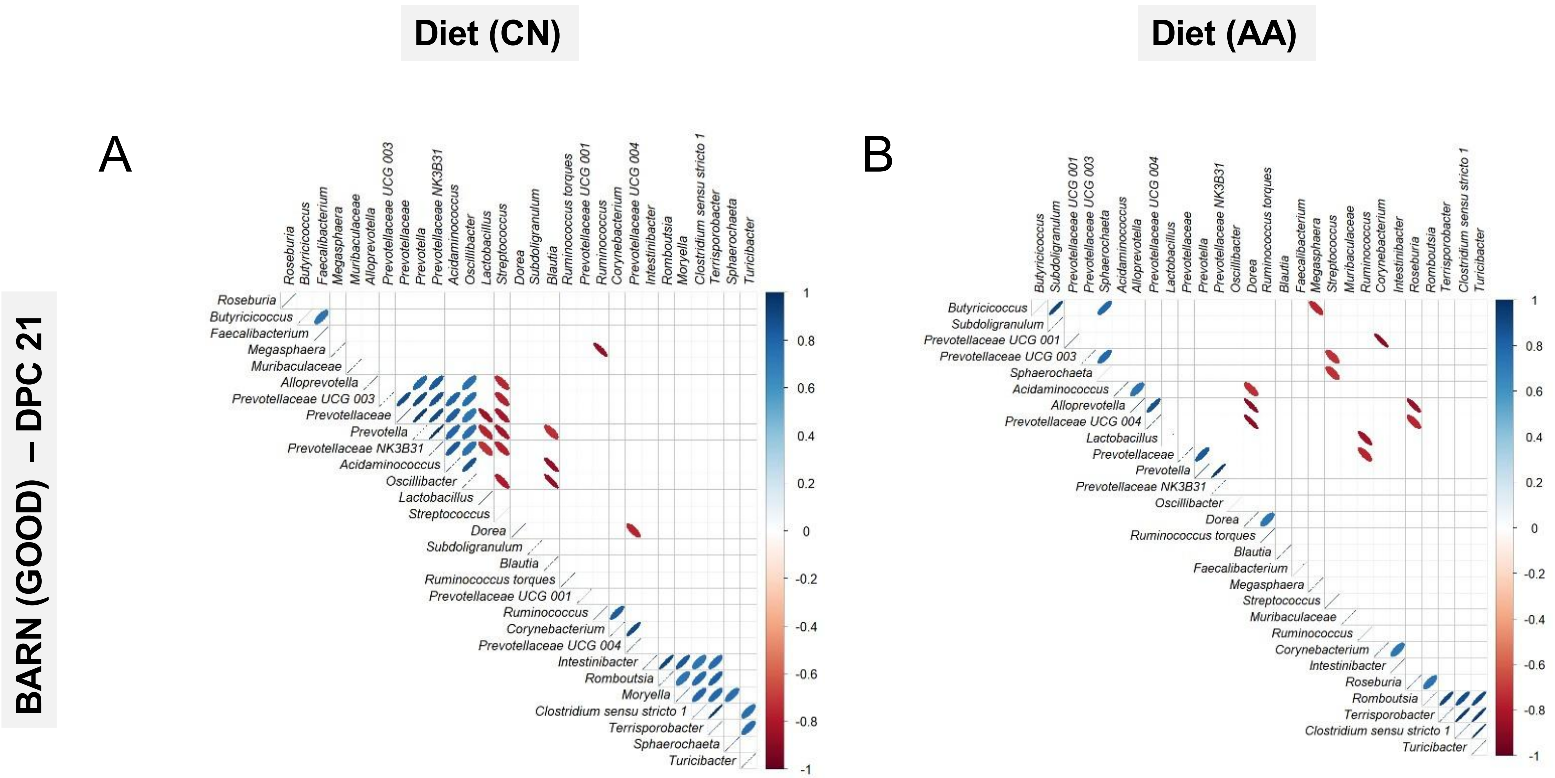

Supplementary Figure 43 | Correlation analysis between fecal microbiome taxa of pigs that will be housed in a barn under GOOD sanitary condition (SC) at DPC 21 and that were fed a control (CN) or a diet supplemented with functional amino acids (AA) (**A-B**). Blue ellipses indicate a positive correlation, while red ellipses indicate a negative correlation. The more circular the symbol, the weaker the correlation between taxa, while the straighter the symbol, the stronger the correlation between taxa. Only Pearson's correlations that passed the P < 0.05 cut-off were kept in the plot. Log_2 transformed rarefied proportions were used as input values.

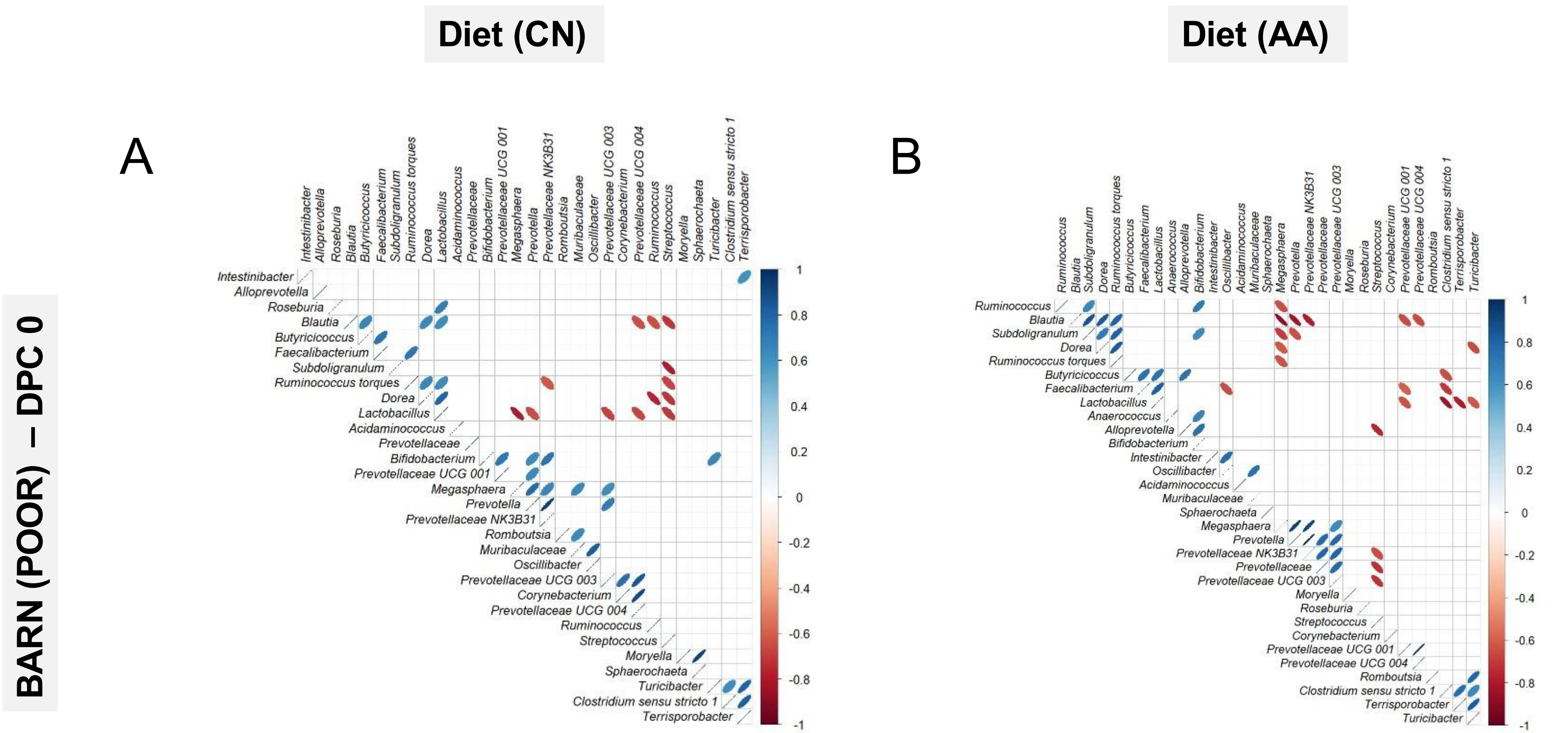

Supplementary Figure 44 | Correlation analysis between fecal microbiome taxa of pigs that will be housed in a barn under POOR sanitary condition (SC) at DPC 0 and that were fed a control (CN) or a diet supplemented with functional amino acids (AA) (**A-B**). Blue ellipses indicate a positive correlation, while red ellipses indicate a negative correlation. The more circular the symbol, the weaker the correlation between taxa, while the straighter the symbol, the stronger the correlation between taxa. Only Pearson's correlations that passed the $P < 0.05$ cut-off were kept in the plot. Log_2 transformed rarefied proportions were used as input values.

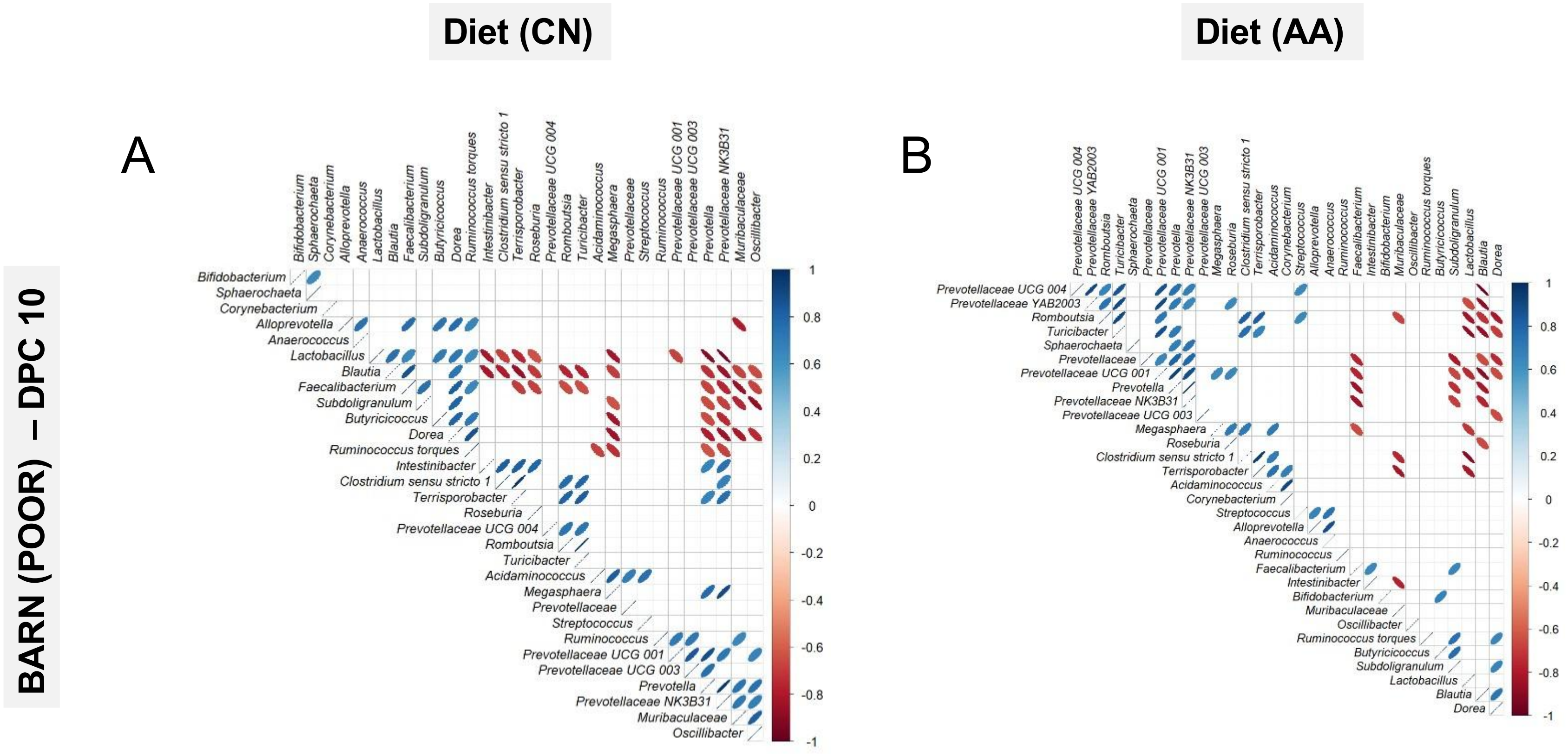

Supplementary Figure 45 | Correlation analysis between fecal microbiome taxa of pigs that will be housed in a barn under POOR sanitary condition (SC) at DPC 10 and that were fed a control (CN) or a diet supplemented with functional amino acids (AA) (**A-B**). Blue ellipses indicate a positive correlation, while red ellipses indicate a negative correlation. The more circular the symbol, the weaker the correlation between taxa, while the straighter the symbol, the stronger the correlation between taxa. Only Pearson's correlations that passed the $P < 0.05$ cut-off were kept in the plot. Log_2 transformed rarefied proportions were used as input values.

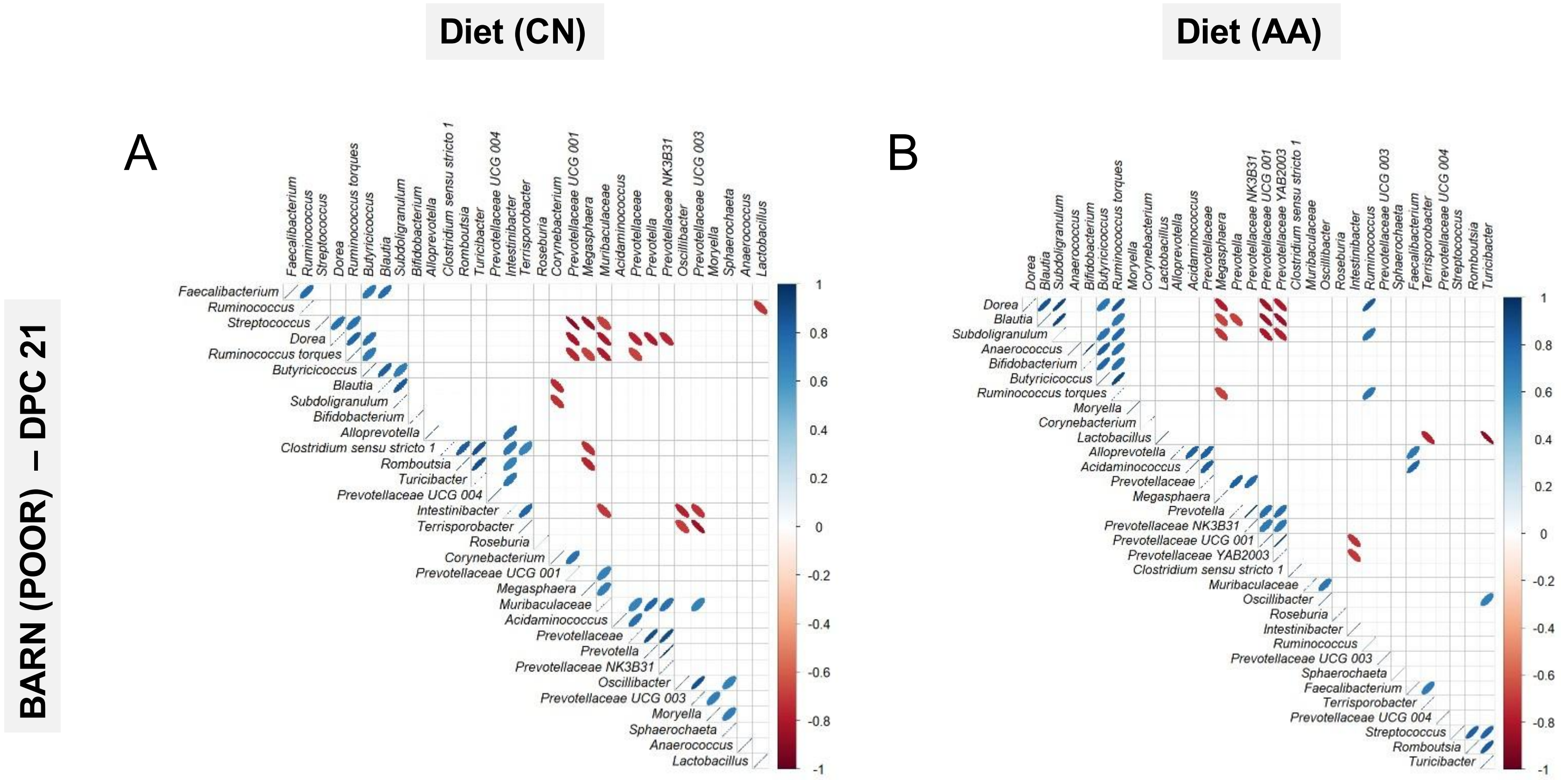

Supplementary Figure 46 | Correlation analysis between fecal microbiome taxa of pigs that will be housed in a barn under POOR sanitary condition (SC) at DPC 21 and that were fed a control (CN) or a diet supplemented with functional amino acids (AA) (**A-B**). Blue ellipses indicate a positive correlation, while red ellipses indicate a negative correlation. The more circular the symbol, the weaker the correlation between taxa, while the straighter the symbol, the stronger the correlation between taxa. Only Pearson's correlations that passed the $P < 0.05$ cut-off were kept in the plot. Log_2 transformed rarefied proportions were used as input values.
